# A Comparative Study on the Complexation of the Anticancer Iron Chelator VLX600 with Essential Metal Ions

**DOI:** 10.1021/acs.inorgchem.3c03259

**Published:** 2024-01-24

**Authors:** Vivien Pósa, Anja Federa, Klaudia Cseh, Dominik Wenisch, Gabriella Spengler, Nóra V. May, Norbert Lihi, Gergely F. Samu, Michael A. Jakupec, Bernhard K. Keppler, Christian R. Kowol, Éva A. Enyedy

**Affiliations:** † MTA-SZTE Lendület Functional Metal Complexes Research Group, University of Szeged, Dóm tér 7, H-6720 Szeged, Hungary; ‡ Department of Molecular and Analytical Chemistry, Interdisciplinary Excellence Centre, University of Szeged, Dóm tér 7-8, H-6720 Szeged, Hungary; § Institute of Inorganic Chemistry, Faculty of Chemistry, University of Vienna, Waehringer Strasse 42, A-1090 Vienna, Austria; ∥ Research Cluster “Translational Cancer Therapy Research”, Waehringer Strasse 42, A-1090 Vienna, Austria; ⊥ Department of Medical Microbiology, Albert Szent-Györgyi Health Center and Albert Szent-Györgyi Medical School, University of Szeged, Semmelweis utca 6, H-6725 Szeged, Hungary; # Centre for Structural Science, Research Centre for Natural Sciences, Hungarian Research Network (HUN-REN), Magyar tudósok körútja 2, H-1117 Budapest, Hungary; ∇ ELKH-DE Mechanisms of Complex Homogeneous and Heterogeneous Chemical Reactions Research Group, Department of Inorganic and Analytical Chemistry, University of Debrecen, Egyetem tér 1., H-4032 Debrecen, Hungary

## Abstract

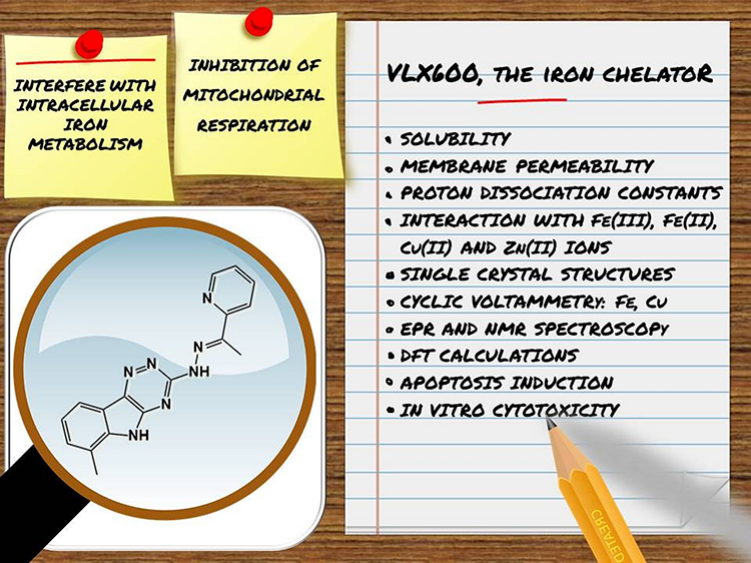

As cancer cells exhibit an increased uptake of iron, targeting the interaction with iron has become a straightforward strategy in the fight against cancer. This work comprehensively characterizes the chemical properties of 6-methyl-3-{(2*E*)-2-[1-(2-pyridinyl)ethylidene]hydrazino}-5*H*-[1,2,4]triazino[5,6-*b*]indole (VLX600), a clinically investigated iron chelator, in solution. Its protonation processes, lipophilicity, and membrane permeability as well as its complexation with essential metal ions were investigated using UV–visible, electron paramagnetic resonance, and NMR spectroscopic and computational methods. Formation constants revealed the following order of metal binding affinity at pH 7.4: Cu(II) > Fe(II) > Zn(II). The structures of VLX600 (denoted as HL) and the coordination modes in its metal complexes [Cu(II)(LH)Cl_2_], [Cu(II)(L)(CH_3_OH)Cl], [Zn(II)(LH)Cl_2_], and [Fe(II)(LH)_2_](NO_3_)_2_ were elucidated by single-crystal X-ray diffraction. Redox properties of the iron complexes characterized by cyclic voltammetry showed strong preference of VLX600 toward Fe(II) over Fe(III). *In vitro* cytotoxicity of VLX600 was determined in six different human cancer cell lines, with IC_50_ values ranging from 0.039 to 0.51 μM. Premixing VLX600 with Fe(III), Zn(II), and Cu(II) salts in stoichiometric ratios had a rather little effect overall, thus neither potentiating nor abolishing cytotoxicity. Together, although clinically investigated as an iron chelator, this is the first comprehensive solution study of VLX600 and its interaction with physiologically essential metal ions.

## Abstract 2

Solution speciation of VLX600 and its metal complexes

## Introduction

**Chart 1:**
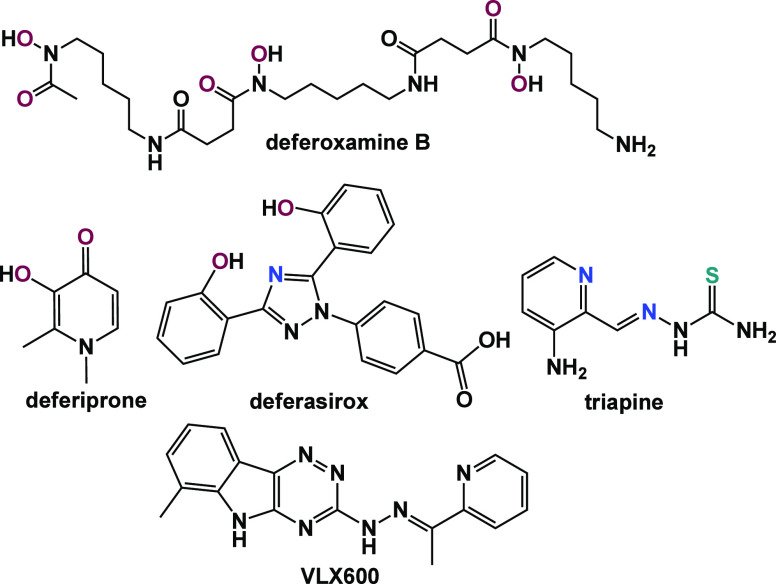
Chemical Formulae of Selected Iron Chelators: Deferoxamine B, Deferiprone, Deferasirox, Triapine, and VLX600

Despite significant advances in recent decades in the development of chemotherapeutic drugs with higher efficacy and tolerability, the treatment of cancer often continues to be encumbering because of adverse effects and multidrug resistance (MDR) developed by the tumor cells. These problems still account for the need to investigate novel therapies combining good efficiency and selectivity. Transition metal ions such as iron, copper, or zinc are vital micronutrients, and dysregulation of metal homeostasis contributes to the pathogenesis of many different types of cancer.^1^ These metal ions play a crucial role in the growth and proliferation of rapidly dividing cancer cells. It is evident that cancer cells demonstrate a higher requirement and preference for iron than normal tissue cells.^1−3^ In line with these facts, the application of iron-chelating compounds is one of the more traditional intervention strategies, and numerous ligands with iron binding capacity were screened for their anticancer properties with some promising results.^2−7^ Initially, iron chelators were developed to remove excess iron in the blood, mostly in the life-long medical therapy for β-thalassemia, in which metal overload is an unfortunate clinical consequence of repeated blood transfusions.^8−10^ Iron chelators such as deferoxamine B, deferiprone, or deferasirox (Chart 1) are hydrophilic ligands typically featuring hard Lewis base donor atoms (mostly O), which rather selectively bind Fe(III) ions in an octahedral arrangement.^9^ As a result, the redox potentials of the Fe(III)/Fe(II) complexes of these ligands are much lower (deferoxamine B: −450 mV, deferiprone: −620 mV, deferasirox: −400 mV vs NHE) compared to the Fe(III)/Fe(II) aqua/hydroxido complexes (+770 mV vs NHE at pH < 2.2; +380 mV vs NHE at pH = 7.4) or complexes of ligands with a strong tendency toward Fe(II) coordination (e.g., 1,10-phenantroline: +1130 mV, 2,2-bipiyridine: +1100 mV vs NHE).^10^ Therefore, the iron complexes of chelators applied in hematologic disorders are not involved in redox cycles under physiological conditions due to their low redox potential.^8−11^ A different behavior is observed with iron complexes of chelators binding both Fe(III) and Fe(II) such as α-*N*-heterocyclic thiosemicarbazones (TSCs), bearing an *(N,N,S)*-donor set.^2,3,5^ Notably, thiosemicarbazones are lipophilic molecules preferentially interacting with iron intracellularly. Their iron complexes show moderate redox potentials (varying TSCs: −160 to +40 mV vs NHE), enabling redox cycling.^12^ The most prominent representative is triapine (Chart 1), already studied in more than 30 clinical phase I–III trials, with the inhibition of the iron-containing enzyme ribonucleotide reductase as the main target.^3,5^

A novel, recently developed iron chelator is 6-methyl-3-{(2*E*)-2-[1-(2-pyridinyl)ethylidene]hydrazino}-5*H*-[1,2,4]triazino[5,6-*b*]indole (VLX600, Chart 1).^13^ It was designed to deplete iron levels and interfere with intracellular iron metabolism, leading to the inhibition of mitochondrial respiration. VLX600 demonstrated significant antitumor activity with a high therapeutic index both *in vitro* and *in vivo.*(13−15) Furthermore, it was also found that VLX600 decreases mitochondrial oxidative phosphorylation and induces a HIF-1α-dependent shift to glycolysis.^13^ A phase I study started in 2015 to determine its safety and adverse event profile along with the maximum tolerated dose (NCT02222363) in patients with refractory advanced solid tumors.^16,17^ It was well tolerated; however, no formal efficacy or survival analyses were performed, and the study was closed early because of slow recruitment.^16^

As a triazinoindolyl-hydrazone, VLX600 is able to bind metal ions via a tridentate *(N,N,N)* coordination mode. Although this compound has already been clinically studied as an iron chelator, comprehensive data (e.g., formation constants, stoichiometry, protonation state) on the interaction with Fe(III) and Fe(II) or the other biologically relevant metal ions Cu(II) and Zn(II) have not been reported so far. Only simple UV–vis spectra in the presence of several metal ions have been published.^14^ Density functional theory (DFT) calculations suggested that in the Fe(II) bis-ligand complex, VLX600 is coordinated in a tridentate manner via the nitrogen atoms of its pyridine, hydrazine, and 1,2,4-triazine moiety. The Fe(II) and Fe(III) bis complexes were obtained in the solid phase, and extended X-ray absorption fine structure (EXAFS) spectroscopy confirmed the pseudo-octahedral geometry with short Fe–N bond distances suggesting the formation of low-spin complexes.^14^ However, no X-ray single-crystal structures have been reported so far.

In the present comparative study, we investigated the proton dissociation processes of VLX600 and its complex formation reactions with Fe(III), Fe(II), Cu(II), and Zn(II) ions. In addition, we provide a complete overview on the solution speciation and structures in addition to the electrochemical properties of the iron and copper complexes of VLX600, as well as the cytotoxic activities in various human cancer cell lines also in the presence of these metal ions.

## Results and Discussion

### Synthesis, Proton Dissociation Processes, and Membrane Permeability of VLX600

**Figure 1:**
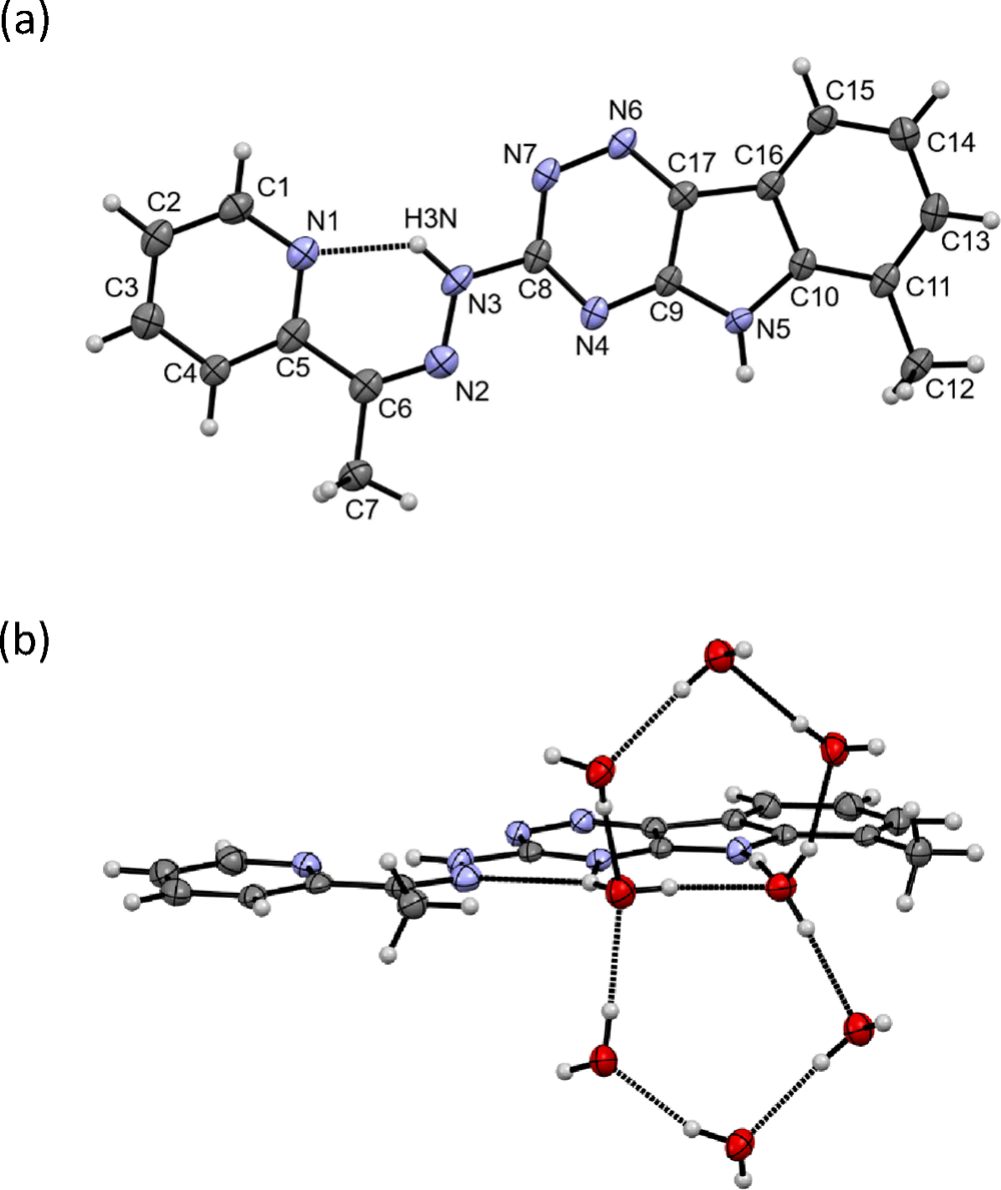
(a) X-ray crystal structure of VLX600 as HL·3H_2_O (the water molecules are omitted for clarity). Selected bond lengths (Å) and bond and torsion angles (°): C6–N2 1.311(3), N2–N3 1.374(3), N3–C8 1.380(3), C8–N4 1.335(3), C8–N7 1.353(3), N7–N6 1.363(3) Å; ∠C5–C6–N2 127.2(2), C6–N2–N3 119.1(2), N2–N3–C8 118.0(2), N3–C8–N7 113.1(2), N3–C8–N4 118.4(2)°; ∠C5–C6–N2–N3 0.91, N2–N3–C8–N7 177.76, N2–N3–C8–N4 1.45°. (b) Co-crystallized water molecules forming infinite pentagonal ring system chains stabilized by several hydrogen bonds to the VLX600 ligand (two pentagons are depicted).

VLX600 was synthesized according to literature procedures.^18^ The commercially available 6-methyl-4,5-dihydro-3*H*-[1,2,4]triazino[5,6-*b*]indole-3-thione was reacted with hydrazine monohydrate, yielding 3-hydrazinyl-6-methyl-5*H*-[1,2,4]triazino[5,6-*b*]indole, which was further reacted with 2-acetylpyridine in a H_2_O/ethanol (EtOH) mixture at 90 °C. The subsequent condensation reaction produced VLX600 in excellent yield (94%). The structure of VLX600 was confirmed by ^1^H and ^13^C NMR spectroscopy, elemental analysis, high-resolution mass spectrometry measurements, and, for the first time, X-ray crystallography (Figure 1a). X-ray diffraction quality single crystals were grown via vapor diffusion of ethyl acetate (EtOAc) into methanol (MeOH) and crystallized in the monoclinic *C*2/*c* space group. Selected bond distances (Å) and angles are quoted in the legend to Figure 1. VLX600 adopts a planar conformation with a hydrogen bond between the pyridine N1 and the hydrazonic NH forming a *cis*-configuration of the C5/N3 atoms. Such a hydrogen bond stabilization is also typical for *Z*-isomers of α-*N*-pyridyl thiosemicarbazones.^19^ The N2 and N4 atoms are in a *cis* configuration. It was found that the surrounding co-crystallized water molecules form pentagonal ring systems (Figure 1b), stabilized by several hydrogen bonds to the VLX600 ligand. Of note, two sets of isomers could be observed in the ^1^H NMR spectra in deuterated dimethyl sulfoxide (DMSO)-*d*_6_. As the main species (∼90%), the E-isomer with the hydrazonic NH at 10.89 ppm and, as the minor species, the Z-isomer (∼10%) with the hydrazonic NH at 14.88 ppm are seen, clearly indicating the involvement of a hydrogen bond in agreement with the X-ray crystal structure. The two isomers are in equilibrium, and after 6 days at room temperature, the amount of the Z-isomer increased to ∼30%.

**Figure 2:**
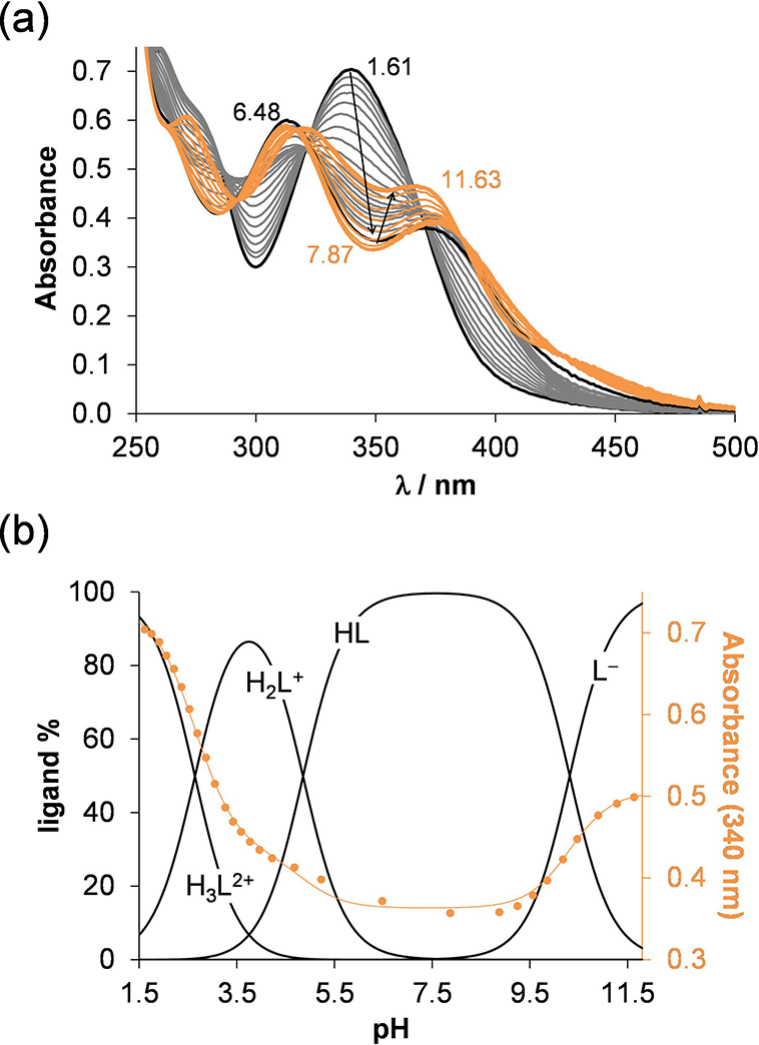
(a) UV–vis spectra of VLX600 recorded in the pH range between 1.6 and 12 in 30% (v/v) DMSO/H_2_O. (b) Concentration distribution curves calculated with the determined p*K*_a_ values and absorbance values at 340 nm (●) with the fitted line (solid line) {*c*_VLX600_ = 24.7 μM; *I* = 0.10 M KCl; *l* = 1 cm; *t* = 25.0 °C}.

**Figure 3:**
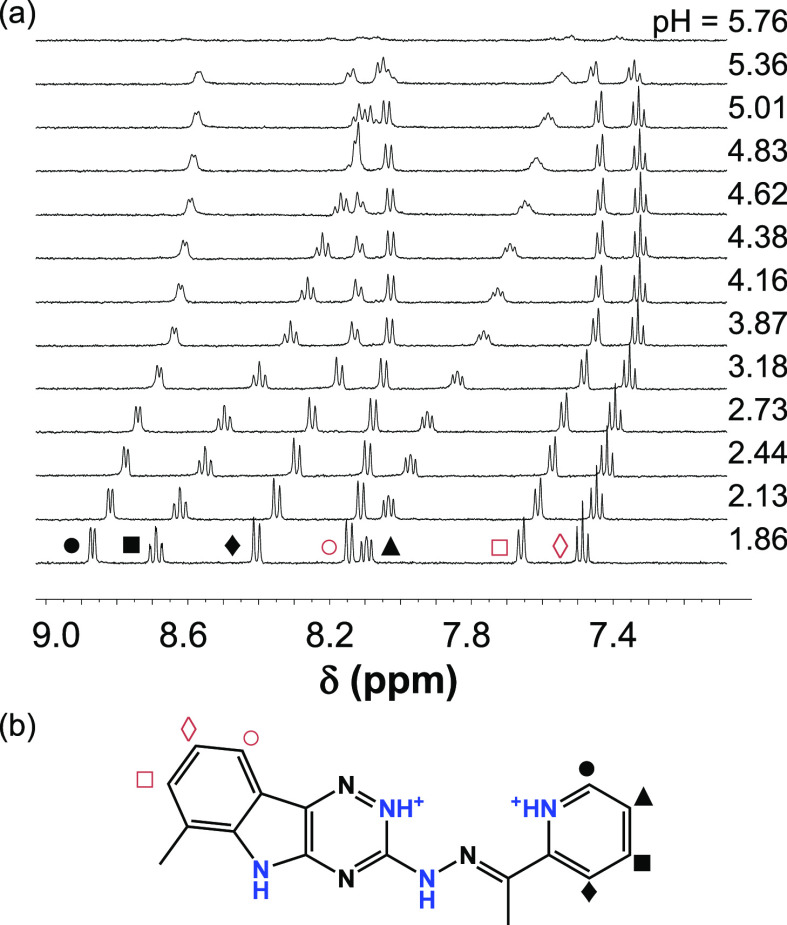
(a) ^1^H NMR spectra of VLX600 recorded at various pH values in the low-field region (7.1–9.0 ppm). (b) VLX600 in its doubly protonated state (H_3_L^2+^) and peak assignation with symbols {*c*_VLX600_ = 1 mM; 30% (v/v) DMSO-*d*_6_/H_2_O, *I* = 0.1 M KCl}.

VLX600 has limited aqueous solubility that hindered the use of pH-potentiometry for studying its (de)protonation processes. Therefore, UV–visible (UV–vis) spectrophotometric titrations were performed at a low concentration (25 μM) in a 30% (v/v) DMSO/H_2_O solvent mixture (UV–vis stability data over 48 h revealed a very slow precipitation process; Figure S1). The fully protonated form of VLX600 has four dissociable groups, namely, the pyridinium nitrogen (NH^+^), N^2^H^+^ of the 1,2,4-triazine moiety, and the hydrazone (NH) and indole (NH) nitrogens. The UV–vis spectra recorded for VLX600 at various pH values (Figure 2a) revealed three deprotonation processes in the studied pH range (1.6–12.0); thus, three proton dissociation constants could be computed (see p*K*_a_ values in Table 1). To assign the p*K*_a_ values to each dissociable functional group, ^1^H NMR spectroscopic titration was also performed (Figure 3a). Because of the formation of precipitate, the ^1^H NMR spectra could be recorded only up to pH ∼6 at the applied 1 mM concentration. In the monitored pH range, two deprotonation processes were observed, and the signals of all the aromatic CH protons were sensitive to the changes in pH (Figure 3 and Figure S2). However, the peaks of the pyridine and indole moieties were shifted differently. The signals of the indole protons were downfield shifted up to pH 4 and then remained unchanged, whereas the peaks of the pyridine ring protons continued to change even at pH > 4. Based on these findings, the two lower p*K*_a_ values most probably belong to the pyridinium and N^2^H^+^ nitrogen atoms of the 1,2,4-triazine moiety, respectively. The highest p*K*_a_ most likely is attributed to the hydrazone (NH), whereas the deprotonation of the indole (NH) nitrogen occurs at pH > 12, which is beyond the pH range applied here. Therefore, the protonated form of VLX600 is denoted as H_3_L^2+^ (Figure 3b). Based on the obtained p*K*_a_ values, concentration distribution curves were computed (Figure 2b), revealing that the neutral HL is the sole species at pH 7.4.

**Table 1:** Overall Protonation Constants (Log β) and p*K*_a_ Values of VLX600 Determined by UV-Vis Titrations in 30% (v/v) DMSO/H_2_O in Addition to the λ_max_ and Molar Absorbance (ε) Values of the Ligand Species in Different Protonation States^a^

	**H**_**3**_**L**^**2+**^	**H**_**2**_**L**^**+**^	**HL**	**L**^**–**^
log β	17.81 ± 0.01	15.17 ± 0.01	10.32 ± 0.01	
**p***K*_**a**_	2.64	4.86	10.32	
**λ**_**ma**x_ (nm)/**ε** (M^–1^cm^–1^)	340/29715	321/21,973	313/23,997	322/23,680
		371/16,922	371/15,643	367/19,207

a HL denotes the neutral form of VLX600, and the equilibrium processes associated with the different constants are given in Table S1 {*I* = 0.1 M KCl; *t* = 25.0 °C}.

The lipophilicity and membrane permeability are key parameters for a drug as these properties strongly affect the absorption processes and bioavailability. We attempted to determine the distribution coefficients (*D*_7.4_) of VLX600 at pH 7.4 with the shake-flask method in *n*-octanol/buffered aqueous solution using 1:10 volume ratios. Unfortunately, the lipophilicity was so high that only a threshold limit could be estimated (log*D*_7.4_ > +2.8) as almost all VLX600 remained in the *n*-octanol phase. In parallel, an artificial membrane permeability assay (PAMPA) was performed to characterize the ability of VLX600 to penetrate membranes by passive diffusion. The determined effective passive permeability value (*P*_effective_ = 2.6 (±0.1) × 10^–6^ cm·s^–1^) reflects high cell membrane permeability.

### Complexation of VLX600 with Fe(III) and Fe(II) Ions

**Figure 4:**
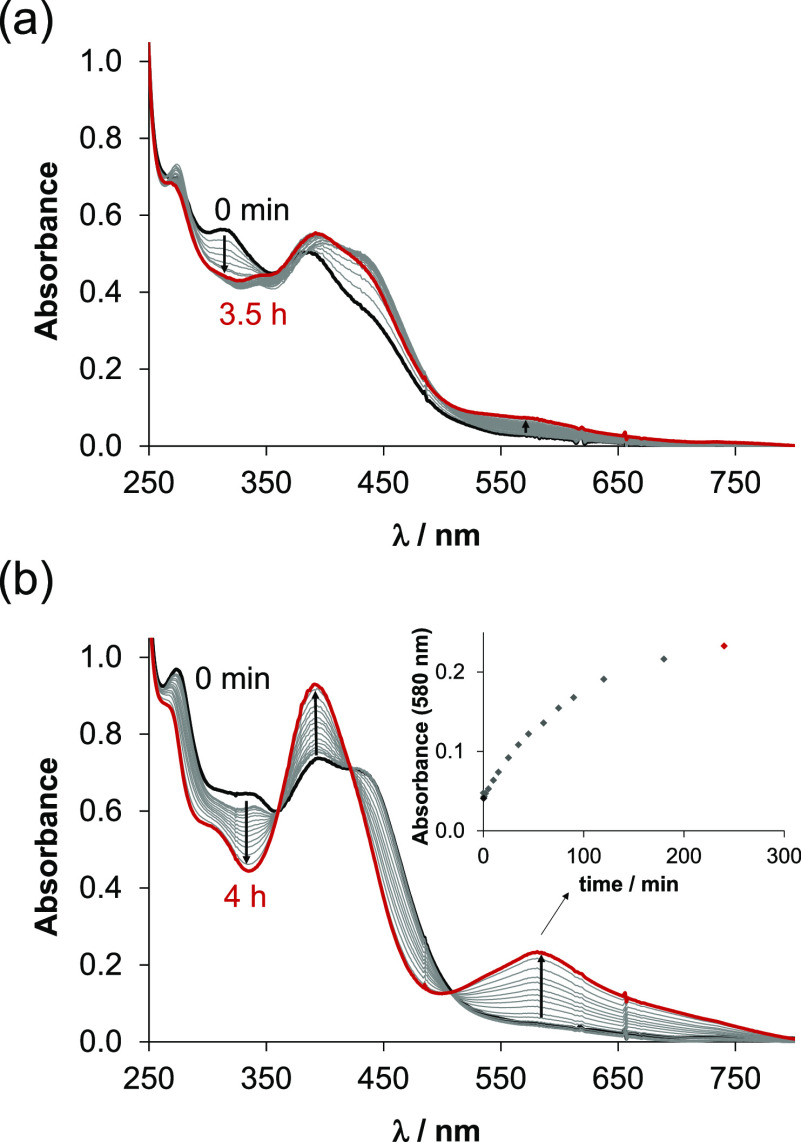
Time dependence of the UV–vis spectra of the Fe(III)–VLX600 (1:2) chemical system recorded at (a) pH 6 (50 mM 2-(*N*-morpholino)ethanesulfonic acid (MES)) and (b) pH 7.4 (50 mM *N*-2-hydroxyethylpiperazine-*N*-2-ethanesulfonic acid (HEPES)) in a 30% (v/v) DMSO/H_2_O solvent mixture. In panel b, the inset shows the absorbance values at 580 nm (◆) plotted against time {*c*_VLX600_ = 40 μM; *c*_Fe(III)_ = 20 μM; *I* = 0.10 M KCl; *l* = 1 cm; *t* = 25.0 °C}.

As VLX600 is considered as an iron chelator, its interaction with Fe(III) was first monitored by UV–vis spectrophotometry in 30% (v/v) DMSO/H_2_O solution at pH 7.4 and 6.0 (Figure 4). The recorded spectra indicate complex formation; however, they also show changes over time, which are occurring faster at pH 7.4. It is reasonable to assume that a redox reaction takes place resulting in increased absorbance values at ∼580 nm. The redox reaction is not complete under the conditions used, but the novel band is typical for Fe(II) complexes (Figure S3). A similar behavior was reported for the Fe(III)–1,10-phenantroline or Fe(III)–2,2′-bipyridine chemical systems due to the fact that those chelators prefer Fe(II) rather than Fe(III).^20,21^ For these ligands, the Fe(III) complex is reduced by OH^–^ with first-order kinetics, implying that the reaction rate is directly proportional to the concentration of OH^–^.^21^ Thus, a higher reaction rate for the reduction of the Fe(III)-VLX600 complex at pH 7.4 is not surprising. This phenomenon precluded the determination of the formation constants for the Fe(III)-VLX600 complexes. It is noteworthy that the bis-ligand Fe(III) complex of VLX600 was found to be stable in solid form, although no specific synthetic details have been reported.^14^

**Figure 5:**
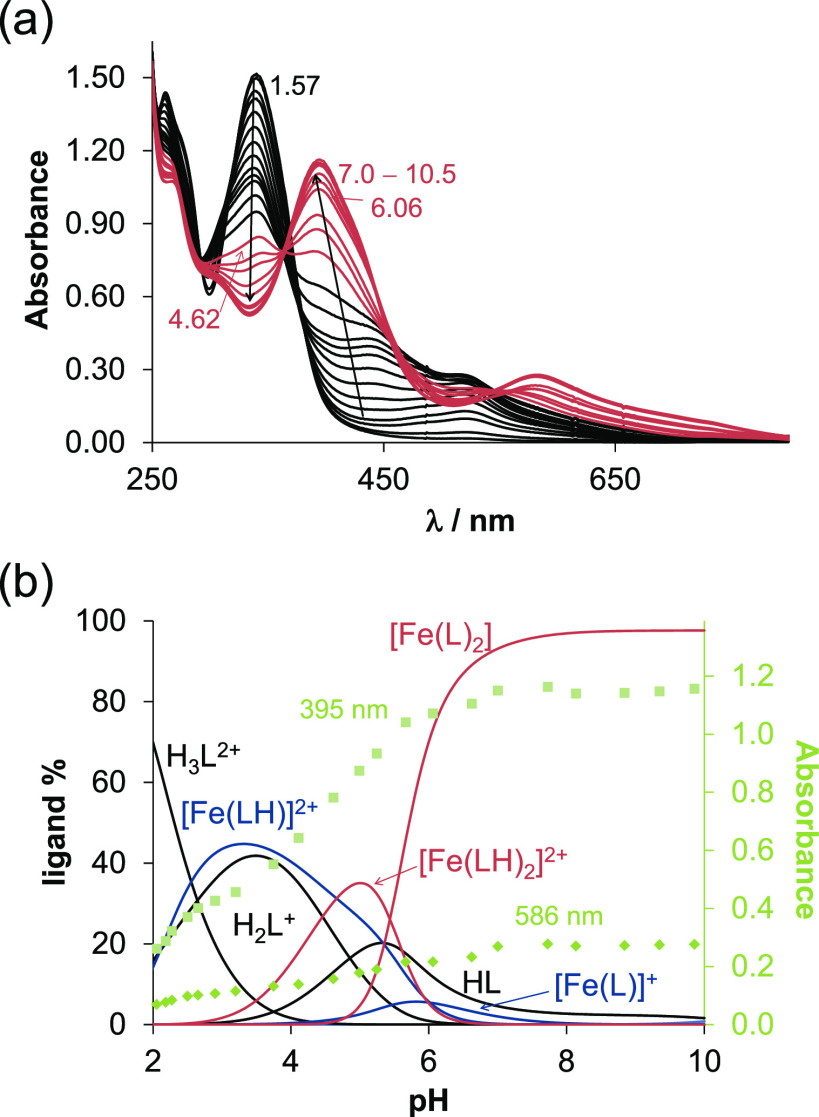
(a) UV–vis spectra of the Fe(II)–VLX600 (1:2) system recorded in the pH ranges between 1.6 and 10.5 in 30% (v/v) DMSO/H_2_O in a laboratory glovebox under strictly anaerobic conditions. (b) Concentration distribution curves computed with the determined formation constants (log β) and absorbance values at 395 nm (■) and 586 nm (◆) {*c*_VLX600_ = 50 μM; *c*_Fe(II)_ = 25 μM; *I* = 0.10 M KCl; *l* = 0.5 cm; *t* = 25.0 °C}.

Next, the Fe(II)–VLX600 chemical system was investigated under anaerobic conditions (in a laboratory glovebox) at various pH values as well as at different metal-to-ligand ratios (exemplary spectra shown in Figure 5a), and overall stability constants (β) for mono and bis complexes were determined in different protonation states (Table 2; the corresponding equilibrium processes are given in Table S1). In all complexes, coordination of the ligand is suggested to occur via the *(N,N,N)-*donor motif. In the complexes [Fe(LH)]^2+^ and [Fe(LH)_2_]^2+^, LH denotes the neutral form of the ligand, and the proton is attributed to the hydrazone nitrogen. Based on the concentration distribution diagrams (Figure 5b, Figure S4), it can be concluded that the neutral [Fe(L)_2_] bis complex is the dominant species at pH 7.4 with an absorption maximum at 586 nm yielding the characteristic greenish yellow color. These findings clearly indicate the binding preference of VLX600 for Fe(II) over Fe(III).

**Table 2:** Overall Stability (Formation) Constants (Log β) of the Complexes Formed with VLX600 Determined by UV–Vis Titrations in 30% (v/v) DMSO/H_2_O^a^

**log β**	**Fe(II)**	**Cu(II)**	**Zn(II)**
[M(LH)]^2+^	18.84 ± 0.01	20.63 ± 0.07	17.37 ± 0.06
[M(L)]^+^	14.31 ± 0.01	16.36 ± 0.07	10.51 ± 0.06
[M(L)H_–1_]		5.81 ± 0.07	2.53 ± 0.06
[M(LH)_2_]^2+^	33.91 ± 0.06		33.22 ± 0.06
[M(L)(LH)]^+^			26.41 ± 0.03
[M(L)_2_]	24.00 ± 0.03		17.97 ± 0.05

a The corresponding equilibrium processes to which the formation constants apply are given in Table S1. The coordinated solvent molecules are not labeled for simplicity {*I* = 0.1 M KCl; *t* = 25.0 °C}.

**Figure 6:**
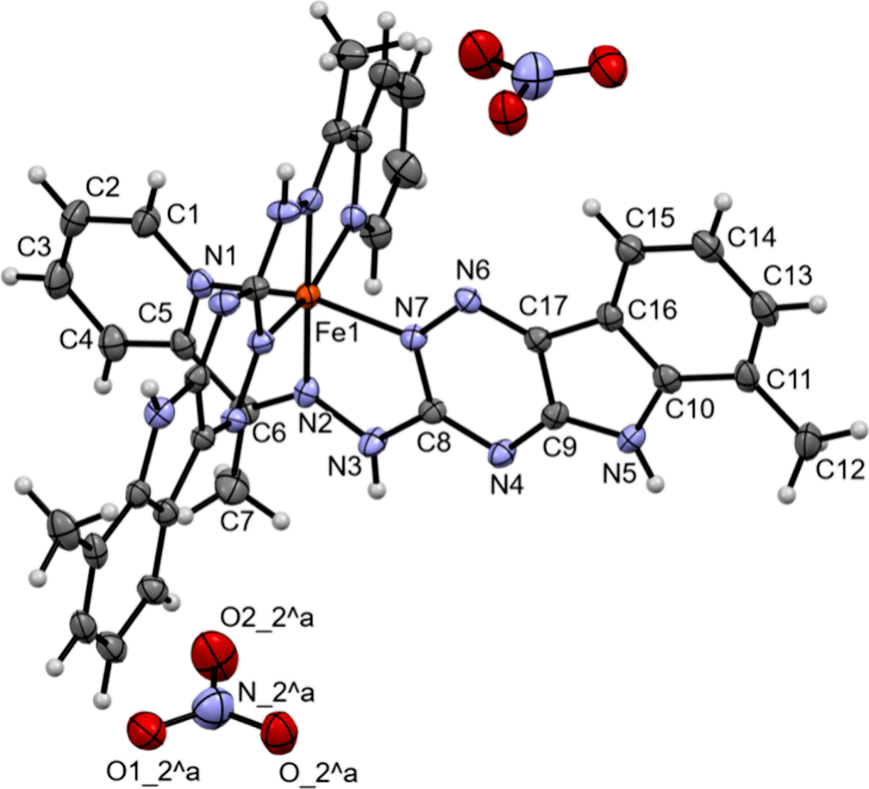
X-ray crystal structure of [Fe(II)(LH)_2_](NO_3_)_2_ (the water molecules are omitted for clarity). Because of the symmetry of the space group, the two VLX600 ligands have identical binding parameters. Selected bond lengths (Å) and bond and torsion angles (°): Fe–N1 1.9614(18), Fe–N2 1.8814(19), Fe–N7 1.9288(18), C6–N2 1.309(3), N2–N3 1.366(2), N3–C8 1.366(3), C8–N4 1.341(3), C8–N7 1.357(3), N7–N6 1.359(2) Å; ∠N1–Fe–N2 80.51(8), N2–Fe–N7 81.33(8), N1–Fe–N7 161.82(8), C5–C6–N2 110.2(2), C6–N2–N3 122.9(2), ∠N2–N3–C8 113.78(19), N3–C8–N7 114.6(2), N3–C8–N4 118.2(2)°; ∠C5–C6–N2–N3 179.76(17), N2–N3–C8–N7 2.7(3), N2–N3–C8–N4 176.17(18)°.

To investigate the coordination mode in the iron complexes and the possible redox reaction that takes place between Fe(III) and VLX600, the compound was mixed with Fe(NO_3_)_3_·9 H_2_O in a 2:1 ratio in EtOH for 3 h at 50 °C, and the formed solid powder was collected after 3 days at 4 °C for X-ray diffraction and X-ray photoelectron spectroscopy (XPS) studies. Crystals were grown from a methanolic solution of the powder under aerobic conditions with slow diffusion of ethyl acetate, generating X-ray diffraction quality crystals (Figure 6). The iron complex crystallized in the monoclinic *C*2/*c* space group and forms an octahedral system in which two planar, neutral VLX600 molecules are bound in a tridentate *(N,N,N)* mode to the metal ion via pyridine, imine N, and the N^2^ of the 1,2,4-triazine (denoted as N7 in Figure 6). Thus, in contrast to the free VLX600 ligand with hydrogen bond-stabilized *cis* C5/N3 atoms (Figure 1), in the iron complex, these atoms are in *trans* position concomitant with a *cis* N2/N7 configuration. Because of the symmetry of the space group, the two VLX600 ligands have identical binding parameters. More details are provided in the SI (Table S2). Of note, despite using Fe(III) for crystal growth, the respective Fe(II) complex [Fe(II)(LH)_2_](NO_3_)_2_ with two nitrate counterions was formed, indicating a very high preference for Fe(II) (in the case of Fe(III) and two nitrates, one of the ligands would have to be protonated, and thus, the binding parameters would no longer be the same). This binding mode was also suggested by DFT calculations;^14^ however, the formation of coordination isomers was not considered in these calculations (N^2^ or N^4^ coordination modes). Therefore, the geometry of low-spin iron(II) complexes was optimized, and the relative energies between the coordination isomers were compared. These DFT calculations confirmed that iron(II) favors the pyridine, imine N, and the N^2^ coordination mode over the pyridine, imine N, and the N^4^ donor set (see details in SI, Figures S5 and S6 and Tables S3–S7). This is in good agreement with the results of X-ray studies and further confirms the binding mode of the iron(II) complex in solution.

**Figure 7:**
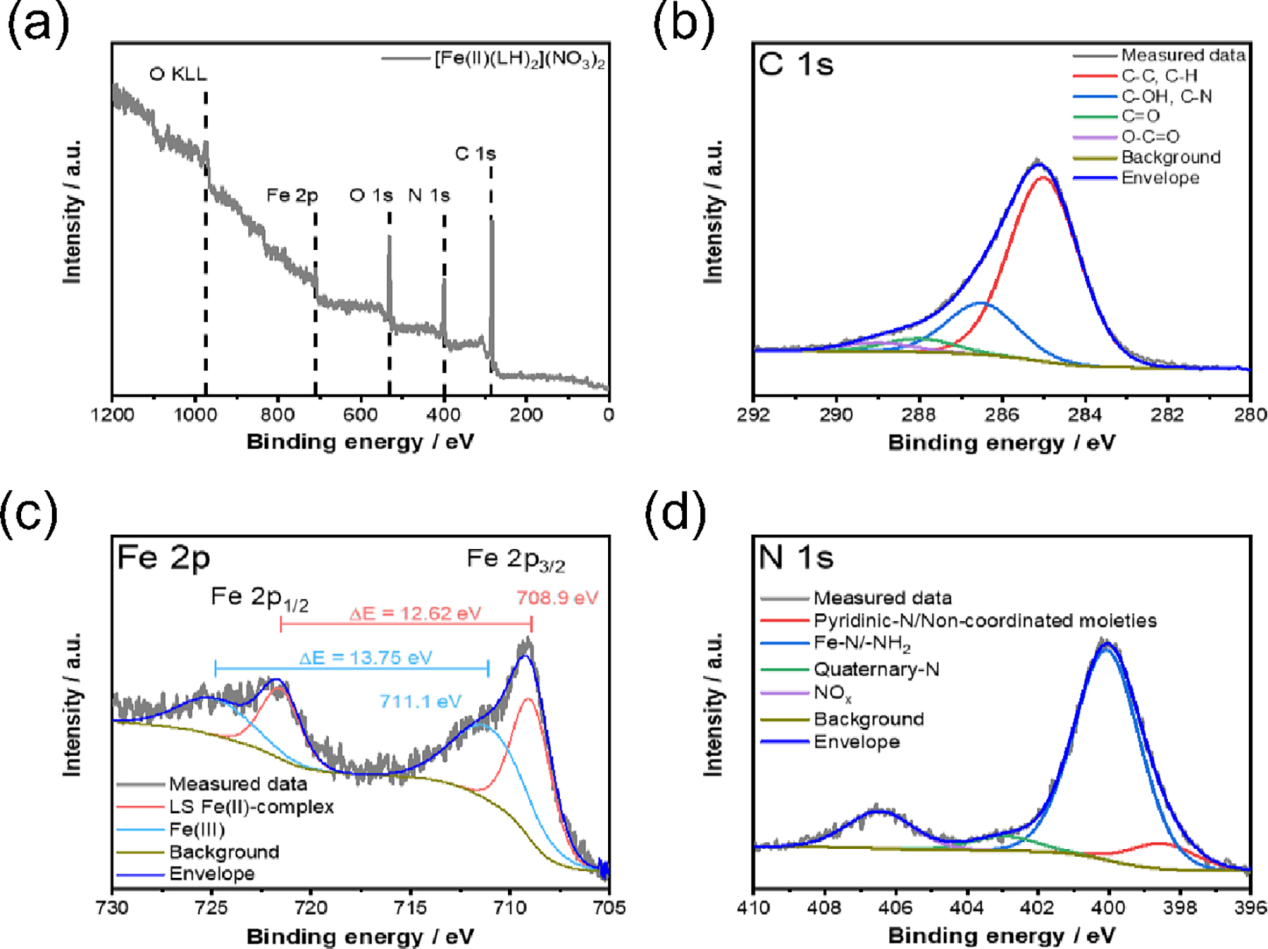
(a) XPS survey scan of the iron complex of VLX600. High-resolution core-level spectra of (b) C 1s, (c) Fe 2p, and (d) N 1s regions.

To further investigate the oxidation and spin state of the iron complex, the solid iron-VLX600 powder was measured by XPS (Figure 7); however, this method provides information only about the surface of the solid material. The recorded survey scans reveal the presence of only C, O, N, and Fe (Figure 7a). The high-resolution Fe 2p region (Figure 7c) could be fitted with two components. The lower binding energy component (2p_3/2_ = 708.9 eV) could be assigned to a low-spin Fe(II) complex, indicating that the reduction of Fe(III) has indeed occurred. This assignment is based on (i) the narrow peak of the core–electron line and (ii) the peak separation of the 2p_3/2_-2p_1/2_ lines (Δ*E* ∼ 12.5 eV for low-spin Fe(II) moieties).^22^ As low-spin Fe(II) complexes have no satellite features at higher binding energies, the other component in the Fe 2p spectra was assigned to an Fe(III)-containing complex. This can be formed through the oxidation at the surface of the powder (as the sample was stored in air) as also reported in the literature.^22,23^ It is noteworthy that the multiplet splitting of high-spin Fe(II) compounds can also have higher binding energy features; however, their position is typically located at even higher binding energies of 2.5–7.5 eV. The N 1s region is dominated by the signal arising from the Fe–N binding in the complex (400.1 eV), in good agreement with literature examples.^22,24^

**Figure 8:**
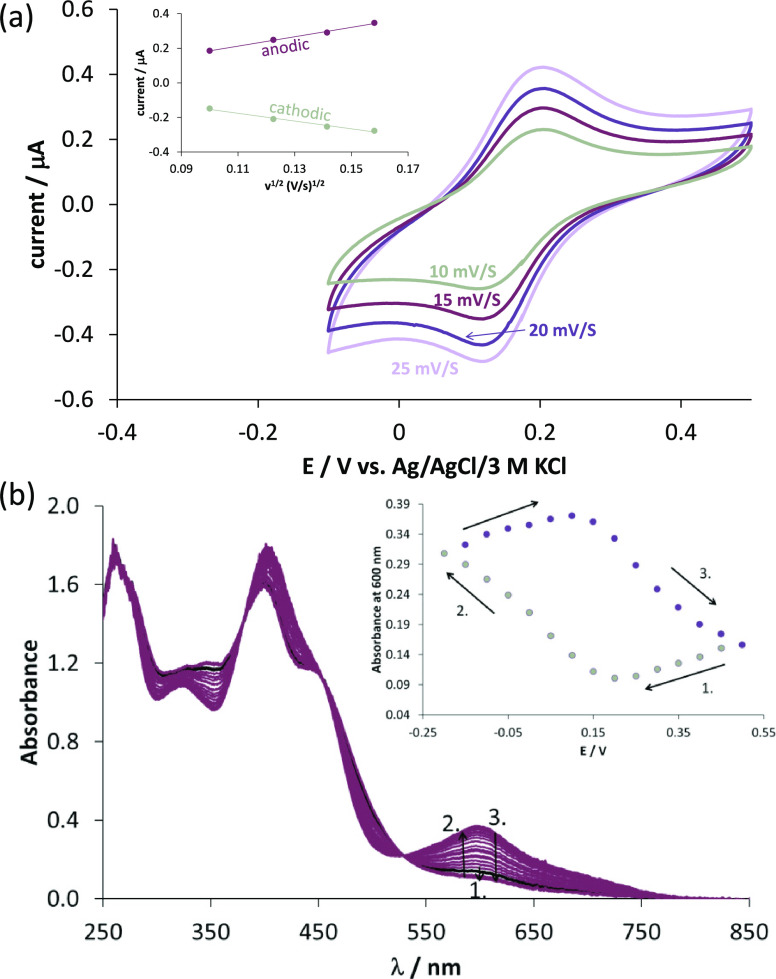
(a) Cyclic voltammograms of the iron complexes of VLX600 at different scan rates using the reference electrode Ag/AgCl/3 M KCl and Pt working and counter electrodes. The inserted figure shows the dependence of the peak current densities on the square root of the scan rate {*c*_VLX600_ = 1.0 mM, *c*_Fe(III)_ = 0.5 mM; 60% (v/v) DMF/buffered aqueous solution at pH 7.4; *t* = 25 °C; *I* = 0.1 M (tributylammonium nitrate (TBAN)); *l* = 1.70 mm}. (b) Changes in the UV–vis spectra of the iron complexes as a result of changing the potential using the spectroelectrochemical cell. The inserted figure exhibits the absorbance values measured at 600 nm as a function of potential. Numbers (1–3) near the spectra correspond to the potential ranges indicated in the inset {*c*_VLX600_ = 0.5 mM, *c*_Fe(III)_ = 0.25 mM; 90% (v/v) DMF/buffered aqueous solution at pH 7.4; *t* = 25 °C; *I* = 0.1 M (TBAN); *l* = 1.70 mm}.

Cyclic voltammetric (CV) measurements were performed to characterize the redox properties of the iron complex using different scan rates in the presence of 60% (v/v) dimethylformamide (DMF). The recorded voltammograms (Figure 8) and the electrochemical data (Table 3) show a one-electron transfer reversible process. The current is plotted against the square root of the applied scan rate (inset of Figure 8). The obtained curves are linear with similar slopes indicating a diffusion-controlled electrode reaction in both oxidation states. The formal potential calculated for the Fe(III)/Fe(II) redox couple is *E*_1/2_ = +384 ± 2 mV vs NHE, which also shows that VLX600 binds Fe(II) stronger than Fe(III) and suggests that these iron complexes are not able to undergo redox cycling in cells. To further investigate the electrochemical processes *in situ*, UV–vis spectroelectrochemical measurements were performed in addition to cyclic voltammetry, applying a special thin-layer cell with a microstructured honeycomb working electrode. An increase in absorbance was observed in the UV–vis spectra measured during cathodic reduction, whereas it was decreased during the anodic process. The reversibility of the process indicates that both iron complexes have similar coordination geometry and are stable without dissociation during the measurement.

**Table 3:** Electrochemical Data Collected for the Iron–VLX600 (1:2) System in 60% (v/v) DMF/Buffered Aqueous Solution at pH = 7.4 by Cyclic Voltammetric Measurements^a^

**scanrate/mV/s**	**10**	**15**	**20**	**25**
*E*_**c**_**/**mV	+131	+129	+131	+136
*E*_**a**_**/**mV	+194	+192	+192	+192
Δ*E***/**mV	63	63	60	56
*E*_**1/2**_**/**mV	+163	+161	+161	+164
*E*_**1/2**_**/**mV vs NHE	+385	+383	+383	+386
**|***i*_**c**_**/***i*_**a**_**|**	0.80	0.84	0.87	0.80

a *c*_VLX600_ = 1.0 mM, *c*_Fe(III)_ = 0.5 mM; *t* = 25 °C; *I* = 0.1 M (TBAN); reference electrode: Ag/AgCl/3 M KCl; working and counter electrodes: Pt.

### Complexation of VLX600 with Cu(II) Ions

**Figure 9:**
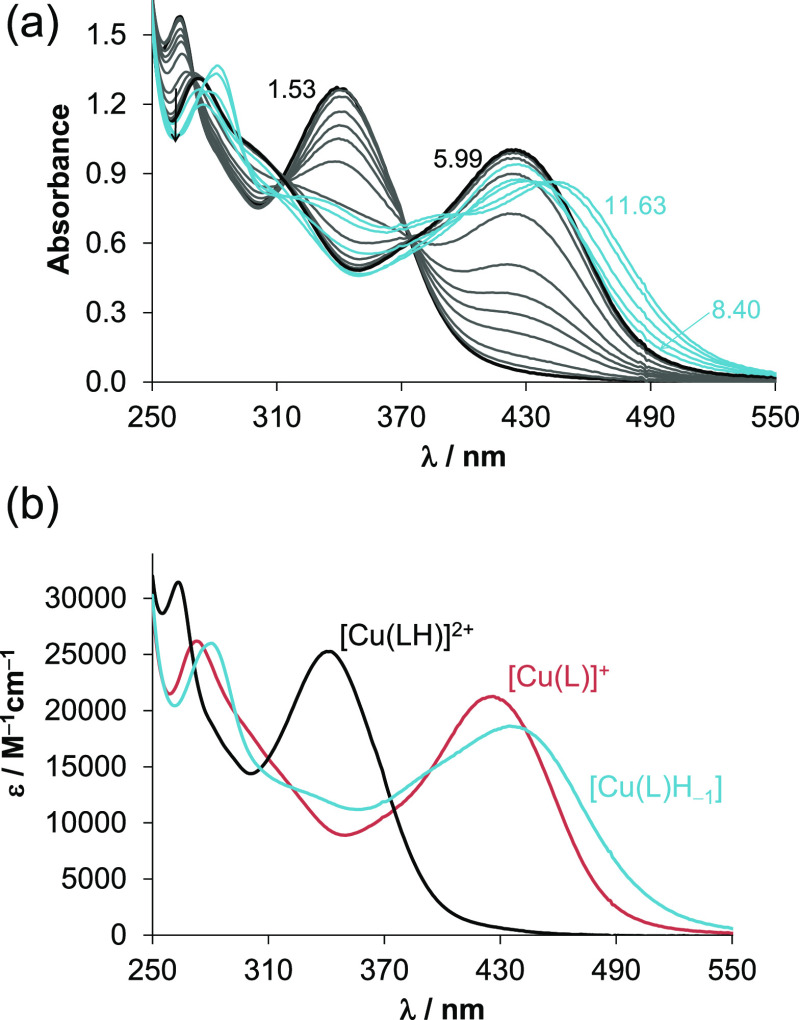
(a) UV–vis spectra of the Cu(II)–VLX600 (1:1) system recorded in the pH ranges between 1.5 and 11.7 in 30% (v/v) DMSO/H_2_O. (b) Molar absorbance spectra calculated for the various Cu(II) complexes {*c*_VLX600_ = 50 μM; *c*_Cu(II)_ = 50 μM; *I* = 0.10 M KCl; *l* = 1 cm; *t* = 25.0 °C}.

The *(N,N,N)-*donor set is also appropriate for efficient binding to essential divalent metal ions such as Cu(II) and Zn(II) other than Fe(II). Notably, the mechanisms of action of some iron-chelator α-*N*-heterocyclic TSCs such as Dp44mT or certain *N*,*N-*dimethyl derivatives of triapine have also been associated with the complexation with copper.^4,5^ The complex formation reaction of VLX600 with Cu(II) ions was investigated by UV–vis spectrophotometry in 30% (v/v) DMSO/H_2_O mixture, and to gain insight into the coordination environment around the metal ion, electron paramagnetic resonance (EPR) spectroscopy was also used. The measured absorbance values obtained by the UV–vis titrations (exemplary spectra shown in Figure 9a) were best fitted if the exclusive formation of mono-ligand complexes was assumed (Table 2). Including the formation of bis-ligand complexes into the calculations always resulted in a worse match between measured and calculated absorbance values, even when higher metal-to-ligand ratios were applied.

The computed molar absorbance spectra of [Cu(LH)]^2+^ and [Cu(L)]^+^ differ significantly (Figure 9b), most probably as a result of the deprotonation of the hydrazonic nitrogen (p*K*_a_ [Cu(LH)]^2+^ = 4.27), and [Cu(L)H_–1_] is assumed to be a mixed-hydroxido [Cu(L)(OH)] species formed from [Cu(L)]^+^ via the deprotonation of the coordinated aqua ligand (p*K*_a_ [Cu(L)]^+^ = 10.55). As a result, [Cu(L)]^+^ is the sole species at pH 7.4 based on the UV–vis data and the calculated concentration distribution curves (Figure S7).

**Figure 10:**
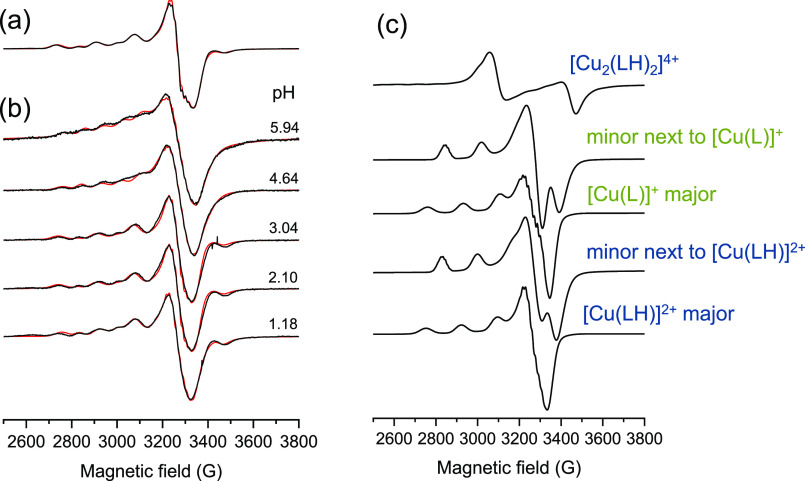
Frozen EPR spectra recorded for (a) the solid Cu(II) complex dissolved in DMSO and (b) the Cu(II)–VLX600 system in 30% (v/v) DMSO/H_2_O at various pH values (indicated in the figure). (c) Calculated component EPR spectra obtained by the simulation of the measured spectra (see EPR parameters in Table 4) {*c*_VLX600_ = 0.5 mM; *c*_Cu(II)_ = 0.33 mM; *I* = 0.10 M KCl; *T* = 77 K}.

To better understand the coordination modes of the Cu(II) complexes and to show if different binding modes are present, equimolar amounts of CuCl_2_ and VLX600 were dissolved in methanol, and the formed precipitate was collected for X-ray diffraction and EPR spectroscopic studies. The EPR spectrum of the solid Cu(II)–VLX600 complex was recorded after dissolution in DMSO (Figure 10a). For the Cu(II)–VLX600 system, anisotropic EPR spectra were also recorded at various pH values in 30% (v/v) DMSO/H_2_O (Figure 10b); however, spectra could be used for calculations only at pH < 6 due to the formation of precipitate at higher pH values. These spectra revealed a more complicated speciation, at least at 77 K. The obtained anisotropic EPR parameters are shown in Table 4. In the pH range between 1.2 and 3.1, in addition to the major complex that was identified as [Cu(LH)]^2+^, two minor species also appeared (Figure 10c).

**Table 4:** Anisotropic EPR Parameters of the Cu(II) Complexes of VLX600 Determined by the Simulation of Frozen Solution EPR Spectra Recorded at Various pH Values in 30% (v/v) DMSO/H_2_O (Figure 10a)^a^ {The Coupling Values Are in 10^–4^ cm^–1^ Unit (*I* = 0.1 M KCl; *T* = 77 K)}

	***g***_⊥_	***g*_∥_**	***A***_⊥_	***A*_∥_**	***a*^N^_0_**	***g*_0,cald._**^b^
			(×10^–4^ cm^–1^)	(×10^–4^ cm^–1^)	(×10^–4^ cm^–1^)	
[Cu(LH)]^2+^ major	2.064	2.241	12	174	16, 13, 13	2.123
minor species next to [Cu(LH)]^2+^	2.060	2.185	5	168		2.102
[Cu_2_(LH)_2_]^4+^^c^	2.056	2.247	10	178		2.120
[Cu(L)]^+^ major	2.060	2.233	19	175	16, 13, 13	2.117
minor species next to [Cu(L)]^+^	2.050	2.170	14	172		2.090

a The experimental errors were ±0.002 for *g*_⊥_, ±0.001 for *g*_∥_, ±2 × 10^–4^ cm^–1^ for *A*_⊥_, and ±1 × 10^–4^ cm^–1^ for *A*_∥_.

b Calculated by the equation *g*_0,cald._ = (2*g*_⊥_ + *g*_∥_)/3.

c The *g* and *A* values of [Cu(LH)]^2+^ were used for the simulation (dipolar coupling = 240 G, distance (Cu–Cu)_cald._ = 4.4 Å).

One of these minor species is the dimeric complex [Cu_2_(LH)_2_]^4+^, in which 4.4 Å distance of the Cu(II) ions can be calculated from the dipolar coupling values of 240 G by the point dipole approach. Its existence is feasible only in the frozen solution and possibly indicates a dimeric species that often appears in the case of planar aromatic ring ligands, where two complexes are arranged one above the other.^25^ The amount of the dimeric species is small, only 10% compared to the monomer complexes. The other minor species might be an isomeric form of [Cu(LH)]^2+^ or a bis-chelated complex. Similarly, two species were found in the pH range where the [Cu(L)]^+^ complex is formed (Figure 10c, major and minor species). Both minor species next to [Cu(LH)]^2+^ and [Cu(L)]^+^ have significantly lower *g*_∥_ tensors and *A*_⊥_ constants (Table 4) in comparison to their corresponding major counterparts, indicating a stronger ligand field and suggesting a different coordination mode. The EPR spectrum of the complex dissolved in DMSO (Figure 9a) also suggests the coexistence of the major and minor [Cu(LH)]^2+^ species. The ratio of major/minor isomer components is 80/20 for the protonated complex and 70/30 for the deprotonated complex.

**Figure 11:**
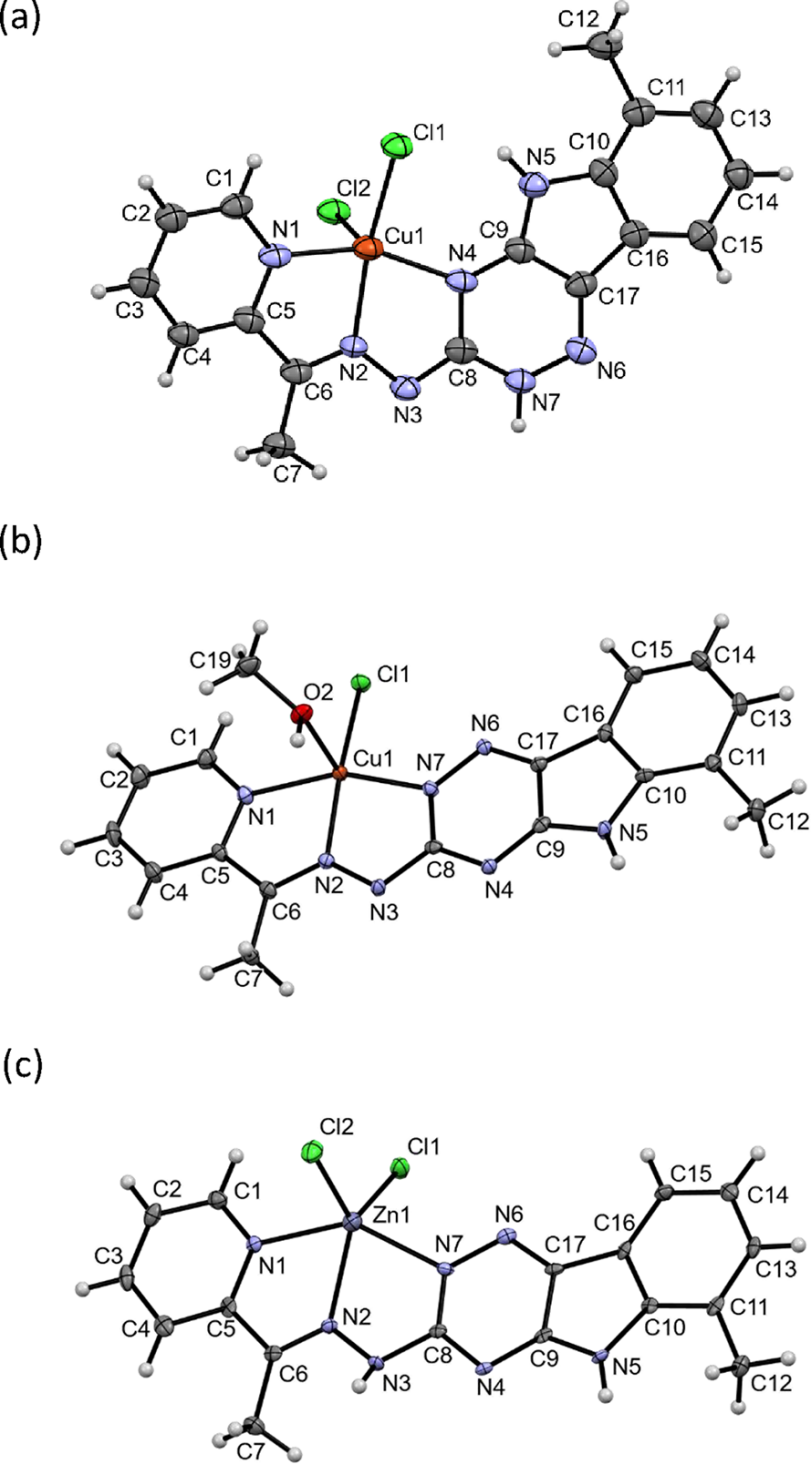
X-ray crystal structure of (a) [Cu(LH)Cl_2_], (b) [Cu(L)Cl(MeOH)], and (c) [Zn(LH)Cl_2_] (solvent molecules are omitted for clarity). (a) Selected bond lengths (Å) and bond and torsion angles (°) for [Cu(LH)Cl_2_]: Cu–N1 1.995(3), Cu–N2 1.961(3), Cu–N4 1.997(3), Cu–Cl1 2.2633(11), Cu–Cl2 2.5141(12), C6–N2 1.287(5), N2–N3 1.376(4), N3–C8 1.320(5), C8–N4 1.389(5), C8–N7 1.357(5), N7–N6 1.352(4) Å; ∠N1–Cu–N2 80.38(13), N2–Cu–N4 77.73(13), N1–Cu–N4 155.04(14), C5–C6–N2 113.5(3), C6–N2–N3 121.1(3), N2–N3–C8 108.2(3), N3–C8–N7 118.9(4), N3–C8–N4 121.8(4)°; ∠C5–C6–N2–N3 175.7(3), N2–N3–C8–N7 178.3(4), N2–N3–C8–N4 0.6(6)°. (b) Selected bond lengths (Å) and bond and torsion angles (°) for [Cu(L)Cl(MeOH)]: Cu–N1 2.0041(17), Cu–N2 1.9588(16), Cu–N7 1.9654(17), Cu–Cl1 2.2458(6), Cu–O2 2.2677(15), C6–N2 1.286(3), N2–N3 1.361(2), N3–C8 1.345(3), C8–N4 1.358(2), C8–N7 1.379(2), N7–N6 1.340(2) Å; ∠N1–Cu–N2 80.12(7), N2–Cu–N7 78.45(7), N1–Cu–N7 157.72(7), C5–C6–N2 113.45(18), ∠C6–N2–N3 122.66(17), ∠N2–N3–C8 110.29(16), N3–C8–N7 118.77(18), N3–C8–N4 116.73(17)°; ∠C5–C6–N2–N3 179.32, N2–N3–C8–N7 1.27, N2–N3–C8–N4 178.63°. (c) Selected bond lengths (Å) and bond and torsion angles (°) for [Zn(LH)Cl_2_]: Zn–N1 2.200(2), Zn–N2 2.133(2), Zn–N7 2.192(2), Zn–Cl1 2.2434(9), Zn–Cl2 2.2651(9), C6–N2 1.280(3), N2–N3 1.370(3), N3–C8 1.382(3), C8–N4 1.355(3), C8–N7 1.331(3), N7–N6 1.364(3) Å; ∠N1–Zn–N2 73.06(9), N2–Zn–N7 72.56(9), N1–Zn–N7 143.32(9), C5–C6–N2 113.6(3), C6–N2–N3 119.9(2), N2–N3–C8 114.7(2), N3–C8–N7 117.0(2), N3–C8–N4 114.2(3) °; ∠C5–C6–N2–N3 178.41, N2–N3–C8–N7 1.19, N2–N3–C8–N4 179.26°.

As the formation of the different species might be a consequence of linkage isomers, we attempted to obtain single crystals for the corresponding copper complexes to be analyzed by X-ray crystallography, and DFT calculations were also performed to examine the thermodynamic preference of the differently coordinated isomers. Therefore, single crystals were grown from the Cu(II)-VLX600 powder by recrystallization. Two different X-ray diffraction quality crystals could be obtained after slow diffusion of ethyl acetate or diethyl ether, into a methanolic solution, respectively (Figure 11a,b).

In the presence of diethyl ether, [Cu(LH)Cl_2_] crystals were formed in the triclinic *P*-1 space group (Figure 11a). In this case, the ligand is neutral with two chlorido ligands forming a square-pyramidal geometry with τ = 0.11 (trigonal-bipyramidal geometry τ = 1; square-pyramidal geometry τ = 0). However, the ligand is rotated via the N3–C8 bond, resulting in an N2/N7 *trans* configuration and coordination of the N^4^ of the 1,2,4-triazine (Figure 11b). In addition, the VLX600 ligand is present in an enamine tautomeric form with a protonated N7 instead of N3 (Scheme S1). This is in agreement with a shorter bond length of N3–C8 = 1.320(5) Å compared to 1.366(3) Å in the [Fe(II)(LH)_2_]^2+^ complex (Figure 6). A network of hydrogen bonds between the chlorido ligands is formed: intramolecular from Cl1 to N5 and intermolecular between Cl1 and N2 as well as Cl2 and N7 (Figure S8). Most probably, this is the major [CuLH]^2+^ species recognized by the EPR spectroscopic measurements (Table 4).

In the presence of ethyl acetate, [Cu(L)Cl(MeOH)] was formed in the monoclinic space group *P*21/*c* (Figure 11b). In this structure, the Cu(II) ion forms a nearly planar system with a deprotonated *(N,N,N)* tridentate VLX600 and one chlorido ligand. Methanol is coordinated at the fifth binding site of the complex with square-pyramidal geometry with τ = 0.20. Although the VLX600 ligand is deprotonated, the binding mode is comparable to the iron complex [Fe(II)(LH)_2_](NO_3_)_2_ with N2/N7 in *cis* configuration and VLX600 coordinated via pyridine, imine N, and the N^2^ of the 1,2,4-triazine (denoted as N7 in Figure 11a). Each two molecules form a pair via hydrogen bonds between the oxygen donor of methanol and the deprotonated hydrazone N (Figure S9). We suggest that the major [Cu(L)]^+^ species (Table 4) in the EPR spectra corresponds to this coordination mode.

As the Cu(II) complexes display the coordination of the ligand in different tautomeric forms, DFT calculations were also performed. In the first set of calculations, the geometry of [Cu(LH)Cl_2_] complexes was optimized, and the ligand coordinated to Cu(II) via the pyridine, imine N, and the N^2^ or N^4^ donor atoms of the 1,2,4-triazine moiety. Calculation of the relative energy between the two coordination isomers revealed that the N^4^ form is more stable than the N^2^ (Δ*G*_rel_ = 40.3 kJ/mol, see Table S8), which corroborates the results of the X-ray studies. Excellent agreement between the calculated and experimental structures was found (Table S8). The same calculation was performed for the [Cu(L)Cl(MeOH)]. In this case, the energy gap between the N^2^ and N^4^ isomers is relatively low (Δ*G*_rel_ = −2.5 kJ/mol); thus, the crystallization of the tautomeric form from the solution is subject to random selection.

The significant disparity in EPR parameters obtained for the minor and major [CuLH]^2+^ and [Cu(L)]^+^ species (as shown in Table 4) cannot be attributed to the variations in the coordination mode of the ligands in different tautomeric forms. Therefore, the EPR parameters for the Cu(II) complexes were also calculated. In these calculations, the chlorido ligands were replaced with water molecules, yielding the [Cu(LH)(H_2_O)_2_]^2+^ and the [Cu(L)(H_2_O)_2_]^+^ complexes. Calculations of relative energies estimated that [Cu(LH)(H_2_O)_2_]^2+^ favors the pyridine, imine-N, and N^4^ coordination mode, whereas the pyridine, imine-N, and N^2^ donor set becomes favorable for [Cu(L)(H_2_O)_2_]^+^ complex. The calculated EPR parameters ([Cu(LH)(H_2_O)_2_]^2+^: *A*_∥_ = 528 MHz (N^4^); 554 MHz (N^2^) vs 520 MHz (experimental); [Cu(L)(H_2_O)_2_]^+^*A*_∥_ = 546 MHz (N^4^ and N^2^) vs 525 MHz (experimental)) are in good agreement with the experimental values, confirming the structures of the complexes in solution; however, two important pieces of information can be gained from the results (Cartesian coordinates of the complexes are summarized in the SI, Tables S9–S16). The calculated EPR parameters of the coordination isomers (N^2^ or N^4^ species) are very similar (Table S17) and cannot be distinguished by using EPR. It is suggested that the minor species possessing significantly lower *g*_∥_tensors and *A*_⊥_ constants should have a different coordination mode in comparison to the major species. For these minor species, two scenarios are plausible. Because the EPR studies were performed in the excess of ligand (beside the equimolar ratio) and in the DMSO/H_2_O solvent mixture, the formation of bis-chelated species or the binding of the DMSO solvent may provide new Cu(II) complexes. Thus, the geometry of these potential species ([Cu(HL)_2_]^2+^, [Cu(HL)DMSO(Cl)]^+^, and [Cu(HL)(DMSO)_2_]^2+^) was optimized, and their EPR parameters were calculated (Tables S18–S21). EPR parameters of [Cu(HL)_2_]^2+^and [Cu(HL)(DMSO)_2_]^2+^ predicted by DFT are very similar (*g*_∥_ = 2.165, *A*_∥_ = 498 MHz for [Cu(HL)_2_]^2+^ and *g*_∥_ = 2.174, *A*_∥_ = 522 MHz for [Cu(HL)(DMSO)_2_]^2+^, respectively); hence, it is not trivial to distinguish these species. However, the formation of [Cu(HL)(DMSO)_2_]^2+^ is unlikely on the basis of the calculated parameters. Moreover, it is obvious that the presence of a mixed-ligand [Cu(HL)DMSO(Cl)]^+^ complex can be ruled out. In one experiment, the DMSO solvent was replaced with DMF as a non-coordinating solvent, and the EPR spectra were recorded (Figure S10). The results (Table S22) clearly showed the formation of the same minor species, leading to the conclusion that Cu(II) is capable of binding two ligands when VLX600 is applied in excess under the condition used for the EPR measurements (77 K).

The redox properties of the copper complexes of VLX600 were monitored by CV measurements (Figure S11, Table 5), and the obtained electrochemical data indicate irreversible processes as the anodic current was always much lower than the cathodic one, and a large peak separation was detected. The irreversible feature might be due to the inappropriate coordination geometry of the tridentate VLX600 for Cu(I), leading to the dissociation of the complex in the lower oxidation state. The relatively low redox formal potential (*E*_1/2_ = −121 ± 8 mV vs NHE) also suggests the stronger preference of the ligand for Cu(II) over Cu(I).

**Table 5:** Electrochemical Data Collected for the Copper–VLX600 (1:1) System in 60% (v/v) DMF/Buffered Aqueous Solution at pH 7.4 by Cyclic Voltammetric Measurements^a^

**scanrate/mV/s**	**5**	**10**	**15**	**20**
*E*_**c**_**/**mV	–408	–415	–425	–437
*E*_**a**_**/**mV	–259	–266	–269	–266
Δ*E***/**mV	149	149	156	171
*E*_**1/2**_**/**mV	–333	–341	–347	–352
*E*_**1/2**_**/**mV vs NHE	–111	–119	–125	–130
**|***i*_**c**_**/***i*_**a**_**|**	2.82	2.17	1.88	1.86

a *c*_VLX600_ = 1.0 mM, *c*_Cu(II)_ = 1.0 mM; *t* = 25 °C; *I* = 0.1 M (TBAN); reference electrode: Ag/AgCl/3 M KCl; working and counter electrodes: Pt.

### Complexation of VLX600 with Zn(II) Ions

**Figure 12:**
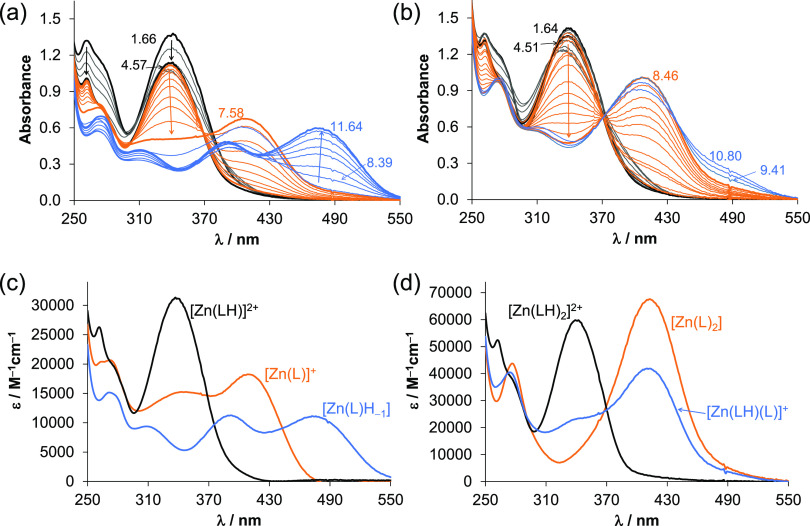
UV–vis spectra of the Zn(II)–VLX600 chemical system recorded in the pH ranges between 1.5 and 11.7 in 30% (v/v) DMSO/H_2_O at (a) 1:1 and (b) 1:2 metal-to-ligand ratio. Molar absorbance spectra calculated for the (c) monoligand and the (d) bis-ligand complexes {*c*_VLX600_ = 51 M; *c*_Zn(II)_ = 50 or 25 μM; *I* = 0.10 M KCl; *l* = 1 cm; *t* = 25.0 °C}.

UV–vis titrations of the Zn(II)–VLX600 system (Figure 12a,b) revealed the formation of mono and bis complexes (Table 2), and the individual molar absorbance spectra of the Zn(II) complexes are shown in Figure 12c,d. In the protonated complexes ([Zn(LH)]^2+^ and [Zn(LH)_2_]^2+^) as well as in the complexes containing the monoanionic ligand ([Zn(L)]^+^, [Zn(LH)(L)]^+^, and [Zn(L)_2_]), similar coordination modes are likely, exactly as it was found for the corresponding Fe(II) complexes. Interestingly, the complex [Zn(L)H_–1_] (= [Zn(L)(OH)]) has a significantly different molar absorbance spectrum compared to that of [Zn(L)]^+^. The difference is much larger than what we would expect from the deprotonation of a coordinated aqua ligand (Figure 12c).

For a better insight into the coordination modes of the Zn(II) complexes of VLX600, X-ray diffraction quality crystals of [Zn(LH)Cl_2_] were obtained from a methanolic solution (with 1% (v/v) DMSO) and slow diffusion of EtOAc (Figure 11c). In this complex, VLX600 is bound in the “typical” imine tautomeric form with the protonated hydrazone NH comparable to the structure of [Fe(II)(LH)_2_](NO_3_)_2_ (Figure 6). Also N2/N7 is in *cis* configuration, and VLX600 coordinated via pyridine, imine N, and the N^2^ of the 1,2,4-triazine. Although the Zn atom is distinctly out of the square plane of N1–N2–N7-Cl1, the geometry is again square-pyramidal with τ = 0.16. No intra- or intermolecular hydrogen bonds are formed between the [Zn(LH)Cl_2_] molecules, only to two molecules of co-crystallized DMSO. In contrast to [Fe(II)(LH)_2_](NO_3_)_2_ (Figure 6), the hydrogen at N3 is 0.509 Å out of the N2, N3, and C8 plane of the VLX600 ligand due to a strong hydrogen bond to the O of one DMSO molecule. This is also indicated by the elongated bond lengths of N2–N3 = 1.370(3) and N3–C8 = 1.382(3) compared to the respective iron complex [Fe(II)(LH)_2_](NO_3_)_2_ (Figure 5) at N2–N3 = 1.366(2) and N3–C8 = 1.366(3) where this hydrogen is almost in plane.

Because the formation of coordination isomers (N^2^ vs N^4^ coordination modes) is also plausible in this system, the geometry of the [Zn(LH)Cl_2_] and [Zn(LH)(H_2_O)_2_]^2+^ complexes was optimized by DFT, and the relative energies of the isomers were calculated. The structures of the complexes as well as their selected bond lengths, angles, and Cartesian coordinates are summarized in the SI (Figures S12 and S13 and Tables S23–27). DFT calculations predicted a low energy gap between the coordination isomers; therefore, we expect the simultaneous existence of N^2^ or N^4^ coordinated Zn(II) complexes. It is noteworthy that the replacement of chlorido ligand with coordinated water molecule decreases the bond length formed between Zn(II) and coordinated nitrogen atoms (Table S23), yielding a more relaxed structure.

### Comparison of the Stability of the VLX600 Complexes Formed with the Different Metal Ions

**Figure 13:**
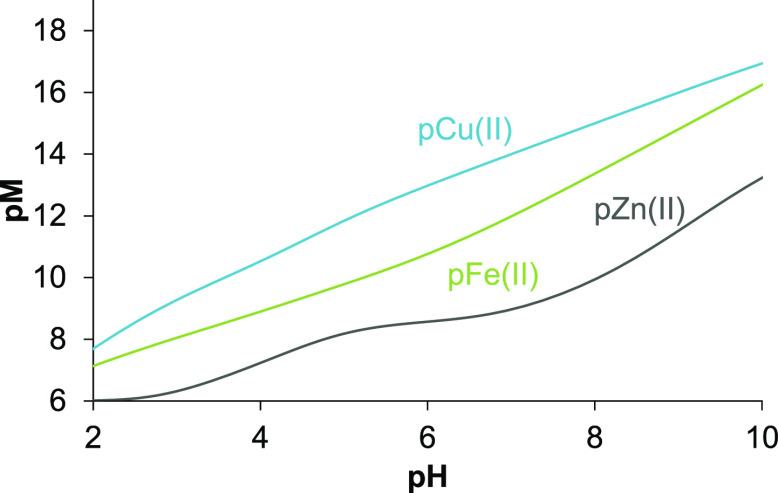
The pM values computed on the basis of the determined overall stability constants for the Cu(II), Fe(II), and Zn(II) complexes of VLX600 using 1 μM metal ion and 10 μM ligand concentrations {30% (v/v) DMSO/H_2_O; *I* = 0.10 M KCl; *t* = 25.0 °C}.

By comparing the overall stability constants of the complexes formed with the studied biologically essential metal ions (Table 2), it can be concluded that the Cu(II) complexes have the highest formation constants, whereas Fe(II) and Zn(II) complexes display lower stability. As different types of complexes are formed, it is difficult to ascertain the metal ion preferences of VLX600 based on these formation constants. Therefore, pM values (= –log[M]) were computed at various pH values for adequate comparison (Figure 13). The higher the pM value is, the stronger is the binding ability to the metal ion. The calculation clearly shows the following trend for the metal ion binding: Cu(II) > Fe(II) > Zn(II). However, although VLX600 binds Cu(II) more effectively than Fe(II) in our artificial settings, intracellularly, where copper is predominantly found in its reduced Cu(I) state, a preferred interaction with Fe(II) is very likely.

### *InVitro* Anticancer Properties of VLX600 Alone and in the Presence of Essential Metal Ions

The *in vitro* cytotoxic activity of VLX600 was determined in six different human cancer cell lines (A549, CH1/PA-1, SW480, Colo205, Colo320, and MCF-7), as well as in a nontumoral human lung fibroblast cell line (MRC-5). The colorimetric 3-(4,5-dimethylthiazol-2-yl)-2,5-diphenyl-tetrazolium bromide (MTT) assay was performed to demonstrate the cytotoxic effects of VLX600 alone or after the addition of different metal salts, namely, in combination with the chloride salts of the three metal ions Cu(II), Zn(II), and Fe(III). The obtained results are summarized in Table 6. VLX600 displayed a strong cytotoxic effect in the submicromolar range (IC_50_ values between 0.039 and 0.51 μM upon exposure times of 72 or 96 h) in all treated cancer cell lines. The strongest antiproliferative activity was found in ovarian teratocarcinoma cells (CH1/PA-1), whereas the detected IC_50_ values in all other cell lines range about 1 order of magnitude higher (which may partially be due to the shorter exposure time). Somewhat higher IC_50_ values were reported for this compound in monolayer colon cancer cell cultures (IC_50_ = 1.4–3.7 μM) by other authors, but it was found to be more potent than other iron chelators such as triapine or desferoxamine B.^14^ Based on our studies, VLX600 exhibits a cytotoxic effect in nontumoral MRC-5 cells with an IC_50_ value 0.60 ± 0.01 μM, suggesting a weak selectivity for cancer cells compared to normal cells.

**Table 6:** *In Vitro* Cytotoxicity in A549, CH1/PA-1, SW480, Colo205, Colo320, and MCF-7 Cancer Cell Lines as well as MRC-5 Fibroblasts, Expressed as IC_50_ Values (in μM) of VLX600 Alone or in the Presence of 1 equiv CuCl_2_, 0.5 equiv ZnCl_2_, and 0.5 equiv FeCl_3_

**IC**_**50**_**/μM**	**A549**	**CH1/PA-1**	**SW480**	**Colo205**	**Colo320**	**MCF-7**	**MRC-5**
cell number	3 × 10^3^	10^3^	2 × 10^3^	10^4^	10^4^	10^4^	10^4^
exposure time	96 h	96 h	96 h	72 h	72 h	72 h	72 h
VLX600	0.16 ± 0.03	0.039 ± 0.003	0.16 ± 0.01	0.20 ± 0.09	0.14 ± 0.01	0.51 ± 0.09	0.60 ± 0.01
VLX600 + 1 equiv CuCl_2_	0.16 ± 0.02	0.037 ± 0.012	0.37 ± 0.08	0.24 ± 0.03	0.16 ± 0.01	0.41 ± 0.01	0.49 ± 0.03
VLX600 + 0.5 equiv ZnCl_2_	0.16 ± 0.03	0.029 ± 0.005	0.19 ± 0.03	0.12 ± 0.02	0.18 ± 0.01	1.1 ± 0.1	0.71 ± 0.03
VLX600 + 0.5 equiv FeCl_3_	0.27 ± 0.07	0.054 ± 0.012	0.27 ± 0.04	0.33 ± 0.04	0.25 ± 0.01	1.93 ± 0.09	0.49 ± 0.01
CuCl_2_	142 ± 16	28 ± 5	154 ± 21	>10	>10	>10	>10
ZnCl_2_	123 ± 9	66 ± 3	125 ± 16	>10	>10	>10	>10
FeCl_3_	>200	>200	>200	>10	>10	>10	>10

Based on our solution speciation data, Cu(II), Zn(II), and Fe(III) form positively charged complexes with VLX600 at pH 7.4 (100% [Cu(L)]^+^; 52% [Zn(LH)(L)]^+^, 19% [Zn(LH)_2_]^2+^, 19% [Zn(L)]^+^; most likely ∼100% [Fe(III)(L)_2_]^+^, which is slowly reduced), which may result in limited cellular uptake, possibly affecting anticancer activity. Thus, IC_50_ values were also determined after premixing of VLX600 with the different metal salts (FeCl_3_, CuCl_2_, ZnCl_2_). It was found that the IC_50_ values are in a similar range as for VLX600 alone with mostly minor deviations in either direction depending on the cell line. These data are in contrast to the results reported for HCT116 cells where the antiproliferative activity of VLX600 was abrogated by addition of FeCl_2_ and FeCl_3_.^14^ Our data suggest that the tested metal ions do not synergize with VLX600 in terms of cytotoxicity and that the premix complex preparation with Cu(II), Zn(II), or Fe(III) is not recommended as a strategy to obtain more effective antiproliferative activities. In addition, we can assume that if VLX600 forms complexes with Cu(II), Zn(II), and Fe(III) in the extracellular space (where the metal ions are mostly found in their higher oxidation states), it does not improve the membrane transport via passive diffusion. Notably, the Fe(II) bis complex of VLX600 is neutral ([Fe(II)(L)_2_]), but its formation is more likely to happen in the intracellular space. The cytotoxic potencies of the metal salts alone are mostly negligible or at least 2–3 orders of magnitude lower than that of VLX600.

The ability of VLX600 to induce ROS production was investigated by means of the DCFH-DA assay in CH1/PA-1 and SW480 cells. It has previously been reported by other authors that VLX600 alone does not cause ROS production.^13,14,16^ Interestingly, also in the presence of Cu(II), Zn(II), or Fe(III) ions, no ROS formation could be observed, like for VLX600 alone (Figure S14).

**Figure 14:**
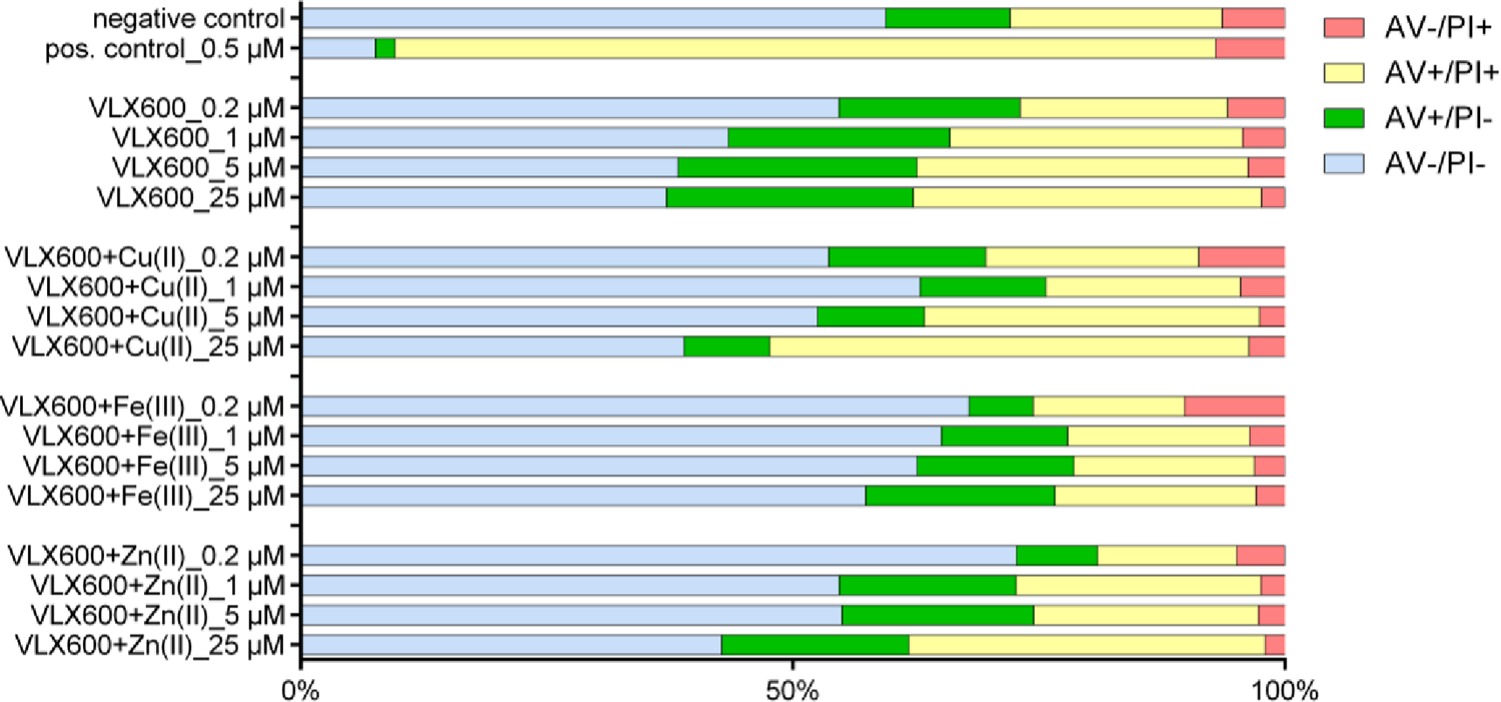
Apoptosis/necrosis induction by four different concentrations of VLX600 alone or in the presence of 1 equiv CuCl_2_, 0.5 equiv ZnCl_2_, and 0.5 equiv FeCl_3_. Viable (AV−/PI−), early apoptotic (AV+/PI−), late apoptotic (AV+/PI+), and necrotic (AV–/PI+) cell counts were detected by flow cytometry of CH1/PA-1 cells double-stained with annexin V-FITC and PI (positive control: 1-P^26^). Note the rather high baseline incidence of apoptosis in untreated CH1/PA-1 cells (negative control).

Induction of apoptosis by VLX600 in the absence or presence of 1 equiv CuCl_2_, 0.5 equiv ZnCl_2_, and 0.5 equiv FeCl_3_ was investigated in CH1/PA-1 cells (the most chemosensitive cell line). Cells were double stained with annexin V-FITC and propidium iodide (PI) and analyzed by flow cytometry. Subpopulations of necrotic cells (AV–/PI+), late apoptotic cells (AV+/PI+), early apoptotic cells (AV+/PI−), and viable cells (AV–/PI−) are represented in Figure 14 as percentages of total events, illustrating that VLX600 induces early and late apoptosis in a concentration-dependent manner. However, the sums of early and late apoptotic events were rather decreased when the metal ions were added, especially in the case of Fe(III).

## Conclusions

The cellular interaction of potential drugs with iron ions as well as modulation of iron-dependent processes is a viable approach in the fight against cancer. This is particularly relevant due to the increased iron uptake and dependence in cancer cells. VLX600 is an iron chelator, which interferes with the iron metabolism intracellularly, leading to the inhibition of mitochondrial respiration and finally to cell death. Despite the clinical investigation of VLX600, its solution chemical properties and metal complexation have not been extensively explored in detail until now. Herein, we investigated the solubility, membrane permeability, and proton dissociation processes of VLX600. The protonated form of VLX600 has four dissociable groups, namely, the pyridinium nitrogen (NH^+^), N^2^H^+^ of the 1,2,4-triazine moiety, and the hydrazone (NH) and indole (NH) nitrogen. However, p*K*_a_ values could be obtained only for the first three functional groups. It was found that the neutral HL form is predominant at pH 7.4. The distribution coefficients of VLX600 at pH 7.4 revealed high lipophilicity and cell membrane permeability. The X-ray structure of VLX600 in its neutral form was also reported.

The interaction of this iron chelator with Fe(III), Fe(II), Cu(II), and Zn(II) ions was mostly studied using UV–vis spectrophotometry. In the case of Fe(III), the recorded spectra indicated complex formation; however, changes observed during the measurement suggested the occurrence of a redox reaction, resulting in the production of the Fe(II) complex. Based on the UV–vis spectra of the Fe(II)–VLX600 system at various pH values and metal-to-ligand ratios, formation constants for mono- and bis-ligand complexes were determined in different protonation states. It can be concluded that the neutral [Fe(II)(L)_2_] bis complex is the predominant species at physiological pH. Its single-crystal structure indicates that VLX600 forms an octahedral coordination system with Fe(II), in which the ligand is bound to the metal ion in a tridentate mode with two nitrate ions neutralizing the charge of the central ion. Nitrogens of pyridine, hydrazone, and the N^2^ of the 1,2,4-triazine moiety are coordinated. The XPS measurements also confirmed the formation of a low-spin Fe(II) complex. Cyclic voltammetric measurements revealed a relatively high formal potential value for the Fe(III)/Fe(II) redox couple, indicating that VLX600 binds Fe(II) stronger than Fe(III).

For the Cu(II) ions, the formation of mono-ligand complexes in different protonation states was observed based on the UV–vis titration data. This was confirmed by the EPR spectroscopic measurements. However, minor amount of bis-ligand complex could also be detected at ligand excess in the frozen samples. DFT calculations suggested that the [Cu(LH)]^2+^ complex favors the pyridine, hydrazone, and N^4^ coordination mode, whereas the deprotonation of the complex supports the stabilization of the N^2^ coordination isomer. Single crystals of [Cu(L)Cl(MeOH)] and [Cu(LH)Cl_2_] revealed the tridentate (*N*,*N*,*N*) motif of the ligand via the pyridine, hydrazone and N^2^ and N^4^ of the 1,2,4-triazine moiety for [Cu(L)Cl(MeOH)] and [Cu(LH)Cl_2_], respectively. Based on the cyclic voltammetric measurements, the relatively low redox formal potential also suggests a stronger preference for Cu(II) in favor of Cu(I).

With Zn(II) ions, VLX600 forms mono and bis complexes, and in the [Zn(LH)Cl_2_] complex, the neutral VLX600 coordinates via the pyridine, hydrazone, and N^2^ of the 1,2,4-triazine moiety in a distorted trigonal-bipyramidal geometry.

The *in vitro* cytotoxic activity of VLX600 was determined in six different types of human cancer cell lines (A549, CH1/PA-1, SW480, Colo205, Colo320, and MCF-7) and in a nontumoral human lung fibroblast (MRC-5) cell line. The MTT tests were conducted without and with addition of Cu(II), Zn(II), and Fe(III) salts. VLX600 displayed strong cytotoxic effects, with the highest activity in teratocarcinoma CH1/PA-1 cells. In the presence of metal ions, only minor changes in IC_50_ values could be observed. By flow cytometry, concentration-dependent apoptosis induction could be confirmed for VLX600, but the percentage of apoptotic events was rather decreased in the presence of Cu(II), Zn(II), and Fe(III) salts. No evidence for the generation of reactive oxygen species by VLX600 or any of its *in situ* formed metal complexes was found.

Taken together, the here presented data show that not only iron can be strongly coordinated by VLX600 but also other physiologically relevant metal ions. Consequently, future biological studies should consider these competitors when investigating the activity and mode of action of VLX600.

## Experimental Section

### Chemicals

TBAN and HEPES were purchased from Sigma-Aldrich in puriss quality. HCl, KOH, KCl, DMSO, DMF, and KH-phthalate were obtained from Molar Chemicals (Hungary) and used without further purification. The stock solutions of metal ions were prepared by the dissolution of CuCl_2_, FeCl_3_, and ZnCl_2_ in water, and the concentration was determined by complexometry using ethylenediaminetetraacetic acid (EDTA). The Fe(II) stock solution was generated through the interaction of iron powder and HCl in a purified, strictly oxygen-free argon atmosphere. Subsequently, the solution was filtered and stored for use under anaerobic conditions in a laboratory glovebox (GP(Campus), Jacomex), ensuring that the O_2_ level was kept below 1 ppm. The concentration of the Fe(II) solution was determined through titrations with KMnO_4_. All solutions were prepared using Milli-Q. If required, the pH was adjusted to the desired level by adding of HCl or KOH. All solvents used for the syntheses were dried and purified according to standard procedures.

### Synthesis Procedure and Characterization of VLX600

For the characterization, one-dimensional NMR spectra were recorded at 25 °C using a Bruker FT-NMR spectrometer AV NEO 500 at 500.10 MHz. The final compound was characterized with one- and two-dimensional NMR using a Bruker FT-NMR AVIII 600 MHz spectrometer at 600.25 MHz (^1^H) and 150.93 MHz (^13^C), respectively. All spectra were measured in DMSO-*d*_6_. Chemical shifts (ppm) were referenced internally to the residual solvent peaks. For the description of the spin multiplicities, the following abbreviations were used: s = singlet, bs = broad singlet, d = doublet, t = triplet, td = triplet of doublets, and m = multiplet. High-resolution mass spectra were measured on a Bruker maXis UHR ESI time-of-flight mass spectrometer. Elemental analysis measurements were performed on a Eurovector EA 3000 CHNS-O Elemental Analyzer at the Microanalytical Laboratory of the University of Vienna.

#### Synthesis of 3-Hydrazineyl-6-methyl-5*H*-[1,2,4]triazino[5,6-b]indole

Commercially available 6-methyl-4,5-dihydro-3*H*-[1,2,4]triazino[5,6-*b*]indole-3-thione (1.00 g, 4.62 mmol, 1 equiv) was suspended in hydrazine monohydrate (12.5 mL, 99.88 mmol, 54 equiv). The mixture was stirred under reflux for 20 h. A light yellow solid formed, which was filtered off, washed with water and EtOH, and dried *in vacuo* at 40 °C overnight. Yield: 947.6 mg (96%). ^1^H NMR (500 MHz, DMSO-*d*_6_): δ = 11.88 (bs, 1H), 8.55 (s, 1H), 7.93 (d, *J* = 7.4 Hz, 1H), 7.30 (d, *J* = 7.3 Hz, 1H), 7.20 (t, *J* = 7.5 Hz, 1H), 4.32 (s, 2H), 2.48 (s, 3H).

#### Synthesis of VLX600

3-Hydrazineyl-6-methyl-5*H*-[1,2,4]triazino[5,6-*b*]indole (300 mg, 1.40 mmol, 1 equiv) was suspended in water (3.0 mL) and EtOH (4.5 mL), and 2-acetylpyridine (0.785 mL, 7.00 mmol, 5 equiv) was added. The mixture was stirred under reflux for 5 h. A light yellow solid formed, which was filtered off, washed with a mixture of 40% EtOH in water, and dried *in vacuo* at 40 °C overnight. Yield: 437.1 mg (98%). Elemental analysis, calcd for C_17_H_15_N_7_·1.5H_2_O (%): C, 59.29; H, 5.27; N, 28.47. Found (%): C, 59.28; H, 4.95; N, 28.35. ESI-TOF in MeCN/MeOH + 1% H_2_O: *m*/*z* (M + H, calcd) = 318.1462 *m*/*z*, (M + H, found) = 318.1460 *m*/*z*. ^1^H NMR (600 MHz, DMSO-*d*_6_, Figure S15) E-isomer (∼90%): δ = 12.43 (s, 1H, H11), 10.89 (s, 1H, H8), 8.59 (d, *J* = 4.2 Hz, 1H, H1), 8.16 (d, *J* = 8.1 Hz, 1H, H4), 8.05 (d, *J* = 7.6 Hz, 1H, H17), 7.87 (td, *J* = 7.9, 1.7 Hz, 1H, H3), 7.40–7.35 (m, 2H, H2, H15), 7.27 (t, *J* = 7.5 Hz, 1H, H16), 2.52 (s, 3H, H14), 2.49 (3H, H7; below DMSO, observed in 2D only). Z-isomer (∼10%): δ = 14.88 (s, 1H), 12.43 (s, 1H, overlap with E-isomer peak), 8.88 (d, *J* = 3.9 Hz, 1H), 8.09 (td, *J* = 7.9, 1.8 Hz, 1H), 8.02 (d, *J* = 7.8 Hz, 1H), 7.79 (d, *J* = 8.1 Hz, 1H), 7.57 (dd, *J* = 7.1, 5.2 Hz, 1H), 7.41–7.35 (m, 1H, overlap with E-isomer peak), 7.29–7.24 (m, 1H, overlap with E-isomer peak), 2.49 (3H, below DMSO, observed in 2D only), 2.45 (s, 3H). ^13^C NMR (151 MHz, DMSO-*d*_6_, Figure S16) E-isomer: δ = 159.00 (C9), 155.80 (C5), 148.65 (C10), 148.53 (C1), 148.22 (C6), 138.99 (C19), 138.93 (C12), 136.26 (C3), 129.82 (C15), 123.26 (C2), 122.02 (C16), 121.83 (C13), 119.83 (C4), 118.33 (C18), 117.70 (C17), 16.55 (C14), 11.94 (C7).

### X-ray Crystallography

Single-crystal X-ray diffraction data were collected with a Stadivari Diffractometer (STOE & Cie GmbH, Germany) equipped with an EIGER2 R500 detector (Dectris Ltd., Switzerland). Data were processed and scaled with the STOE software suite X-Area (STOE & Cie GmbH). Structures were solved with SHELXT^27^ and refined with SHELXL^28^ or Olex2.^29^ Model building was done with Olex2^29^ or ShelXle.^30^ Structures were validated with CHECKCIF (https://checkcif.iucr.org/). See the respective CIF files for EPRexact versions and more details. Experimental data and CCDC codes (available online: http://www.ccdc.cam.ac.uk) can be found in Table S2.

### Lipophilicity and Parallel Artificial Membrane Permeability Assays (PAMPA)

The distribution coefficient (*D*_7.40_) of VLX600 was determined at pH 7.40 using the shake-flask method in a system comprising *n*-octanol and buffered aqueous solution (0.10 M KCl, at 25.0 ± 0.2 °C). It was dissolved at 50 μM concentration in *n*-octanol presaturated with 20 mM phosphate buffer. The solution was mixed with aqueous buffer using a volumetric ratio of 1:10 and subjected to 360° vertical rotation for 3 h. After mixing, the samples were centrifuged at 5000 rpm for 5 min. Two phases were separated, and their UV–vis spectra were recorded. The *D*_7.40_ value was calculated as follows:
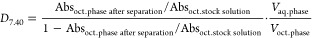


PAMPA was applied for VLX600 with a Corning Gentest precoated PAMPA Plate System.^31^ The initial ligand stock solution was prepared in DMSO (1 mM). A 25 mM HEPES solution with 0.1 M KCl was utilized as both the donor and acceptor buffer solutions. The donor plates were prepared by mixing the ligand with a solution containing the buffer and KCl. The donor plate was loaded with 300 μL of the donor solutions, whereas the acceptor plate was filled with 200 μL of the buffer. The samples were incubated at 25 °C for 5 h. After the incubation time, the donor and the acceptor phases were transferred to Eppendorf tubes, and their UV–vis spectra were recorded. *P*_eff_ values were calculated according to the equation reported by Yu et al.^32^

### UV–Vis Spectrophotometric Titrations

UV–vis spectra in the range of 200–800 nm were recorded using an Agilent Carry 8454 diode array spectrophotometer, and the path length for the measurements was set at 1 cm. The spectra were recorded for the Fe(II)-containing samples using an Avantes AvaSpec-ULS2048CL-EVO spectrometer with an AvaLight-DHc light source and FDP-7UVIR200-2-VAR transmission dip probe. The titrations were carried out with carbonate-free KOH solutions, and their accurate (∼0.10 M) concentrations were determined by pH-potentiometric titrations. Spectrophotometric titrations were performed in a solvent mixture of 30% (v/v) DMSO/H_2_O on samples containing the ligand at 50 μM concentration, and the metal ions-to-ligand ratios were 1:1, 1:2, and 1:3. The titrations were performed by a KOH solution in the presence of 0.1 M KCl at 25.0 °C in the pH range from 1.5 to 12.5. A Metrohm 665 Dosimat buret and an Orion 710A pH-meter equipped with a Metrohm combined electrode (type 6.0234.100) were used for the pH titrations. During the titrations, Ar gas was passed over the solutions, whereas the Fe(II)-containing samples were titrated in a laboratory glovebox (GP(Campus), Jacomex, O_2_ content less than 1 ppm). The electrode system was calibrated by the Irving method^33^ when the pH = −log[H^+^] scale was obtained from blank titrations’ data (HCl vs KOH). The average water ionization constant (p*K*_w_) was 14.53 ± 0.05. Proton dissociation constants (p*K*_*a*_) of the ligand, the overall stability (formation) constants of the complexes, and the individual UV–vis spectra of the species in the different protonation states were calculated by the computer program PSEQUAD.^34^ Hydrolysis constants of the Fe(II) species were included in the speciation models (log β for Fe(II) species:^35^ [FeH_–1_]^+^: −9.43, [FeH_–2_]: −20.73, [FeH_–3_]^−^: −32.68.

### Electrochemical and Spectroelectrochemical Studies

A conventional three-electrode system was used to record the voltammograms under an argon atmosphere with an Autolab PGSTAT 204 potentiostat/galvanostat monitored by Metrohm’s Nova software. The measurements were performed for the Fe(III)–ligand (1:2) and Cu(II)–ligand (1:1) systems at 25.0 ± 0.1 °C at pH 7.4 (10 mM HEPES) using 0.1 M TBAN as the supporting electrolyte. The concentration of the metal ions was 0.5 mM. Argon was also passed over the solutions before recording the cyclic voltammograms. A platinum electrode was used as the working and auxiliary electrode, and Ag/AgCl/3 M KCl was used as the reference electrode. The electrochemical system was calibrated with an aqueous solution of K_3_[Fe(CN)_6_], and *E*_1/2_ = +0.458 V vs NHE.

For the *in situ* UV–vis spectroelectrochemical measurements, an Avantes spectrometer was used (Model AvaLight-DHc light source equipped with an AvaSpec-UL2048XL-EVO in the spectroelectrochemical cell kit (AKSTCKIT3)) with a Pt-microstructured honeycomb working electrode obtained from Pine Research Instrumentation (Lyon, France). The cell was positioned in the CUV-UV cuvette holder connected to the diode-array UV–vis spectrometer by optical fibers. The spectra were analyzed by the AvaSoft 8.1.1 software package.

### EPR Spectroscopic studies

The CW-EPR spectra were measured using a BRUKER EleXsys E500 spectrometer (microwave frequency 9.45 GHz, microwave power 13 mW, modulation amplitude 5 G, modulation frequency 100 kHz). A Cu(II)–VLX600 solution was prepared in 0.33 mM CuCl_2_ and 0.5 mM ligand concentration in 30% (v/v) DMSO/water solution, and a titration was performed by KOH solution. Frozen solution EPR spectra were measured for samples of 0.2 mL in quartz EPR tubes and measured in a dewar containing liquid nitrogen (77 K). EPR spectra were simulated by the EPR program.^36^ Axial *g* and *A* tensors (*I*^Cu^= 3/2) were taken into account. For major monoligand complexes, the isotropic nitrogen splitting of three nitrogen atoms was taken into account to describe the nitrogen splitting of the spectra. For the description of the line width, the orientation dependent α, β, and γ parameters were used to set up each component spectra, where α, β, and γ defined the line widths through the equation σ_MI_ = α + β*M*_I_ + γ*M*_I_^2^, where *M*_I_ denotes the magnetic quantum number of the paramagnetic metal ions. A doublet signal appeared in the spectra with low intensity, which can be assigned to a dimeric complex. The copper–copper distance could be estimated from the distance of the doublet lines using the point-dipole approximation. Because a natural CuCl_2_ was applied for the measurements, the spectra were computed as the sum of the spectra of ^63^Cu and ^65^Cu with weights corresponding to their respective natural abundances. The hyperfine and superhyperfine coupling constants, along with the relaxation parameters, were determined in field units (Gauss = 10^–4^ T).

### X-ray Photoelectron Spectroscopy

XPS measurements were performed with a SPECS instrument. The instrument was equipped with a PHOIBOS 150 MCD 9 hemispherical analyzer, which was operated in the FAT mode. A pass energy of 40 eV was used for acquiring survey scans, and 20 eV was used for high-resolution scans. The Al Kα radiation (*h*ν = 1486.6 eV) of a dual-anode X-ray gun was used as an excitation source and operated at 150 W power. Ten scans were averaged to get a single high-resolution spectrum. The carbon 1s peak was used for charge referencing and set at 285.0 eV. For spectrum evaluation, the CasaXPS commercial software package was used.^37^ The solid sample for the XPS study was prepared by stirring the VLX600 ligand and Fe(NO_3_)_3_·9H_2_O in a 2:1 ratio in EtOH for 3 h at 50 °C. The solution was stored at 4 °C for 3 days, and the dark green solid was collected, washed with diethyl ether, and dried in a vacuum.

### Cell Cultures

CH1/PA-1 (provided by L.R. Kelland, CRC Centre for Cancer Therapeutics, Institute of Cancer Research, Sutton, UK; confirmed by STR profiling as PA-1 ovarian teratocarcinoma cells at Multiplexion, Heidelberg, Germany), SW480 colon carcinoma, and A549 non-small-cell lung cancer cells (both obtained from the American Type Culture Collection, Manassas, VA, USA) were maintained in a minimal essential medium (MEM) supplemented with 1 mM sodium pyruvate, 4 mM l-glutamine, 1% (v/v) nonessential amino acids from 100-fold stock (all purchased from Sigma-Aldrich), and 10% heat-inactivated fetal bovine serum (FBS; BioWest, Nuaillé, France).

Human colon Colo205 (chemo-sensitive, ATCC-CCL-222) and Colo320 (doxorubicin-resistant, ATCC-CCL-220.1) adenocarcinoma cell lines, MCF-7 (ATCC HTB-22) breast cancer cells, and MRC-5 (ATCC CCL-171) human normal embryonal lung fibroblast cell lines were purchased from LGC Promochem, Teddington, UK. The cells were cultured in RPMI 1640 medium supplemented with 10% heat-inactivated fetal bovine serum (Colo205 and Colo320) or in minimum essential medium (MEM) supplemented with 10% heat-inactivated fetal bovine serum (MCF-7, MRC-5).

Cells were grown as monolayers in 75 cm^2^ culture flasks (Starlab, Hamburg, Germany) at 37 °C in a humidified atmosphere containing 5% CO_2_.

### *InVitro* Cytotoxicity Assay

The cytotoxic activity of the compounds was determined by the 3-(4,5-dimethylthiazol-2-yl)-2,5-diphenyl-2H-tetrazolium bromide (MTT) assay (obtained from Acros Organics, Geel, Belgium) in at least three independent experiments. A total of 1 × 10^3^ CH1/PA-1, 2 × 10^3^ SW480, and 3 × 10^3^ A549 cells were seeded at 100 μL per well into 96-well microculture plates (Starlab, Hamburg, Germany) 24 h before a treatment with the test compounds for a period of 96 h. Furthermore, 1 × 10^4^ cells of Colo205, Colo320, and MCF-7 as well as MRC-5 were seeded overnight prior to the assay with 72 h exposure to the compounds tested. The stock solution of VLX600 was prepared in DMSO (*c* = 1 mM), and stock solutions were also made with 1 (CuCl_2_) or 0.5 equiv (ZnCl_2_, FeCl_3_) metal chloride salt. In the case of the metal complexes, we first mixed the ligand and the aqueous metal salt solution (*c* = 10 mM) in the appropriate ratio followed by dilution with the medium. The metal salts dissolved in the same way were also tested.

CH1/PA-1, SW480, and A549 cells were treated with 100 μL/well of the test compounds serially diluted in complete MEM. After 96 h exposure, the medium was replaced with 100 μL of MTT solution (5 mg/mL) in phosphate-buffered saline (PBS, Sigma-Aldrich) diluted 1:7 in RPMI 1640 medium (supplemented with 4 mM l-glutamine and 10% heat-inactivated FBS). After 4 h incubation, the MTT-containing medium was replaced with 150 μL DMSO per well to dissolve the formazan product formed by viable cells. Optical densities at 550 nm (and at 690 nm as a reference) were measured with a microplate reader (ELx808, BioTek) by using the Gen5 3.08 software (BioTek).

MTT assays with the cell lines Colo205, Colo320, MCF-7, and MRC-5 were performed after addition of the substances and a subsequent 72 h incubation time. At the end of the incubation period, 20 μL of the MTT solution (from a stock solution of 5 mg/mL) was added to each well. After the staining, the plates were incubated at 37 °C for 4 h, then 100 μL of SDS solution (10% in 0.01 M HCI) was added to each well, and the plates were incubated at 37 °C overnight. The optical density (OD) was determined at 540/630 nm with a Multiscan EX ELISA reader (Thermo Labsystems).

The IC_50_ values, i.e., the concentrations resulting in half the number of viable cells relative to untreated controls, were interpolated from concentration–effect curves of at least three independent experiments.

### Apoptosis Assay

Induction of apoptotic and necrotic cell death was quantitatively analyzed via flow cytometry upon double staining with FITC-conjugated annexin V (eBioscience) and propidium iodide (PI, 1.0 mg/mL, Sigma-Aldrich). CH1/PA-1 cells were seeded into 24-well plates (7 × 10^4^ cells/well) in 600 μL MEM per well and allowed to settle for 24 h. After 24 h preincubation, cells were treated with different concentrations of the test compounds at concentrations of 0.2, 1, 5, and 25 μM for 24 h. After treatment, the medium was collected, and cells were washed once with 37 °C PBS and trypsinized for 5 min. Following trypsinization, the cell suspension was added to the precollected medium, and cells were pelleted by centrifugation (300*g*, 3 min). The supernatant was removed, and the cell pellet was resuspended with 1.5 μL FITC-conjugated annexin V in 150 μL binding buffer (10 mM HEPES/NaOH pH 7.4, 140 mM NaCl, and 2.5 mM CaCl_2_) and incubated at 37 °C for 15 min. Cells were subsequently stained with PI (1.0 μL in 150 μL binding buffer) and analyzed with a Guava easyCyte 8 HT flow cytometer (Millipore) and the InCyte software. Results were quantified by using the FlowJo software 10.6.1 (TreeStar). A minimum of three independent experiments were carried out.

### Computational Methods

The geometry of the bis-chelated [Fe(II)(LH)_2_]^2+^ complexes was optimized through the Gaussian 16 (rev. B.01)^38^ software at the DFT level of theory using the hybrid B3LYP functional^39,40^ with the D3 version of Grimme’s dispersion including BJ-damping^41^ to better describe noncovalent interactions. This functional was combined with the 6-311g(d) basis set for main group elements and LANL2DZ for Fe(II). The effect of the solvent was taken into account by adopting the polarizable continuum model (PCM)^42^ for water. Single-point frequency calculations were carried out with the same functional and basis sets for the ground-state geometries, which represented true minima on the potential energy surface (PES); thus, no imaginary frequencies were found. The relative free energies of the coordination isomers with spin state *S* = 1/2 were calculated at the same level of theory.

Ground-state geometries of the Cu(II) and Zn(II) complexes were computed through the ORCA software (v. 5.0.3.).^43^ The B3LYP functional with the atom-pairwise dispersion correction including the Becke–Johnson damping scheme (B3LYP D3BJ) was utilized in the calculations. For Zn(II) complexes, the DKH-def2-TZVP basis set was used according to the method published earlier.^44^ The resolution of identity and chain of spheres exchange (RIJCOSX) approximation^45^ was utilized to accelerate the calculations with the auxiliary basis sets generated through the AutoAux procedure.^46^ For Cu(II) complexes, the B3LYP D3BJ was combined with the core-property basis set (CP(PPP)^47^ for Cu(II) and the 6-311g(d,p) for main group elements. In the calculations of Cu(II) hyperfine coupling (*A* tensor), the nitrogen atoms were treated with the EPR-III functional. In all calculations, the effect of the solvent was considered by adopting the PCM method, and single-point calculations for the ground-state geometries represented true minima on PES.

## Supporting Information Available

Additional experimental and computational (DFT) details, and spectral (NMR and EPR) and structural characterization of the compounds along with results showing the ability of VLX600 to induce ROS production (PDF)

## ABBREVIATIONS

CV: cyclic voltammetry
D_7.4_: distribution coefficients
DFT: density functional theor;
DMF: dimethylformamide
DMSO: dimethyl sulfoxide
EPR: electron paramagnetic resonance
EXAFS: extended X-ray absorption fine structure
HEPES: *N*-2-hydroxyethylpiperazine-*N*-2-ethanesulfonic acid
MDR: multidrug resistance
MEM: minimal essential medium
MES: 2-(*N*-morpholino)ethanesulfonic acid
MTT: 3-(4,5-dimethylthiazol-2-yl)-2,5-diphenyl-2*H*-tetrazolium bromide
PAMPA: parallel, artificial membrane permeability assay
PBS: phosphate-buffered saline
*P*_effective_: effective passive permeability
p*K*_a_: proton dissociation constant
TBAN: tetrabutylammonium nitrate
TSC: thiosemicarbazone
UV–vis: UV–visible
VLX600: 6-methyl-3-{(2*E*)-2-[1-(2-pyridinyl)ethylidene]hydrazino}-5*H*-[1,2,4]triazino[5,6-*b*]indole
β: overall protonation constant
ε: molar absorbance
XPS: X-ray photoelectron spectroscopy
