# Supplementary material for: A Comparative Study on the Complexation of the Anticancer Iron Chelator VLX600 with Essential Metal Ions
Source: Inorg Chem. 2024 Jan 24;63(5):2401–17. doi: 10.1021/acs.inorgchem.3c03259 (PMC10848209; doi:10.1021/acs.inorgchem.3c03259)
Supplement: Supplementary file 1 — ic3c03259_si_001.pdf [file ic3c03259_si_001.pdf]

SUPPORTING INFORMATION

A comparative study on complexation of the anticancer iron chelator VLX600 with essential metal ions

Vivien Pósa,[a,b] Anja Federa,[c,d] Klaudia Cseh,[c] Dominik Wenisch,[c] Gabriella Spengler,[a,e] Nóra V. May,[f] Norbert Lihi,[g] Gergely F. Samu,[b] Michael A. Jakupec,[c,d] Bernhard K. Keppler,[c,d] Christian R. Kowol,[c,d] Éva A. Enyedy[a,b,*]

[a] *MTA-SZTE Lendület Functional Metal Complexes Research Group, University of Szeged, Dóm tér 7, H-6720 Szeged, Hungary*
[b] *Department of Molecular and Analytical Chemistry, Interdisciplinary Excellence Centre, University of Szeged, Dóm tér 7-8, H-6720 Szeged, Hungary*
[c] *Institute of Inorganic Chemistry, Faculty of Chemistry, University of Vienna, Waehringer Strasse 42, A-1090 Vienna, Austria*
[d] *Research Cluster ''Translational Cancer Therapy Research'', Waehringer Strasse 42, A-1090 Vienna, Austria*
[e] *Department of Medical Microbiology, Albert Szent-Györgyi Health Center and Albert Szent-Györgyi Medical School, University of Szeged, Semmelweis utca 6, H-6725 Szeged, Hungary*
[f] *Centre for Structural Science, Research Centre for Natural Sciences, Hungarian Research Network (HUN-REN),, Magyar tudósok körútja 2, H-1117 Budapest, Hungary*
[g] *ELKH-DE Mechanisms of Complex Homogeneous and Heterogeneous Chemical Reactions Research Group, Department of Inorganic and Analytical Chemistry, University of Debrecen, Egyetem tér 1., H-4032 Debrecen, Hungary*

Contents

Solution stability of VLX600 and pH-dependence of chemical shifts of its CH protons SI-2
Spectra of iron complexes of VLX600 ....SI-3
Table of the equilibrium processes associated with the equilibrium constants SI-3
Concentration distribution curves for iron complexes of VLX600 ....SI-4
X-ray structures' experimental data SI-5
DFT calculations for the $[Fe(II)(LH)_2]^{2+}$ complexes SI-6
Cu(II) complexes of VLX600: concentration distribution curves, additional X-ray structures SI-15
DFT calculations for the Cu(II) complexes of VLX600 ....SI-17
Cu(II) complexes of VLX600: EPR spectroscopy and CV in DMF/H_2O solvent mixture SI-33
DFT calculations for the $[Zn(LH)Cl_2]$ and $[Zn(LH)(H_2O)_2]^{2+}$ complexes SI-33
The ability of VLX600 to induce ROS production (DCFH/DA assay) SI-39
NMR spectra of VLX600 ....SI-40

Solution stability of VLX600 and pH-dependence of chemical shifts of its CH protons

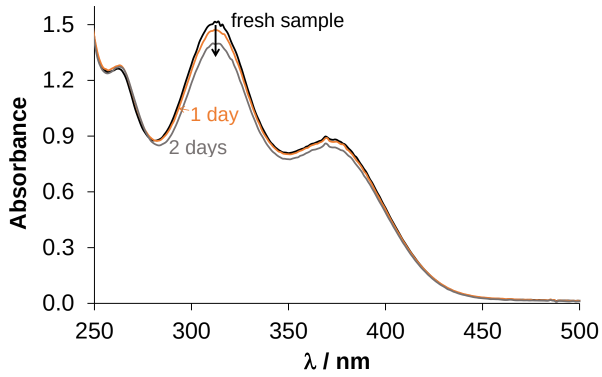

Figure S1 UV-vis spectra recorded for VLX600 dissolved in 30% (*v*/*v*) DMSO/H_2O freshly, after 1 and 2 days. {c_{VLX600} = 60 µM; *I* = 0.10 M KCl; pH = 6.5; ℓ = 1 cm; *t* = 25.0 °C}

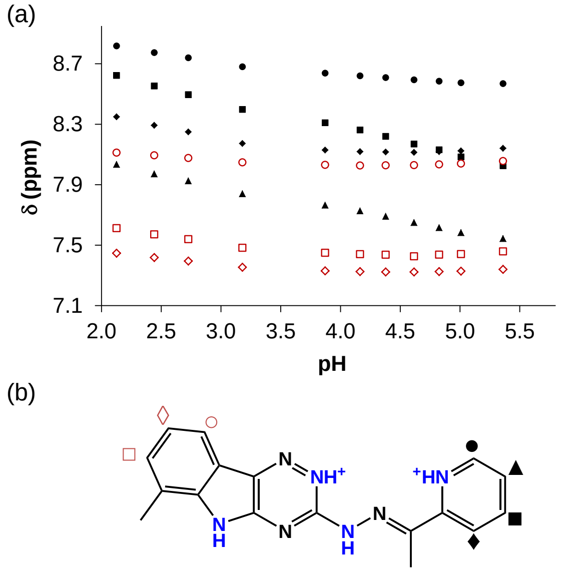

Figure S2 (a) Chemical shifts (δ) of various CH protons of VLX600 plotted against the pH. (b) VLX600 in its fully protonated state (H_3L^{2+}) and peak assignation with symbols. {c_{VLX600} = 1 mM; 30% (*v*/*v*) DMSO-d_6/H_2O, *I* = 0.1 M KCl}

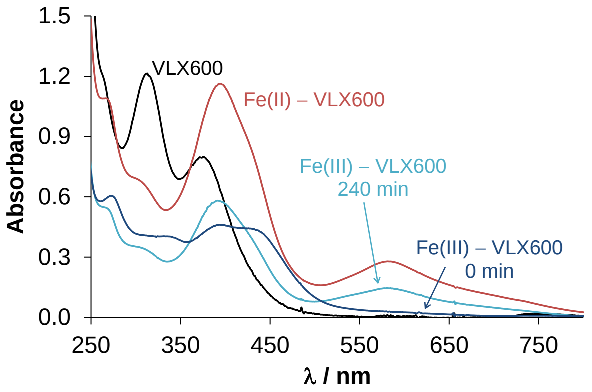

Figure S3 UV-vis spectra of VLX600, Fe(II) – VLX600 (1:2) system, and Fe(III) – VLX600 (1:2) system at 0 min and 240 min. {c_{VLX600} = 50 μM; $c_{Fe(II)}$ = 25 μM; I = 0.10 M KCl; ℓ = 0.5 cm; t = 25.0 °C} The spectra for the Fe(III) containing spectra were calculated from data in Figure 4.

Table S1. Equilibrium processes associated with the overall protonation (β) and proton dissociation constants (K_a) of VLX600 and the overall stability (formation) constants (β) of its complexes. HL denotes the neutral form of VLX600. (The coordinated solvent molecules are not labelled for simplicity.)

	**equilibrium process**
β **H_3L^{2+}**	$L^- + 3\,H^+ \rightleftharpoons H_3L^{2+}$
β **H_2L^+**	$L^- + 2\,H^+ \rightleftharpoons H_2L^+$
β **HL**	$L^- + H^+ \rightleftharpoons HL$
K_a **H_3L^{2+}**	$H_3L^{2+} \rightleftharpoons H_2L^+ + H^+$
K_a **H_2L^+**	$H_2L^+ \rightleftharpoons HL + H^+$
K_a **HL**	$HL \rightleftharpoons L^- + H^+$
β **$[M(LH)]^{2+}$**	$M^{2+} + L^- + H^+ \rightleftharpoons [M(LH)]^{2+}$
β **$[M(L)]^+$**	$M^{2+} + L^- \rightleftharpoons [M(L)]^+$
β **$[M(L)H_{-1}]$**	$M^{2+} + L^- \rightleftharpoons [M(L)H_{-1}] + H^+$
β **$[M(LH)_2]^{2+}$**	$M^{2+} + 2\,L^- + 2\,H^+ \rightleftharpoons [M(LH)_2]^{2+}$
β **$[M(L)(LH)]^+$**	$M^{2+} + 2\,L^- + H^+ \rightleftharpoons [M(L)(LH)]^+$
β **$[M(L)_2]$**	$M^{2+} + 2\,L^- \rightleftharpoons [M(L)_2]$

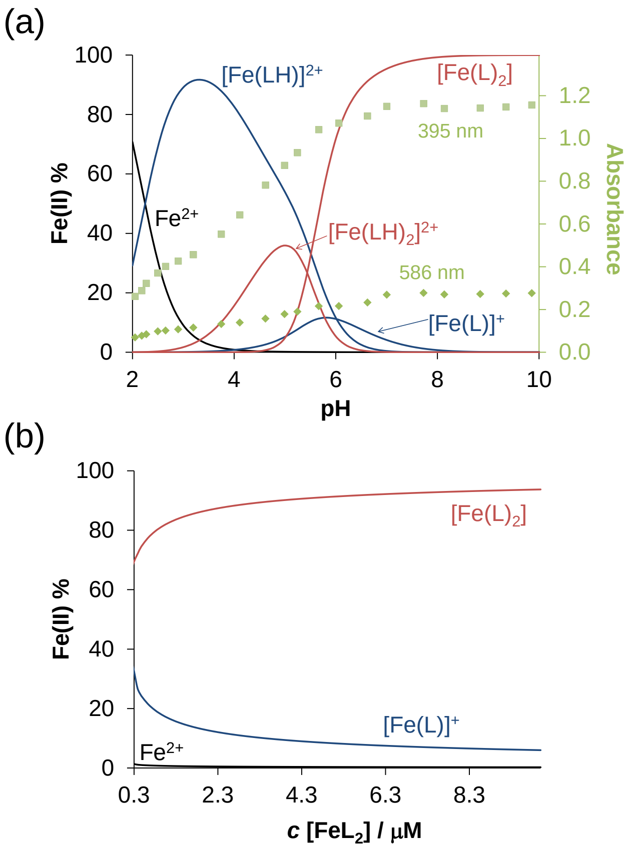

Figure S4 (a) Concentration distribution curves of the Fe(II) – VLX600 (1:2) system calculated with the determined formation constants (log*β*) and absorbance values at 395 nm (■) and 586 nm (♦). {c_{VLX600} = 50 μM; $c_{Fe(II)}$ = 25 μM; *I* = 0.10 M KCl; ℓ = 0.5 cm; *t* = 25.0 °C} (b) Concentration distribution curves of the Fe(II) – VLX600 (1:2) system to present the distribution of complex [FeL_2] at pH 7.4.

X-ray structures experimental data

Table S2 Experimental, sample and crystal data, data collection and structure refinement parameters

	**[Fe(II)(LH)$_2$](NO$_3$)$_2$**	**[Cu(L)ClMeOH]**	**[Cu(LH)Cl$_2$]**	**[Zn(LH)Cl$_2$]**	**HL·3H$_2$O**
Empirical formula	$C_{34}H_{38}FeN_{16}O_{10}$	$C_{17}H_{15}Cl_2CuN_7$	$C_{19}H_{22}ClCuN_7O_2$	$C_{21}H_{27}Cl_2N_7O_2S_2Zn$	$C_{17}H_{21}N_7O_3$
CCDC code	2245566	2245574	2261411	2245575	2245577
Formula weight	886.65	451.80	479.42	609.88	371.402
Temperature	100 K	100 K	100 K	100 K	100 K
Detector distance [mm]	40.0	40.0	40.0	40.0	50.0
Number of frames	1627	3160	9070	5062	3621
Wavelength	0.71073 Å	0.71073 Å	0.71073 Å	0.71073 Å	0.71073 Å
Crystal system	Monoclinic	Triclinic	Monoclinic	Triclinic	Monoclinic
Space group	C2/c	P-1	P 21/c	P-1	P 1 21/c 1
Unit cell dimensions [Å]	a = 26.412(2)<br>b = 10.7562(7)<br>c = 18.8759(2)	a = 7.5666(7)<br>b = 9.9681(12)<br>c = 12.2519(13)	a = 7.0597(4)<br>b = 16.0274(8)<br>c = 18.0208(8)	a = 8.9522(6)<br>b = 10.5420(7)<br>c = 14.8840(10)	a = 12.7354(16)<br>b = 6.9263(5)<br>c = 20.524(3)
Unit cell dimensions [°]	α = 90°<br>β = 123.374(5)°<br>γ = 90°	α = 87.290(9)°<br>β = 73.479(8)°<br>γ = 84.166(8)°	α = 90°<br>β = 91.131(4)°<br>γ = 90°	α = 80.114(6)°<br>β = 77.951(5)°<br>γ = 71.034(5)°	α = 90°<br>β = 94.026(10)°<br>γ = 90°
Volume	4478.3(6) Å^3	881.19(16) Å^3	2038.62(18) Å^3	1290.92(15) Å^3	1806.0(3) Å^3
Z	4	2	4	2	4
Density (calculated)	1.315 Mg/m^3	1.703 Mg/m^3	1.562 Mg/m^3	1.569 Mg/m^3	1.366 Mg/m^3
Absorption coefficient	0.406 mm^{-1}	1.561 mm^{-1}	1.235 mm^{-1}	1.354 mm^{-1}	0.098 mm^{-1}
F(000)	1840	458	988.0	628	784.556
Crystal size	0.210 x 0.123 x 0.070 mm^3	0.150 x 0.150 x 0.150 mm^3	0.200 x 0.060 x 0.060 mm^3	0.090 x 0.050 x 0.040 mm^3	0.15 x 0.103 x 0.03 mm^3

	$[Fe(II)(LH)_2](NO_3)_2$	[Cu(L)ClMeOH]	$[Cu(LH)Cl_2]$	$[Zn(LH)Cl_2]$	$HL·3H_2O$
Theta range for data collection	2.107 to 27.098°	2.054 to 31.63°	2.78 to 28.58°	2.36 to 29.78°	1.99 to 25.68°
Index ranges	-33 <= h < =33, -13 <= k <= 13, -23 <= l <=24	-11 <= h <= 9, -14 <= k <= 14, -17 <= l <= 17	-9 <= h < =9, -20 <= k <= 21, -24 <= l <=23	-10 <= h <= 12, -14 <= k <= 14, -16 <= l <= 20	-17 <= h <= 17, -8 <= k <= 9, -29 <= l <= 20
Reflections collected	27665	45063	49204	54449	27830
Independent reflections	4942 [R(int) = 0.0816]	5663 [R(int) = 0.0713]	5058 [R(int) = 0.0569]	7034 [R(int) = 0.0996]	3424 [R(int) = 0.0995]
Completeness to theta = 25.242°	99.9 %	99.9 %	99.9 %	99.7 %	99.76 %
Absorption correction	Semi-empirical from equivalents	Semi-empirical from equivalents	Semi-empirical from equivalents	Semi-empirical from equivalents	Semi-empirical from equivalents
Max. and min. transmission	0.9937 and 0.9813	0.4885 and 0.3449	0.9283 and 0.7804	0.9205 and 0.2802	0.9976 and 0.9881
Refinement method	Full-matrix least-squares on F^2	Full-matrix least-squares on F^2	Full-matrix least-squares on F^2	Full-matrix least-squares on F^2	Full-matrix least-squares on F^2
Data / restraints / parameters	4942 / 845 / 341	5663 / 0 / 247	5058 / 0 / 277	7034 / 0 / 322	3424 / 1 / 256
Goodness-of-fit on F^2	0.961	0.889	0.913	0.475	0.7959
Final R indices [I>2sigma(I)]	R1 = 0.0468, wR2 = 0.1213	R1 = 0.0653, wR2 = 0.1602	R1 = 0.0318 wR2 = 0.0737	R1 = 0.0351, wR2 = 0.0962	R1 = 0.0507, wR2 = 0.1163
R indices (all data)	R1 = 0.0771, wR2 = 0.1310	R1 = 0.1340, wR2 = 0.1898	R1 = 0.0577 wR2 = 0.0779	R1 = 0.0963, wR2 = 0.1050	R1 = 0.1021, wR2 = 0.1268
Extinction coefficient	n/a	0.038(5)	n/a	n/a	0.022(3)
Largest diff. peak and hole	0.493 and -0.769 e.Å^{-3}	1.093 and -2.242 e.Å^{-3}	0.524 and -0.649 e.Å^{-3}	0.495 and -0.690 e.Å^{-3}	0.6376 and -0.4008 e.Å^{-3}

DFT calculations for the $[Fe(II)(LH)_2]^{2+}$ complexes

For the bis-chelated iron(II) complexes, the formation of four coordination isomers is plausible. Iron(II) coordinates via the pyridine, imine N and the N^2 of the ligand or the pyridine, imine N and the N^4 donor set. However, the donor atoms of pyridine and N^2 or N^4 of the second ligand can alter the axial positions of the octahedral iron(II) leading to the formation of further coordination isomers. These binding modes together with the optimized structures are shown in Figure S3 and the ΔG values for the isomerisation reactions are reported in Table S3. Cartesian coordinates and energy values are summarized in Table S4-S7.

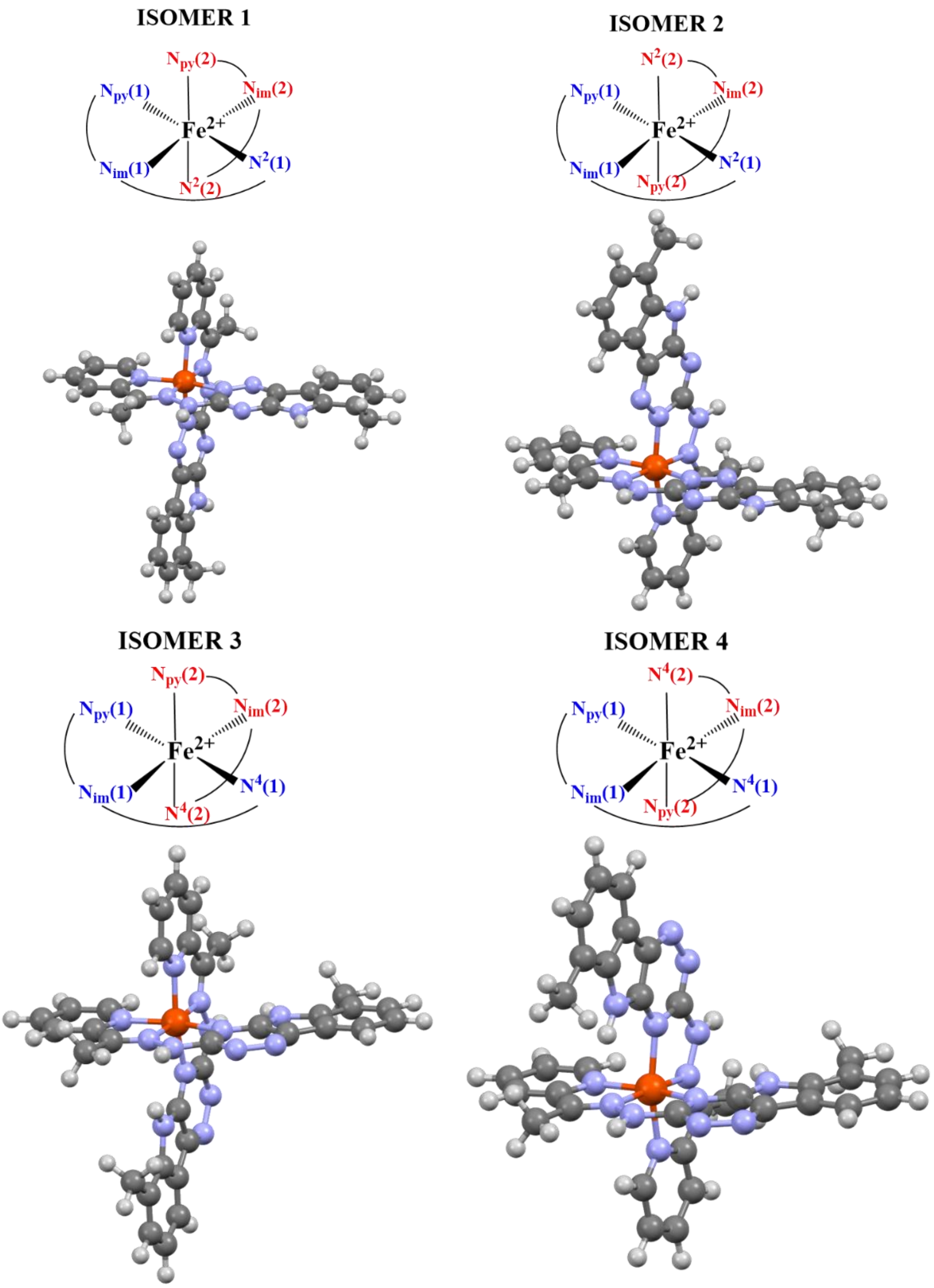

Figure S5 Binding modes and DFT optimized structures of $[Fe(II)(LH)_2]^{2+}$ complexes.

The relative energies clearly indicate that iron(II) prefers the pyridine, imine N and the N^2 coordination mode, the energy gap for the formation of N^4 coordinated species was calculated to be 37.5 kJ/mol. The results of the DFT calculations of Isomer 1 are in good agreement with the experimental data obtained by X-ray crystallography. The two structures overlap confirming that the geometry optimization was reasonably accurate (Figure S4).

Table S3 Selected bond lengths, angles and the relative energy values obtained from DFT calculations. Selected parameters from X-ray crystallographic studies are also shown (taken from Figure 6).

	**Isomer 1**	**Isomer 2**	**Isomer 3**	**Isomer 4**	**X-ray**
Bond lengths (Å)					
Fe - $_{Npy}$	1.9794	1.9794	1.9845	1.9845	1.9614
Fe - $_{Nimine}$	1.9050	1.9050	1.9243	1.9243	1.8814
Fe - N^2	1.9588	1.9588			1.9288
Fe - N^4			1.9929	1.9929	
Angles (˚)					
N_{py} - Fe- N_{imine}	80.7	80.7	80.8	80.9	80.51
N_{imine} - Fe- N^2	82.1	82.1			81.33
N_{imine} - Fe- N^4			81.5	81.5	
N_{py} - Fe- N^2	162.8	162.8			161.82
N_{py} - Fe- N^4			162.3	162.3	
ΔG_{rel} (kJ/mol)					
Isomer 1 → Isomer 2	-0.05				
Isomer 3 → Isomer 4	-0.01				
Isomer 2 → Isomer 3	-37.5				

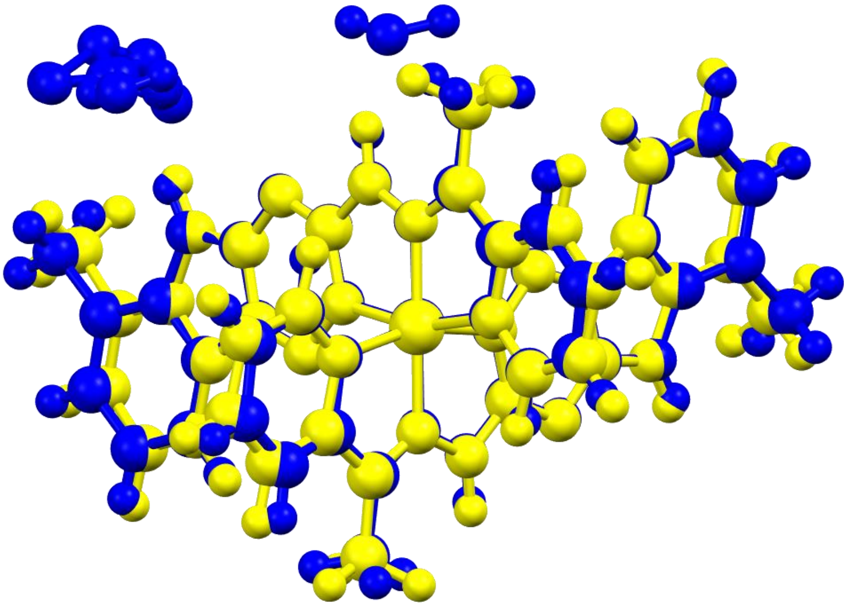

Figure S6 Comparison of the structures obtained from DFT calculation (yellow) and X-ray crystallography (blue).

Table S4 Cartesian coordinates and energy values of $[Fe(II)(LH)_2]^{2+}$, Isomer 1.

Sum of electronic and thermal Energies (Eh)	-2202.888896
Sum of electronic and enthalpy Energies (Eh)	-2202.887952
Sum of electronic and thermal Free Energies (Eh)	-2203.001208
Number of Imaginary Frequencies	0

Molecular Geometry in Cartesian Coordinates

N	2.148725	0.518361	0.331378
C	-3.458741	-4.726693	-0.502040
H	-4.250696	-5.440719	-0.668535
C	5.432164	3.461612	1.812509
H	5.844253	3.631032	2.796709
C	-2.934790	-4.534660	0.775134
H	-3.304724	-5.090878	1.621651
N	0.370980	-0.563379	-2.576783
N	-0.362248	-1.477702	-1.869606
N	2.030756	1.088633	-2.460039
C	-1.913747	-3.602445	0.952668
H	-1.477194	-3.418131	1.920250
N	-1.421714	-2.884937	-0.073768
C	-1.927199	-3.067707	-1.341911
C	4.432069	3.089950	-0.735191
C	5.426631	4.056901	-0.577889
C	4.438370	2.500623	1.634396
H	4.066925	1.916163	2.463125
C	3.933523	2.313630	0.345350
C	-2.950008	-3.986841	-1.571530
H	-3.346316	-4.126512	-2.563779
C	-1.304510	-2.228359	-2.372850
C	5.910871	4.218524	0.728470
H	6.682775	4.955820	0.901812
C	5.938551	4.867560	-1.739508
H	6.365414	4.229159	-2.516923
H	6.714431	5.558095	-1.414671
H	5.141159	5.455916	-2.200290
N	3.757537	2.705615	-1.922790
H	3.909408	3.091568	-2.839570
N	1.297163	-0.130729	-0.502771
C	2.863370	1.727457	-1.634357
C	2.930844	1.441358	-0.220901
C	-1.712220	-2.194407	-3.808109
H	-2.369874	-3.018975	-4.061303
H	-0.844451	-2.256926	-4.467607
H	-2.241548	-1.265467	-4.036135
C	1.270053	0.166236	-1.838243
H	0.237475	-0.386827	-3.560963
N	-2.148471	0.518598	-0.331387
C	3.458484	-4.726993	0.501757
H	4.250370	-5.441107	0.668206
C	-5.431648	3.462190	-1.812423
H	-5.843584	3.631815	-2.796652
C	2.934595	-4.534783	-0.775416
H	3.304516	-5.090941	-1.621979
N	-0.371110	-0.563698	2.576792
N	0.362124	-1.477968	1.869553
N	-2.030895	1.088324	2.460158
C	1.913638	-3.602463	-0.952890

H	1.477117	-3.418030	-1.920465
N	1.421615	-2.885039	0.073609
C	1.927038	-3.067978	1.341752
C	-4.431940	3.090002	0.735348
C	-5.426483	4.056982	0.578097
C	-4.437868	2.501178	-1.634362
H	-4.066288	1.916901	-2.463160
C	-3.933218	2.313915	-0.345277
C	2.949774	-3.987212	1.571308
H	3.346032	-4.127037	2.563554
C	1.304357	-2.228693	2.372752
C	-5.910527	4.218874	-0.728300
H	-6.682398	4.956212	-0.901612
C	-5.938620	4.867344	1.739826
H	-6.366315	4.228775	2.516649
H	-6.713908	5.558495	1.414885
H	-5.141174	5.455003	2.201396
N	-3.757597	2.705420	1.922975
H	-3.909695	3.091102	2.839831
N	-1.297060	-0.130681	0.502763
C	-2.863385	1.727321	1.634478
C	-2.930632	1.441525	0.220953
C	1.711875	-2.194953	3.808072
H	2.239702	-1.265314	4.036707
H	2.370834	-3.018596	4.060861
H	0.844117	-2.259253	4.467423
C	-1.270094	0.166065	1.838293
H	-0.238198	-0.387779	3.561169
Fe	-0.000011	-1.510371	-0.000037

Table S5 Cartesian coordinates and energy values of $[Fe(II)(LH)_2]^{2+}$, Isomer 2.

Sum of electronic and thermal Energies (Eh)	-2202.888895
Sum of electronic and enthalpy Energies (Eh)	-2202.887951
Sum of electronic and thermal Free Energies (Eh)	-2203.001188
Number of Imaginary Frequencies	0

Molecular Geometry in Cartesian Coordinates

N	2.148690	0.518515	-0.331373
C	-3.458201	-4.727193	0.501654
H	-4.250000	-5.441411	0.668071
C	5.432566	3.461441	-1.812129
H	5.844793	3.630817	-2.796279
C	-2.934253	-4.534949	-0.775498
H	-3.304035	-5.091198	-1.622063
N	0.370593	-0.563143	2.576649
N	-0.362309	-1.477780	1.869525
N	2.030546	1.088703	2.460073
C	-1.913396	-3.602520	-0.952932
H	-1.476816	-3.418045	-1.920471
N	-1.421513	-2.885024	0.073592
C	-1.927031	-3.067946	1.341676
C	4.432129	3.089891	0.735442
C	5.426804	4.056747	0.578263
C	4.438626	2.500584	-1.634140
H	4.067206	1.916178	-2.462919
C	3.933609	2.313644	-0.345151

C	-2.949662	-3.987311	1.571210
H	-3.345975	-4.127132	2.563436
C	-1.304559	-2.228518	2.372689
C	5.911228	4.218307	-0.728033
H	6.683217	4.955531	-0.901300
C	5.938729	4.867265	1.739977
H	5.141032	5.454296	2.201904
H	6.713443	5.559007	1.414926
H	6.367098	4.228841	2.516553
N	3.757444	2.705608	1.922977
H	3.909449	3.091347	2.839823
N	1.296999	-0.130556	0.502720
C	2.863226	1.727520	1.634460
C	2.930803	1.441439	0.220996
C	-1.712543	-2.194504	3.807868
H	-2.370362	-3.018974	4.060947
H	-2.241768	-1.265479	4.035775
H	-0.844907	-2.257237	4.467520
C	1.269809	0.166354	1.838182
H	0.237626	-0.387144	3.560999
N	-2.148966	0.518272	0.331355
C	3.458903	-4.726430	-0.501867
H	4.250888	-5.440428	-0.668339
C	-5.432770	3.461141	1.812307
H	-5.844969	3.630477	2.796475
C	2.934747	-4.534587	0.775256
H	3.304553	-5.090928	1.621751
N	-0.370878	-0.563221	-2.576758
N	0.362361	-1.477537	-1.869609
N	-2.030871	1.088583	-2.460085
C	1.913664	-3.602422	0.952754
H	1.476923	-3.418252	1.920278
N	1.421775	-2.884788	-0.073676
C	1.927453	-3.067357	-1.341753
C	-4.432406	3.089695	-0.735311
C	-5.427045	4.056574	-0.578057
C	-4.438868	2.500257	1.634240
H	-4.067437	1.915798	2.462977
C	-3.933889	2.313376	0.345229
C	2.950329	-3.986440	-1.571331
H	3.346804	-4.125983	-2.563528
C	1.304894	-2.227910	-2.372724
C	-5.911430	4.218080	0.728263
H	-6.683394	4.955318	0.901585
C	-5.938939	4.867215	-1.739699
H	-5.141321	5.454683	-2.201210
H	-6.714018	5.558566	-1.414685
H	-6.366827	4.228855	-2.516590
N	-3.757748	2.705465	-1.922881
H	-3.909697	3.091331	-2.839683
N	-1.297207	-0.130700	-0.502792
C	-2.863540	1.727350	-1.634438
C	-2.931093	1.441192	-0.220970
C	1.712982	-2.193519	-3.807865
H	2.371736	-3.017254	-4.060873
H	2.241184	-1.263917	-4.035794
H	0.845430	-2.257216	-4.467534
C	-1.270069	0.166257	-1.838210
H	-0.237164	-0.386356	-3.560859
Fe	0.000002	-1.510277	-0.000041

Table S6 Cartesian coordinates and energy values of $[Fe(II)(LH)_2]^{2+}$, Isomer 3.

Sum of electronic and thermal Energies (Eh)	-2202.875744
Sum of electronic and enthalpy Energies (Eh)	-2202.8748
Sum of electronic and thermal Free Energies (Eh)	-2202.98691
Number of Imaginary Frequencies	0

Molecular Geometry in Cartesian Coordinates

N	-1.938278	-2.080793	-3.033431
C	3.252906	4.414927	1.017472
H	4.010456	5.162639	1.194241
C	-5.499142	-4.114974	-0.839492
H	-6.199317	-4.868500	-1.169476
C	2.327019	4.094487	2.007823
H	2.344553	4.581996	2.969332
N	0.695464	0.311570	-2.591910
N	1.083557	1.164944	-1.588637
N	-0.947091	-0.298585	-1.065279
C	1.361009	3.124029	1.745471
H	0.627616	2.851384	2.484660
N	1.297702	2.486120	0.561776
C	2.209728	2.792621	-0.421489
C	-3.752650	-2.191849	0.076652
C	-4.734000	-2.625816	0.966150
C	-4.507931	-3.664234	-1.710929
H	-4.422664	-4.053168	-2.714533
C	-3.623456	-2.689299	-1.244791
C	3.194191	3.755644	-0.212073
H	3.903160	3.986126	-0.990586
C	2.059977	2.032658	-1.667106
C	-5.605572	-3.605359	0.466434
H	-6.386413	-3.977862	1.115130
C	-4.839799	-2.069253	2.361590
H	-3.916514	-2.222555	2.925660
H	-5.646811	-2.551240	2.909568
H	-5.040851	-0.994844	2.349313
N	-2.740200	-1.218548	0.298015
H	-2.586294	-0.728785	1.161134
N	-0.846262	-1.285791	-3.288527
C	-1.992968	-1.091750	-0.821525
C	-2.495650	-1.994801	-1.829532
C	2.944575	2.255054	-2.847112
H	3.975822	1.990066	-2.605718
H	2.932129	3.305079	-3.140248
H	2.650244	1.669950	-3.713551
C	-0.411886	-0.460984	-2.322027
H	1.107035	0.311990	-3.512815
N	1.937680	-2.081706	3.032981
C	-3.252060	4.415936	-1.016734
H	-4.009463	5.163834	-1.193347
C	5.498787	-4.115494	0.839080
H	6.198863	-4.869151	1.168974
C	-2.326203	4.095564	-2.007130
H	-2.343604	4.583318	-2.968517
N	-0.695933	0.310877	2.591683
N	-1.083546	1.164846	1.588715
N	0.946832	-0.299007	1.065097
C	-1.360408	3.124838	-1.744988

H	-0.627076	2.852221	-2.484243
N	-1.297279	2.486599	-0.561457
C	-2.209267	2.793049	0.421869
C	3.752561	-2.192011	-0.076831
C	4.734036	-2.625791	-0.966282
C	4.507443	-3.664950	1.710466
H	4.421969	-4.054168	2.713943
C	3.623111	-2.689828	1.244449
C	-3.193516	3.756336	0.212648
H	-3.902487	3.986754	0.991177
C	-2.059821	2.032688	1.667291
C	5.605472	-3.605515	-0.466683
H	6.386401	-3.977883	-1.115350
C	4.840063	-2.068909	-2.361576
H	3.916953	-2.222324	-2.925898
H	5.647323	-2.550602	-2.909449
H	5.040865	-0.994458	-2.349041
N	2.740148	-1.218647	-0.298112
H	2.586544	-0.728422	-1.161019
N	0.845669	-1.286694	3.288110
C	1.992762	-1.092102	0.821342
C	2.495249	-1.995430	1.829200
C	-2.944676	2.254700	2.847181
H	-3.975638	1.988476	2.605894
H	-2.933374	3.304867	3.139815
H	-2.649716	1.670364	3.713925
C	0.411467	-0.461641	2.321755
H	-1.107304	0.311420	3.512681
Fe	0.000010	1.094306	0.000066

Table S7 Cartesian coordinates and energy values of $[Fe(II)(LH)_2]^{2+}$, Isomer 4.

Sum of electronic and thermal Energies (Eh)	-2202.875744
Sum of electronic and enthalpy Energies (Eh)	-2202.8748
Sum of electronic and thermal Free Energies (Eh)	-2202.986912
Number of Imaginary Frequencies	0

Molecular Geometry in Cartesian Coordinates

N	-1.938471	2.080939	-3.033218
C	3.253132	-4.415013	1.016902
H	4.010700	-5.162744	1.193511
C	-5.499558	4.114691	-0.839266
H	-6.199714	4.868248	-1.169222
C	2.327404	-4.094622	2.007408
H	2.345095	-4.582167	2.968895
N	0.695687	-0.310961	-2.591712
N	1.083489	-1.164808	-1.588696
N	-0.947068	0.298828	-1.065116
C	1.361351	-3.124149	1.745238
H	0.628085	-2.851565	2.484570
N	1.297872	-2.486156	0.561609
C	2.209775	-2.792587	-0.421807
C	-3.753063	2.191499	0.076784
C	-4.734466	2.625392	0.966284
C	-4.508335	3.664013	-1.710713
H	-4.423084	4.053011	-2.714295
C	-3.623858	2.689055	-1.244641

C	3.194243	-3.755642	-0.212591
H	3.903112	-3.986088	-0.991205
C	2.059947	-2.032429	-1.667309
C	-5.606019	3.604975	0.466610
H	-6.386902	3.977393	1.115302
C	-4.840263	2.068929	2.361778
H	-5.038720	0.994043	2.349694
H	-5.649006	2.549095	2.908806
H	-3.917918	2.224710	2.926716
N	-2.740396	1.218416	0.298158
H	-2.586650	0.728337	1.161122
N	-0.846337	1.286146	-3.288301
C	-1.993138	1.091754	-0.821379
C	-2.495910	1.994779	-1.829347
C	2.944700	-2.254400	-2.847263
H	2.933097	-3.304494	-3.140168
H	3.975756	-1.988564	-2.605896
H	2.649931	-1.669720	-3.713839
C	-0.411854	0.461363	-2.321809
H	1.106668	-0.311947	-3.512892
N	1.937975	2.081252	3.033256
C	-3.251694	-4.415798	-1.017299
H	-4.009029	-5.163715	-1.194120
C	5.498664	4.115673	0.839184
H	6.198782	4.869263	1.169147
C	-2.325822	-4.095149	-2.007591
H	-2.343194	-4.582667	-2.969100
N	-0.695603	-0.311285	2.591856
N	-1.083375	-1.165175	1.588860
N	0.946990	0.298860	1.065212
C	-1.360086	-3.124430	-1.745177
H	-0.626700	-2.851565	-2.484293
N	-1.297091	-2.486481	-0.561499
C	-2.209061	-2.793211	0.421758
C	3.752352	2.192373	-0.076804
C	4.733641	2.626322	-0.966332
C	4.507417	3.664984	1.710632
H	4.422076	4.054072	2.714170
C	3.623036	2.689937	1.244580
C	-3.193250	-3.756497	0.212260
H	-3.902229	-3.987192	0.990699
C	-2.059662	-2.033025	1.667312
C	5.605178	3.605949	-0.466680
H	6.385987	3.978452	-1.115414
C	4.839472	2.069571	-2.361685
H	5.042333	0.995498	-2.349209
H	5.645298	2.552769	-2.910342
H	3.915553	2.221126	-2.925172
N	2.739967	1.218966	-0.298156
H	2.586527	0.728811	-1.161139
N	0.846093	1.286139	3.288415
C	1.992761	1.092160	0.821385
C	2.495293	1.995331	1.829329
C	-2.944397	-2.255467	2.847201
H	-2.933415	-3.305816	3.139173
H	-3.975325	-1.988754	2.606276
H	-2.649144	-1.671795	3.714297
C	0.411784	0.461231	2.321898
H	-1.106950	-0.311765	3.512865
Fe	0.000099	-1.094324	0.000127

Cu(II) complexes of VLX600: concentration distribution curves, additional X-ray structures

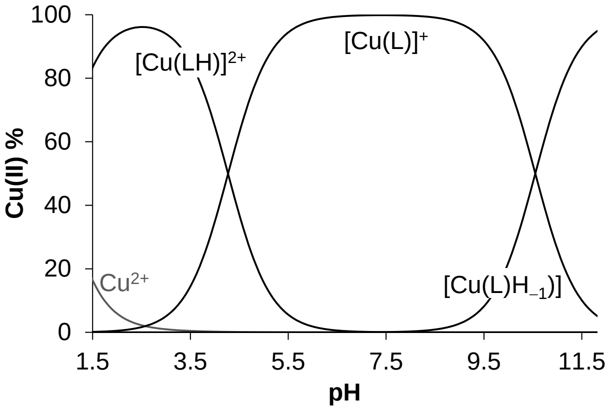

Figure S7 Concentration distribution curves calculated for the Cu(II) – VLX600 (1:1) system with the determined formation constants. {c_{VLX600} = 50 µM; $c_{Cu(II)}$ = 50 µM; *I* = 0.10 M KCl; *t* = 25.0 °C; 30% (*v*/*v*) DMSO/H_2O}7

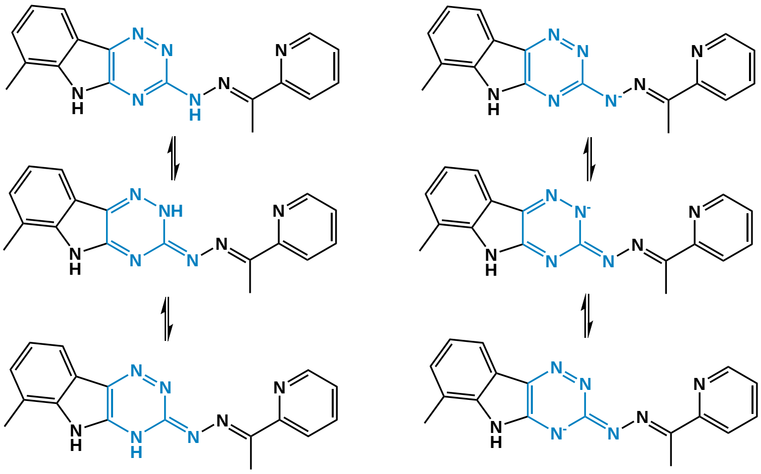

Scheme S1 The imine-enamine tautomeric forms of the neutral and monoanionic VLX600.

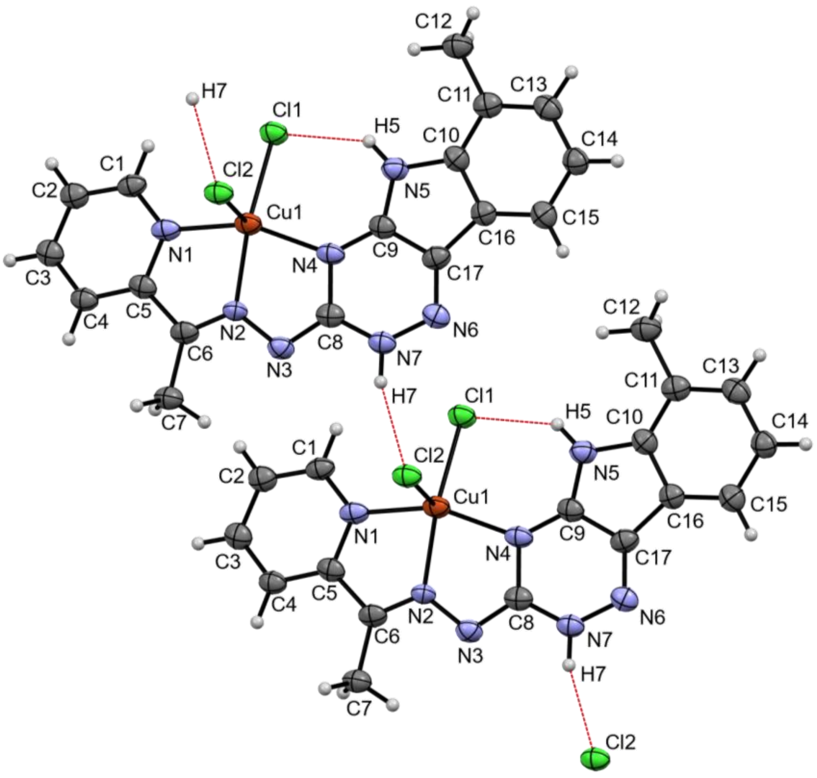

Figure S8 Hydrogen bonds of the [Cu(LH)Cl_2] crystal, highlighted in red. Intramolecular bond from Cl1 to N5 and intermolecular bond between Cl2 and N7.

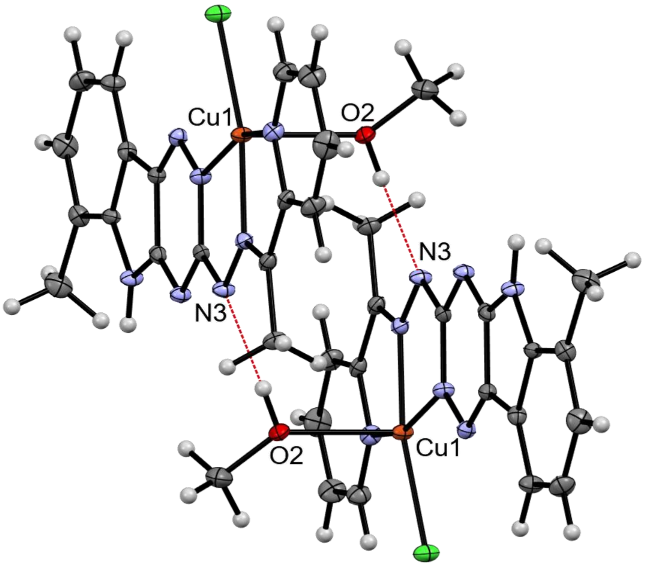

Figure S9 Intramolecular hydrogen bonds of [Cu(L)ClMeOH], highlighted in red between N3 and O2.

DFT calculations for the Cu(II) complexes of VLX600

Table S8 Selected bond lengths, angles and the relative energy values obtained from DFT calculations. Selected parameters from X-ray studies are also shown.

	**[Cu(LH)Cl_2]**		**X-ray**	**[Cu(L)Cl(MeOH]**		**X-ray**
			Bond lengths (Å)			
Cu - $_{Npy}$	2.029	2.042	1.995	2.031	2.042	2.004
Cu - $_{Nimine}$	1.996	1.993	1.961	1.990	1.988	1.959
Cu - N^2		2.038			2.010	1.966
Cu - N^4	2.050		1.997	2.023		
			Angles (˚)			
N_{py} - Cu- N_{imine}	79.8	79.4	80.38	80.4	80.2	80.12
N_{imine} - Cu- N^2		78.0			78.2	78.45
N_{imine} - Cu- N^4	77.5		77.73	77.9		
N_{py} - Cu- N^2		156.8			158.4	157.12
N_{py} - Cu- N^4	155.8		155.04	157.5		
			ΔG_{rel} (kJ/mol)			
**[Cu(LH)Cl_2]** (N^4) → **[Cu(LH))Cl_2]** (N^2)	40.3					
**[Cu(L)Cl(MeOH]** (N^4) → **[Cu(L)Cl(MeOH]** (N^2)	-2.5					

Table S9 Cartesian coordinates and energy values of [Cu(LH)Cl_2] (N^2).

Sum of electronic and thermal Energies (Eh)	-3600.4706
Sum of electronic and enthalpy Energies (Eh)	-3600.4697
Sum of electronic and thermal Free Energies (Eh)	-3600.5487
Number of Imaginary Frequencies	0

Molecular Geometry in Cartesian Coordinates

```
Cu       8.27389      6.21547     10.55931
Cl       9.48013      7.52056      8.68854
Cl       9.49906      4.26752     10.79538
N        6.84690      4.56567      8.46543
C       10.01072      8.98183     14.07043
H       10.36402      9.65501     14.84209
C        5.72216      1.55242      5.09244
H        6.26881      0.72567      4.65472
C       10.81271      7.94197     13.61639
H       11.80413      7.77422     14.01623
N        5.64400      7.39899     10.26319
N        6.79831      7.49250     10.96542
N        4.59778      6.16074      8.62163
C       10.31507      7.10162     12.62298
H       10.89217      6.27189     12.23470
N        9.10229      7.26570     12.10185
C        8.30903      8.27767     12.53118
C        4.27449      3.63001      6.15227
C        3.66818      2.89733      5.13821
C        6.30809      2.30277      6.10865
H        7.30292      2.07937      6.47325
C        5.57046      3.35745      6.64383
C        8.74112      9.15421     13.52247
```

H	8.10047	9.95750	13.85950
C	6.98602	8.37671	11.88670
C	4.43567	1.84528	4.62218
H	4.01071	1.24030	3.82883
C	2.28733	3.21945	4.63605
H	1.55158	3.16851	5.44501
H	1.98798	2.51805	3.85699
H	2.24577	4.23160	4.22098
N	3.74432	4.74301	6.85853
H	2.83263	5.15661	6.71298
N	6.79189	5.55520	9.32568
C	4.64230	5.15908	7.74373
C	5.81940	4.34451	7.67749
C	5.98985	9.40998	12.29417
H	6.40534	10.41140	12.14808
H	5.75251	9.30569	13.35684
H	5.07783	9.31174	11.71108
C	5.69781	6.38845	9.42765
H	3.79427	6.77669	8.70599

Table S10 Cartesian coordinates and energy values of [Cu(LH)Cl_2] (N^4).

Sum of electronic and thermal Energies (Eh)	-3600.4863
Sum of electronic and enthalpy Energies (Eh)	-3600.4853
Sum of electronic and thermal Free Energies (Eh)	-3600.5641
Number of Imaginary Frequencies	0

Molecular Geometry in Cartesian Coordinates

Cu	8.29715	6.55847	10.55931
Cl	9.30820	8.01204	8.71706
Cl	9.63235	4.65267	10.47428
N	4.40498	5.59878	7.83216
C	10.08706	9.04563	14.23256
H	10.45119	9.65414	15.05146
C	5.51203	2.01730	5.08830
H	4.95857	1.50652	4.30958
C	10.88143	8.04416	13.68735
H	11.87664	7.84295	14.06130
N	5.63171	7.71161	10.43210
N	6.80691	7.77092	11.10203
N	6.74754	5.91214	9.38228
C	10.37153	7.28767	12.63495
H	10.94433	6.49350	12.17300
N	9.15146	7.49309	12.14525
C	8.36251	8.46546	12.66686
C	6.98284	3.26538	7.05125
C	7.58661	2.20108	6.39251
C	4.92654	3.08551	5.76346
H	3.92403	3.42057	5.52786
C	5.67558	3.71291	6.75648
C	8.81084	9.26177	13.71693
H	8.17479	10.03508	14.12524
C	7.02473	8.60379	12.06427
C	6.80855	1.59022	5.39953
H	7.23500	0.75475	4.85506
C	8.97890	1.74572	6.73284
H	9.70138	2.55730	6.60135

H	9.27892	0.91256	6.09682
H	9.04139	1.42116	7.77642
N	7.52850	4.06555	8.08419
H	8.43343	3.95532	8.53610
N	4.54562	6.53200	8.77541
H	3.74650	7.13837	8.90996
C	6.63847	4.98546	8.45863
C	5.42902	4.82379	7.65563
C	6.04151	9.61242	12.55657
H	6.46759	10.61763	12.49003
H	5.80417	9.42607	13.60818
H	5.12697	9.57039	11.97062
C	5.65955	6.73543	9.55225

Table S11 Cartesian coordinates and energy values of [Cu(L)ClMeOH] (N^2).

Sum of electronic and thermal Energies (Eh)	-3255.3509
Sum of electronic and enthalpy Energies (Eh)	-3255.3499
Sum of electronic and thermal Free Energies (Eh)	-3255.4317
Number of Imaginary Frequencies	0

Molecular Geometry in Cartesian Coordinates

Cu	8.24856	6.06223	10.77448
N	6.90111	4.48639	8.61708
C	10.12434	8.91571	14.13959
H	10.50585	9.60903	14.87975
C	5.74064	1.48705	5.24572
H	6.24919	0.61603	4.84946
C	10.86672	7.79969	13.76690
H	11.83667	7.59225	14.19926
N	5.77368	7.45013	10.27687
N	6.87954	7.48147	11.02590
N	4.71251	6.26649	8.60302
C	10.33210	6.93797	12.81353
H	10.85941	6.05022	12.48718
N	9.14344	7.14983	12.25247
C	8.40908	8.23835	12.59816
C	4.38529	3.69089	6.20201
C	3.78493	2.97048	5.17033
C	6.32468	2.21973	6.27478
H	7.28390	1.93755	6.69179
C	5.63856	3.33461	6.75704
C	8.88298	9.13987	13.55191
H	8.29256	10.00329	13.82603
C	7.12623	8.40335	11.90496
C	4.49939	1.85902	4.71018
H	4.07081	1.26806	3.90770
C	2.45391	3.37480	4.59649
H	1.67496	3.37579	5.36622
H	2.14959	2.68938	3.80471
H	2.49492	4.38558	4.17721
N	3.90211	4.84103	6.85173
H	3.02858	5.30648	6.65466
N	6.84079	5.51273	9.44899
C	4.79165	5.23856	7.79444
C	5.90519	4.32288	7.78186
C	6.21764	9.55232	12.20096

H	6.72820	10.50238	12.01563
H	5.91724	9.54281	13.25327
H	5.32790	9.50097	11.57817
C	5.77718	6.40002	9.43639
O	9.41423	7.24114	9.08356
H	8.96583	7.03738	8.25372
Cl	9.53644	4.15702	10.92176
C	9.52358	8.66954	9.20312
H	8.53817	9.13809	9.27594
H	10.08230	8.86690	10.11687
H	10.06743	9.08772	8.35252

Table S12 Cartesian coordinates and energy values of [Cu(L)ClMeOH] (N^4).

Sum of electronic and thermal Energies (Eh)	-3255.3488
Sum of electronic and enthalpy Energies (Eh)	-3255.3479
Sum of electronic and thermal Free Energies (Eh)	-3255.4308
Number of Imaginary Frequencies	0

Molecular Geometry in Cartesian Coordinates

Cu	8.29419	6.49496	10.63064
N	4.49728	5.66682	7.83365
C	10.11291	9.00889	14.27308
H	10.47649	9.61879	15.09149
C	5.55936	2.02912	5.10518
H	5.00234	1.53260	4.31912
C	10.89353	7.97827	13.75891
H	11.87522	7.75635	14.15593
N	5.69590	7.73446	10.41941
N	6.84398	7.77535	11.09791
N	6.79628	5.90857	9.40315
C	10.38273	7.22241	12.70807
H	10.94223	6.40627	12.26815
N	9.17809	7.45245	12.18909
C	8.40199	8.45538	12.67756
C	7.03611	3.24068	7.08986
C	7.61787	2.15651	6.43613
C	4.99461	3.11258	5.77161
H	4.00420	3.47212	5.51956
C	5.74271	3.72647	6.77801
C	8.85557	9.25290	13.72926
H	8.23343	10.04869	14.11514
C	7.08839	8.62559	12.04939
C	6.84125	1.56493	5.43379
H	7.25184	0.71702	4.89595
C	8.99324	1.66508	6.79750
H	9.74372	2.44926	6.65381
H	9.27239	0.80833	6.18303
H	9.04012	1.36241	7.84874
N	7.58299	4.02295	8.12459
H	8.48583	3.90692	8.57332
N	4.57589	6.61875	8.74944
C	6.69342	4.97096	8.47971
C	5.51673	4.84303	7.67347
C	6.12817	9.67758	12.50170
H	6.60392	10.66215	12.49734
H	5.79585	9.47837	13.52589

H	5.25886	9.69857	11.84873
C	5.69777	6.73916	9.51443
O	9.19136	8.00776	9.07343
H	9.20029	8.89192	9.45988
C	8.60155	8.08734	7.76432
H	9.16668	8.77264	7.12782
H	8.64368	7.08570	7.33869
H	7.55857	8.41168	7.81871
Cl	9.80517	4.76910	10.33867

Table S13 Cartesian coordinates and energy values of $[Cu(HL)(H_2O)_2]^{2+}$ (N^2).

Sum of electronic and thermal Energies (Eh)	-2832.5769
Sum of electronic and enthalpy Energies (Eh)	-2832.5759
Sum of electronic and thermal Free Energies (Eh)	-2832.6578
Number of Imaginary Frequencies	0

Molecular Geometry in Cartesian Coordinates

Cu	8.20560	6.37230	10.58256
N	6.86616	4.69830	8.49678
C	9.93780	8.97143	14.17831
H	10.28315	9.59984	14.99016
C	5.80600	1.61836	5.16800
H	6.37207	0.80162	4.73641
C	10.75027	7.96676	13.66868
H	11.74070	7.78336	14.06362
N	5.57350	7.48296	10.32108
N	6.72555	7.59234	11.02401
N	4.56341	6.22426	8.66482
C	10.26443	7.18300	12.62380
H	10.85027	6.38327	12.18791
N	9.05143	7.37200	12.11137
C	8.24395	8.34758	12.59839
C	4.30764	3.66643	6.21409
C	3.71629	2.90872	5.21029
C	6.37654	2.39468	6.17350
H	7.37838	2.20146	6.53607
C	5.61259	3.43395	6.70078
C	8.66685	9.16748	13.63808
H	8.01861	9.94315	14.02201
C	6.91295	8.45558	11.96525
C	4.51023	1.87233	4.70139
H	4.09806	1.24785	3.91653
C	2.32586	3.18864	4.70959
H	1.59583	3.13927	5.52364
H	2.03890	2.46329	3.94808
H	2.26006	4.18988	4.27203
N	3.74975	4.77117	6.91502
H	2.82473	5.15617	6.77213
N	6.77902	5.69766	9.34931
C	4.63658	5.21887	7.79238
C	5.84061	4.43635	7.72393
C	5.90844	9.46236	12.41022
H	6.32382	10.47033	12.32201
H	5.65863	9.30178	13.46320
H	5.00405	9.39091	11.81137
C	5.65562	6.48737	9.46823

H	3.73734	6.80955	8.75381
O	9.53941	7.10471	8.89997
H	10.16328	7.80421	9.13451
H	9.04535	7.44316	8.14189
O	9.55142	4.82847	10.50727
H	9.92075	4.77879	9.61370
H	9.19789	3.95031	10.70730

Table S14 Cartesian coordinates and energy values of $[Cu(HL)(H_2O)_2]^{2+}$ (N^4).

Sum of electronic and thermal Energies (Eh)	-2832.5861
Sum of electronic and enthalpy Energies (Eh)	-2832.5852
Sum of electronic and thermal Free Energies (Eh)	-2832.6658
Number of Imaginary Frequencies	0

Molecular Geometry in Cartesian Coordinates

Cu	8.23715	6.57780	10.69422
N	4.36041	5.53371	7.96730
C	10.05972	9.21612	14.22156
H	10.42813	9.85571	15.01430
C	5.49383	2.08751	5.08656
H	4.92793	1.57653	4.31703
C	10.86375	8.21628	13.68926
H	11.87048	8.04695	14.04743
N	5.57340	7.65684	10.54983
N	6.75326	7.77271	11.19669
N	6.74758	5.91884	9.44401
C	10.34886	7.41788	12.67007
H	10.92601	6.61953	12.22184
N	9.11524	7.58882	12.20203
C	8.31524	8.55764	12.71288
C	6.99696	3.33879	7.02063
C	7.61581	2.32109	6.30516
C	4.89548	3.10980	5.81865
H	3.87080	3.40951	5.63755
C	5.66282	3.73812	6.79690
C	8.76696	9.39153	13.72939
H	8.12339	10.16216	14.13092
C	6.96389	8.64052	12.13104
C	6.81931	1.70743	5.32810
H	7.25539	0.90711	4.74038
C	9.03851	1.91110	6.56813
H	9.72249	2.75568	6.43828
H	9.34189	1.11646	5.88631
H	9.15902	1.54823	7.59390
N	7.55007	4.13307	8.05839
H	8.49158	4.04785	8.41295
N	4.49384	6.45071	8.92312
H	3.67478	7.01847	9.10223
C	6.64398	5.00725	8.49830
C	5.41058	4.81109	7.73677
C	5.95869	9.63144	12.60963
H	6.35068	10.64631	12.49562
H	5.75538	9.47569	13.67319
H	5.03224	9.53810	12.04921
C	5.62437	6.69096	9.66198
O	9.60087	7.28430	9.05826

H	10.10007	8.08924	9.25012
H	9.17061	7.44437	8.20786
O	9.64255	5.07638	10.73131
H	9.37701	4.15840	10.87928
H	10.21790	5.09226	9.95311

Table S15 Cartesian coordinates and energy values of $[Cu(L)(H_2O)_2]^+$ (N^2).

Sum of electronic and thermal Energies (Eh)	-2832.1509
Sum of electronic and enthalpy Energies (Eh)	-2832.1500
Sum of electronic and thermal Free Energies (Eh)	-2832.2320
Number of Imaginary Frequencies	0

Molecular Geometry in Cartesian Coordinates

Cu	8.18148	6.37776	10.56318
N	6.89045	4.70143	8.49912
C	9.92647	8.94528	14.17529
H	10.27178	9.56796	14.99195
C	5.80985	1.60377	5.19171
H	6.36993	0.78052	4.76415
C	10.74073	7.94130	13.66090
H	11.73009	7.75368	14.05643
N	5.56143	7.48933	10.31456
N	6.69611	7.59172	11.01191
N	4.53547	6.25534	8.64613
C	10.25296	7.16800	12.61145
H	10.83730	6.36976	12.17023
N	9.04018	7.36132	12.09691
C	8.22963	8.33474	12.58722
C	4.31980	3.67812	6.23298
C	3.72948	2.90870	5.23142
C	6.38305	2.38490	6.19056
H	7.38469	2.18783	6.55324
C	5.62856	3.43449	6.71528
C	8.65867	9.14733	13.63606
H	8.01189	9.92181	14.02493
C	6.90550	8.45289	11.95984
C	4.51307	1.86512	4.72700
H	4.09452	1.23857	3.94660
C	2.33868	3.19376	4.73268
H	1.60779	3.14042	5.54627
H	2.04831	2.47511	3.96557
H	2.27179	4.19879	4.30309
N	3.77166	4.77785	6.91886
H	2.84937	5.16240	6.77648
N	6.77462	5.71508	9.34146
C	4.66897	5.24801	7.81755
C	5.86273	4.43971	7.73108
C	5.90737	9.46923	12.40988
H	6.33298	10.47534	12.35365
H	5.62289	9.29204	13.45197
H	5.01626	9.42383	11.78818
C	5.62383	6.48016	9.42481

O	9.56492	7.12728	8.90543
H	10.17483	7.83345	9.15576
H	9.07531	7.46911	8.14609
O	9.54425	4.82235	10.47037
H	9.92298	4.82674	9.57955
H	9.16981	3.94036	10.60191

Table S16 Cartesian coordinates and energy values of $[Cu(L)(H_2O)_2]^+$ (N^4).

Sum of electronic and thermal Energies (Eh)	-2832.1454
Sum of electronic and enthalpy Energies (Eh)	-2832.1444
Sum of electronic and thermal Free Energies (Eh)	-2832.2232
Number of Imaginary Frequencies	0

Molecular Geometry in Cartesian Coordinates

Cu	8.19739	6.56627	10.68981
N	4.36872	5.56479	7.99963
C	10.06797	9.20485	14.19236
H	10.44490	9.84581	14.98034
C	5.51496	2.10393	5.09714
H	4.94780	1.59614	4.32572
C	10.86774	8.20034	13.65644
H	11.87698	8.03000	14.00682
N	5.55151	7.66032	10.56093
N	6.71778	7.77026	11.19510
N	6.72792	5.92593	9.45974
C	10.34021	7.40284	12.64534
H	10.91083	6.60105	12.19438
N	9.10246	7.57408	12.18513
C	8.30546	8.54688	12.69796
C	7.01964	3.34867	7.03794
C	7.63536	2.33034	6.31363
C	4.91861	3.12325	5.83302
H	3.89342	3.42201	5.65069
C	5.68233	3.75340	6.81747
C	8.77381	9.38253	13.71175
H	8.13628	10.15631	14.11685
C	6.95676	8.63905	12.13230
C	6.84250	1.72008	5.33541
H	7.27769	0.92159	4.74406
C	9.05915	1.92177	6.57692
H	9.74364	2.76619	6.44544
H	9.36454	1.12546	5.89734
H	9.18255	1.56186	7.60377
N	7.56835	4.13324	8.07289
H	8.50854	4.05374	8.42642
N	4.43032	6.49685	8.93373
C	6.64131	5.01044	8.50984
C	5.43436	4.82280	7.76141
C	5.96167	9.64110	12.61906
H	6.35182	10.65639	12.49983
H	5.75673	9.49179	13.68364
H	5.03191	9.54983	12.06293
C	5.57986	6.67987	9.64211
O	9.59048	7.25691	9.04772

H	10.08650	8.06486	9.23417
H	9.15303	7.41386	8.20060
O	9.60818	5.04404	10.73312
H	9.34238	4.12350	10.86056
H	10.19978	5.07261	9.96796

Table S17 Calculated copper(II) hyperfine couplings ($A_{||}$) of the complexes.

	$[Cu(HL)(H_2O)_2]^{2+}$ (N^2)	$[Cu(HL)(H_2O)_2]^{2+}$ (N^4)	$[Cu(L)(H_2O)_2]^{+}$ (N^2)	$[Cu(L)(H_2O)_2]^{+}$ (N^4)
$A_{||}$ (MHz)	554	548	547	546
	$[Cu(HL)_2]^{2+}$ (N^2)	$[Cu(HL)_2]^{2+}$ (N^4)	$[Cu(HL)DMSO(Cl)]^{+}$ (N^4)	$[Cu(HL)(DMSO)_2]^{2+}$ (N^4)
$A_{||}$ (MHz)	510	498	487	523

Table S18 Cartesian coordinates and energy values of $[Cu(HL)_2]^{2+}$ (N^2).

Sum of electronic and thermal Energies (Eh)	-3720.503075
Sum of electronic and enthalpy Energies (Eh)	-3720.502131
Sum of electronic and thermal Free Energies (Eh)	-3720.624575
Number of Imaginary Frequencies	0

Molecular Geometry in Cartesian Coordinates

N	-2.454594	0.301211	0.364223
C	4.211444	-4.096702	-1.670518
H	5.031713	-4.640144	-2.122080
C	-6.172478	2.719166	1.756240
H	-6.624607	2.843634	2.732383
C	3.976330	-4.175674	-0.304441
H	4.602140	-4.773618	0.344244
N	-0.518531	-0.483596	-2.507364
N	0.402500	-1.283178	-1.936511
N	-2.393699	0.847561	-2.409891
C	2.901375	-3.460418	0.219826
H	2.673165	-3.490031	1.279605
N	2.104025	-2.709399	-0.533522
C	2.327282	-2.620435	-1.859639
C	-5.083075	2.455549	-0.761185
C	-6.216987	3.257604	-0.639958
C	-5.042552	1.923355	1.614445
H	-4.596963	1.418968	2.462322
C	-4.492564	1.791242	0.339458
C	3.379352	-3.309879	-2.463086
H	3.552175	-3.250806	-3.527152
C	1.388374	-1.760822	-2.620502
C	-6.741390	3.367867	0.650547
H	-7.623329	3.981220	0.796488
C	-6.826190	3.950021	-1.828180

H	-6.110192	4.626854	-2.304527
H	-7.695821	4.535664	-1.530246
H	-7.148010	3.228599	-2.585721
N	-4.345559	2.164993	-1.925151
H	-4.544373	2.508621	-2.853155
N	-1.511231	-0.210984	-0.435197
C	-3.315075	1.347991	-1.612717
C	-3.355289	1.075407	-0.196098
C	1.565201	-1.454101	-4.073670
H	2.491676	-1.850712	-4.474705
H	1.576520	-0.370333	-4.226751
H	0.738189	-1.868980	-4.658536
C	-1.520394	0.073915	-1.743847
H	-0.497155	-0.259425	-3.494038
N	2.017127	0.884636	-0.181417
C	-2.398600	-5.382933	0.947994
H	-3.003702	-6.248998	1.182801
C	4.639340	4.380072	-1.744049
H	4.926628	4.671503	-2.746559
C	-2.085112	-5.076004	-0.368991
H	-2.432889	-5.686708	-1.190555
N	0.737943	-0.596430	2.780448
N	0.110711	-1.593072	2.133901
N	2.085006	1.284863	2.621909
C	-1.303469	-3.952594	-0.622898
H	-1.031276	-3.668650	-1.631204
N	-0.852705	-3.169646	0.354988
C	-1.151616	-3.457991	1.646658
C	3.955216	3.708434	0.838693
C	4.771694	4.824874	0.669335
C	3.827495	3.269111	-1.553556
H	3.469708	2.679768	-2.387918
C	3.483475	2.932035	-0.244929
C	-1.927183	-4.564078	1.972267
H	-2.160696	-4.786195	3.003625
C	-0.602737	-2.527412	2.660173
C	5.096665	5.134948	-0.654381
H	5.729391	5.995372	-0.839785
C	5.265671	5.631397	1.838746
H	4.433505	6.039650	2.420201
H	5.880581	6.464952	1.499945
H	5.869738	5.019393	2.515546
N	3.468858	3.161331	2.044717
H	3.649853	3.518478	2.971760
N	1.379192	0.080133	0.670172
C	2.715297	2.078035	1.776756
C	2.685849	1.880291	0.343453
C	-0.882088	-2.706920	4.113734
H	-0.474756	-3.660294	4.459356
H	-1.960335	-2.720593	4.287838
H	-0.452820	-1.915045	4.723714
C	1.432444	0.298589	2.002176
H	0.741407	-0.501404	3.788230
Cu	0.288035	-1.514253	0.146504

Table S19 Cartesian coordinates and energy values of $[Cu(HL)_2]^{2+}$ (N^2).

Sum of electronic and thermal Energies (Eh) -3720.49566

Sum of electronic and enthalpy Energies (Eh) -3720.494716

Sum of electronic and thermal Free Energies (Eh) -3720.613592

Number of Imaginary Frequencies 0

Molecular Geometry in Cartesian Coordinates

N	2.399029	-1.547930	3.417884
C	-3.128149	4.456648	-0.857816
H	-3.892525	5.205252	-1.020510
C	4.838394	-4.805510	1.168541
H	5.480228	-5.599524	1.529272
C	-2.493961	3.849328	-1.932761
H	-2.741312	4.104232	-2.953812
N	0.127993	1.119221	2.929410
N	-0.471231	1.725688	1.892108
N	1.193734	-0.144010	1.309017
C	-1.518660	2.890182	-1.676130
H	-0.994255	2.391298	-2.480268
N	-1.179193	2.542949	-0.436049
C	-1.790482	3.129716	0.624176
C	3.227209	-2.815840	0.171765
C	3.818930	-3.668991	-0.755133
C	4.232112	-3.944256	2.075271
H	4.383943	-4.048167	3.141743
C	3.414065	-2.935627	1.566641
C	-2.772151	4.095295	0.440090
H	-3.255155	4.555792	1.289888
C	-1.340592	2.677657	1.958123
C	4.632300	-4.667808	-0.211770
H	5.119580	-5.360226	-0.888647
C	3.587462	-3.512567	-2.233097
H	2.523558	-3.582041	-2.479897
H	4.112942	-4.287717	-2.790399
H	3.943698	-2.540847	-2.588721
N	2.365849	-1.726345	-0.086504
H	2.019965	-1.455938	-0.999898
N	1.559073	-0.530751	3.660860
C	1.995797	-1.172901	1.078534
C	2.616921	-1.885412	2.166192
C	-1.873817	3.288282	3.208649
H	-2.937381	3.058235	3.312708
H	-1.767531	4.374052	3.169734
H	-1.360723	2.934104	4.099888
C	1.004062	0.101569	2.632624
H	-0.096446	1.322422	3.894999
N	-2.063522	-2.477400	-2.699594
C	3.597415	4.729496	-0.898365
H	4.268554	5.514064	-1.223410
C	-6.055067	-3.707002	-0.649985
H	-6.825306	-4.410392	-0.940925
C	2.989937	4.786820	0.348102
H	3.172673	5.606642	1.029504
N	0.769754	-0.382367	-2.364652
N	1.119657	0.765384	-1.704331
N	-1.037714	-0.575422	-0.904588
C	2.124142	3.755398	0.709046
H	1.622695	3.761718	1.670139
N	1.867237	2.721805	-0.086829
C	2.462048	2.653576	-1.293772
C	-4.144437	-1.900414	0.161960
C	-5.257236	-2.057461	0.985800

C	-4.938369	-3.533473	-1.457785
H	-4.816213	-4.087394	-2.379605
C	-3.970933	-2.616990	-1.045030
C	3.334720	3.646988	-1.734912
H	3.793181	3.597876	-2.711164
C	2.119699	1.472073	-2.121224
C	-6.205535	-2.982781	0.541913
H	-7.091088	-3.142262	1.146608
C	-5.413657	-1.275244	2.260950
H	-4.600770	-1.490487	2.961325
H	-6.354669	-1.521896	2.752296
H	-5.404988	-0.197659	2.069683
N	-3.040013	-1.043878	0.351493
H	-2.906537	-0.419683	1.132972
N	-0.902019	-1.850402	-2.942593
C	-2.186943	-1.187207	-0.680573
C	-2.712629	-2.163076	-1.597927
C	2.882982	1.119391	-3.357477
H	3.204672	0.074877	-3.320312
H	3.771059	1.730239	-3.478277
H	2.256076	1.248170	-4.246063
C	-0.464700	-0.957654	-2.056212
H	1.054400	-0.497365	-3.331812
Cu	0.196275	1.165986	0.105279

Table S20 Cartesian coordinates and energy values of $[Cu(HL)DMSO(Cl)]^{+}$ (N^{4}).

Sum of electronic and thermal Energies (Eh)	-3693.2015
Sum of electronic and enthalpy Energies (Eh)	-3693.2005
Sum of electronic and thermal Free Energies (Eh)	-3693.2882
Number of Imaginary Frequencies	0

Molecular Geometry in Cartesian Coordinates

Cu	8.60287	6.88880	10.16484
Cl	9.09475	8.16123	8.12596
N	4.50988	4.86149	8.65670
C	10.81572	9.67535	13.32094
H	11.27131	10.32335	14.05981
C	5.70517	1.94637	5.22923
H	5.11482	1.29894	4.59221
C	11.59287	9.06012	12.34617
H	12.66339	9.20849	12.29667
N	5.84899	7.12786	11.08282
N	7.11763	7.53642	11.33873
N	7.00745	5.89019	9.44666
C	10.96431	8.23415	11.41918
H	11.51918	7.72814	10.63896
N	9.65181	8.01640	11.44752
C	8.87530	8.61664	12.38443
C	7.27322	3.56673	6.80630
C	7.92719	2.78181	5.86504
C	5.07179	2.73879	6.18383
H	3.99605	2.72196	6.30701
C	5.86990	3.55610	6.98090
C	9.43977	9.45586	13.34076
H	8.81653	9.93075	14.08589
C	7.42987	8.32516	12.31147

C	7.09689	1.96993	5.07957
H	7.55895	1.33753	4.32931
C	9.42327	2.80890	5.71242
H	9.77323	3.81196	5.44878
H	9.74404	2.11763	4.93279
H	9.91843	2.52730	6.64726
N	7.85133	4.47518	7.72741
H	8.83877	4.65308	7.88306
N	4.66236	5.74632	9.64715
H	3.80821	6.02634	10.11284
C	6.89237	5.02149	8.47356
C	5.59981	4.48768	8.06090
C	6.46045	8.90408	13.28615
H	6.50941	9.99666	13.26315
H	6.71198	8.58578	14.30224
H	5.44832	8.58477	13.05057
C	5.85180	6.27696	10.07966
S	10.26295	4.10574	10.62071
O	9.90078	5.32749	9.71043
C	8.69530	3.49889	11.30065
H	8.13331	3.05730	10.47930
H	8.93839	2.73776	12.04313
H	8.15680	4.33410	11.74977
C	10.96369	4.79740	12.13999
H	11.17633	3.95982	12.80583
H	11.88706	5.30452	11.86501
H	10.24788	5.48652	12.58750

Table S21 Cartesian coordinates and energy values of $[Cu(HL)(DMSO)_2]^{2+}$ (N^4).

Sum of electronic and thermal Energies (Eh)	-3785.9137
Sum of electronic and enthalpy Energies (Eh)	-3785.9128
Sum of electronic and thermal Free Energies (Eh)	-3786.0102
Number of Imaginary Frequencies	0

Molecular Geometry in Cartesian Coordinates

Cu	8.50830	6.78870	10.25047
N	4.38559	4.80983	8.69928
C	10.61921	9.92527	13.17328
H	11.03707	10.67536	13.83366
C	5.53610	1.96827	5.19406
H	4.93561	1.35654	4.53175
C	11.43733	9.20639	12.31089
H	12.50600	9.37025	12.27127
N	5.74123	7.10790	11.07745
N	7.00684	7.52092	11.33489
N	6.90592	5.76455	9.52867
C	10.85269	8.25534	11.47977
H	11.44877	7.67686	10.78824
N	9.54479	8.01107	11.48915
C	8.73098	8.71096	12.32004
C	7.12761	3.49281	6.83576
C	7.77088	2.73984	5.86244
C	4.91411	2.73472	6.17728
H	3.83771	2.73476	6.29550
C	5.72494	3.50595	7.00692
C	9.24817	9.67670	13.17853

H	8.59036	10.22994	13.83436
C	7.29144	8.39896	12.23694
C	6.92828	1.97403	5.04471
H	7.37962	1.36566	4.26852
C	9.26694	2.76215	5.70830
H	9.61857	3.76751	5.45468
H	9.58506	2.07850	4.92101
H	9.76277	2.47096	6.63985
N	7.72060	4.35229	7.79143
H	8.70756	4.54558	7.91393
N	4.55140	5.68664	9.69317
H	3.69981	5.99855	10.14317
C	6.77326	4.91181	8.54250
C	5.47114	4.41681	8.10875
C	6.29070	9.06929	13.11634
H	6.29074	10.14775	12.93038
H	6.55003	8.91755	14.16775
H	5.29523	8.67522	12.92894
C	5.74784	6.20583	10.12185
S	10.53263	4.25380	10.15232
O	9.86363	5.50088	9.46405
C	9.19337	3.39622	11.01938
H	8.52689	2.98189	10.26508
H	9.64949	2.59302	11.59972
H	8.67704	4.10893	11.66356
C	11.43795	4.87189	11.58896
H	11.83752	3.99817	12.10611
H	12.25283	5.48905	11.21533
H	10.76234	5.43499	12.23169
S	9.25985	8.57636	7.38332
O	8.38324	8.24108	8.61494
C	9.35477	7.06734	6.37751
H	8.33722	6.80717	6.08955
H	9.95141	7.30104	5.49435
H	9.81928	6.27804	6.96617
C	10.97853	8.58144	7.96276
H	11.62098	8.72185	7.09208
H	11.08656	9.41935	8.64978
H	11.18400	7.63200	8.45674

Cu(II) complexes of VLX600: EPR spectroscopy in DMF/H_2O solvent mixture

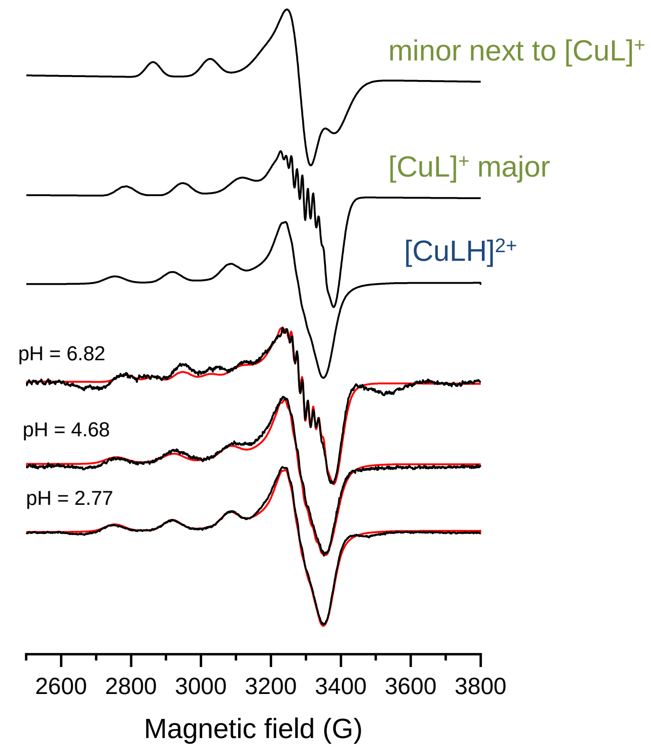

Figure S10 Frozen solution EPR spectra recorded for Cu(II) – VLX600 system in 30% DMF/H_2O solution (lower spectra) in addition to the calculated component EPR spectra (upper spectra) obtained by the simulation of the measured spectra (see EPR parameters in Table S21). {c_{VLX600} = 0.5 mM; $c_{Cu(II)}$ = 0.34 mM; I = 0.10 M KCl; T = 77 K}

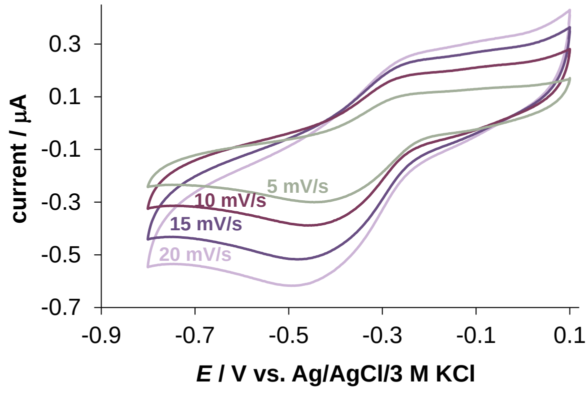

Figure S11 Cyclic voltammograms of the copper complexes of VLX600 at different scan rates using reference electrode: Ag/AgCl/3 M KCl and Pt working and counter electrodes. {c_{VLX600} = 1.0 mM, $c_{Cu(II)}$ = 1.0 mM; 60% (v/v) DMF/buffered aqueous solution at pH 7.4; t = 25 °C; I = 0.1 M (TBAN)}

Table S22 Anisotropic EPR parameters of the Cu(II) complexes of VLX600 determined by the simulation of frozen solution EPR spectra recorded at various pH values in 30% (*v*/*v*) DMF/H_2O (Figure S8).[a] The coupling values are in 10^{-4} cm^{-1} unit. {I = 0.1 M KCl; T = 77 K}

	$g_{\perp}$	$g_{\parallel}$	$A_{\perp}$ ($\times10^{-4}cm^{-1}$)	$A_{\parallel}$ (G) ($\times10^{-4}cm^{-1}$)	a^{N}_{0} (G) ($\times10^{-4}cm^{-1}$)	$g_{0,calc}$ [b]
**$[CuLH]^{2+}$**	2.048	2.247	17.4	168.9	16, 13, 13	2.114
**$[CuL]^{+}$**	2.041	2.225	27.6	166.2	16, 13, 13	2.102
**minor species next to $[CuL]^{+}$ [b]**	2.051	2.170	11	162.1		2.090

[a] The experimental error were ±0.002 for $g_{\perp}$ and ±0.001 for $g_{\parallel}$, ± 2 $\times10^{-4}cm^{-1}$ for $A_{\perp}$ and ±1 $\times10^{-4}cm^{-1}$ for $A_{\parallel}$. [b] Calculated by the equation $g_{0,calc} = (2g_{\perp}+g_{\parallel})/3$. [b] Identified as $[CuL_2]$.

DFT calculations for the $[Zn(LH)Cl_2]$ and $[Zn(LH)(H_2O)_2]^{2+}$ complexes

N_{py}, N_{imine}, N^2 coordination mode	N_{py}, N_{imine}, N^4 coordination mode

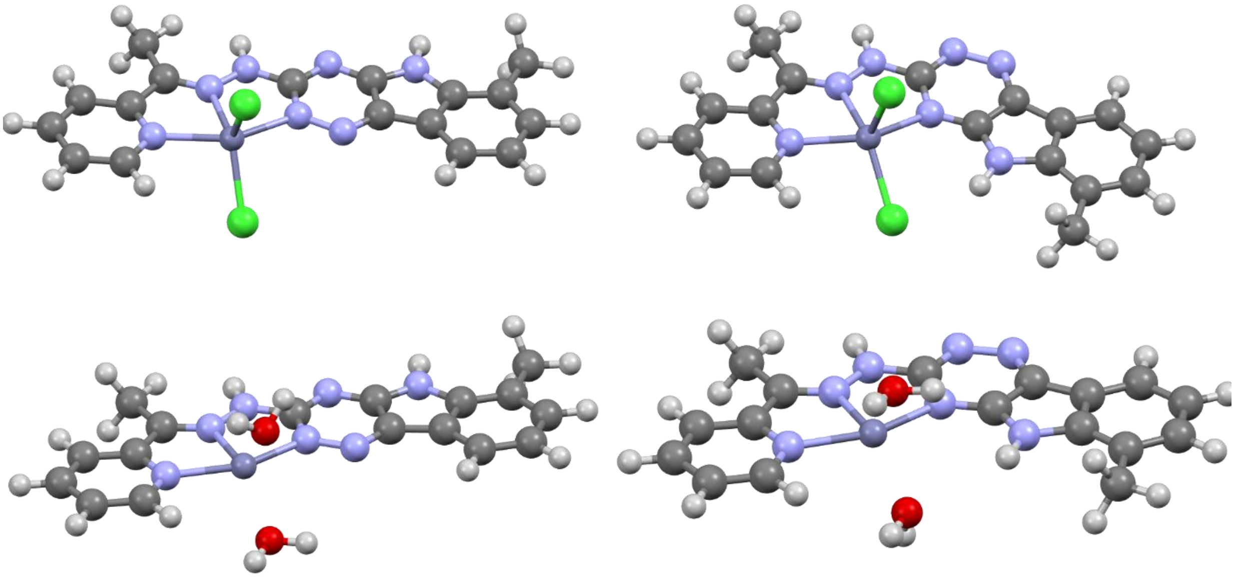

Figure S12 DFT optimized structures of the isomers formed in the $[Zn(LH)Cl_2]$ (top) and $[Zn(LH)(H_2O)_2]^{2+}$ (bottom) systems.

Table S23 Selected bond lengths, angles and the relative energy values obtained from DFT calculations. Selected parameters from X-ray studies are also shown.

	$[Zn(LH)Cl_2]$		$[Zn(LH)(H_2O)_2]^{2+}$		X-ray
Bond lengths (Å)					
Zn - $_{Npy}$	2.124	2.169	2.085	2.075	2.200
Zn - $_{Nimine}$	2.190	2.182	2.103	2.116	2.133
Zn - N^2		2.171		2.091	2.191
Zn - N^4	2.193		2.124	1.9929	
Angles (˚)					
N_{py} - Zn- N_{imine}	73.96	73.04	76.69	76.66	73.06
N_{imine} - Zn- N^2		73.29		75.88	72.56
N_{imine} - Zn- N^4	72.09		75.73		
N_{py} - Zn- N^2	144.43	146.16			143.32
N_{py} - Zn- N^4			152.28	152.52	
ΔG_{rel} (kJ/mol)					
**$[Zn(LH)Cl_2]$** (N^4) → **$[Zn(LH)Cl_2]$** (N^2)	5.23				
**$[Zn(LH)(H_2O)_2]^{2+}$** (N^4) → **$[Zn(LH)(H_2O)_2]^{2+}$** (N^2)	-5.48				

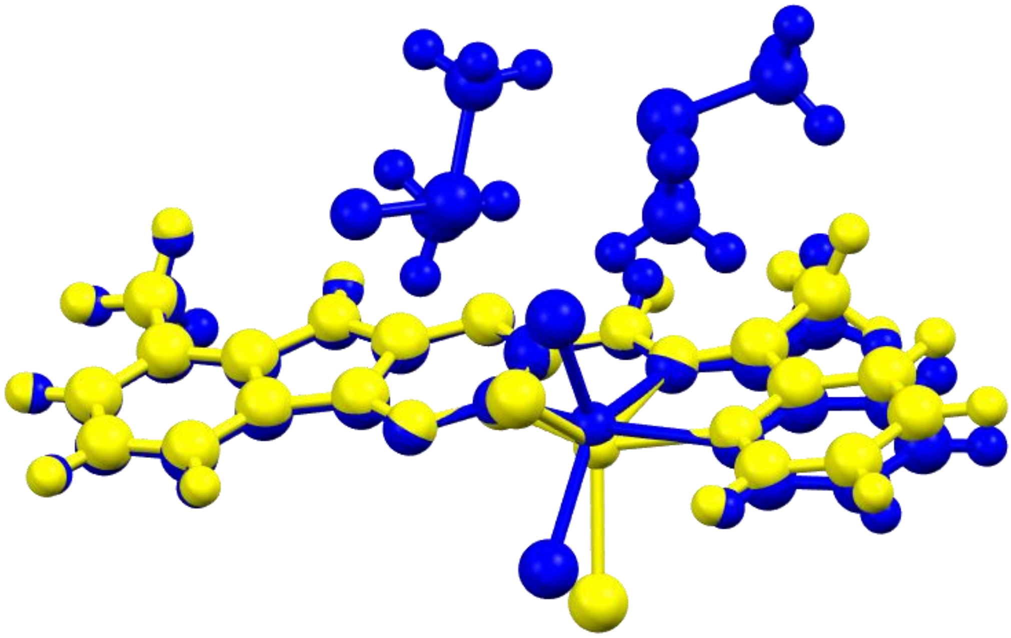

Figure S13 Comparison of the structures ([Zn(LH)Cl$_2$]) obtained from DFT calculation (yellow) and X-ray crystallography (blue).

Table S24 Cartesian coordinates and energy values of [Zn(LH)Cl$_2$] (N^2).

Sum of electronic and thermal Energies (Eh)	-3759.2266
Sum of electronic and enthalpy Energies (Eh)	-3759.2257
Sum of electronic and thermal Free Energies (Eh)	-3759.3005
Number of Imaginary Frequencies	0

Molecular Geometry in Cartesian Coordinates

Zn	8.542930000000	6.147060000000	10.437390000000
Cl	10.048770000000	6.919290000000	8.863520000000
Cl	9.051050000000	4.069340000000	11.302600000000
N	6.948860000000	4.595420000000	8.257620000000
C	9.909450000000	9.076790000000	14.148690000000
H	10.187290000000	9.743810000000	14.953990000000
C	5.662490000000	1.610140000000	4.964420000000
H	6.194220000000	0.798800000000	4.485800000000
C	10.794340000000	8.114980000000	13.695100000000
H	11.779570000000	7.999380000000	14.124460000000
N	5.774220000000	7.300250000000	10.209880000000
N	6.879530000000	7.467560000000	10.939520000000
N	4.625350000000	6.145380000000	8.589770000000
C	10.390370000000	7.282410000000	12.661540000000
H	11.040610000000	6.511680000000	12.267380000000
N	9.193490000000	7.380340000000	12.098050000000
C	8.326960000000	8.310280000000	12.531130000000
C	4.256610000000	3.641830000000	6.133860000000
C	3.604950000000	2.906430000000	5.165790000000

C	6.291820000000	2.364930000000	5.936570000000
H	7.312300000000	2.157480000000	6.227660000000
C	5.580560000000	3.394090000000	6.530500000000
C	8.658880000000	9.177270000000	13.559040000000
H	7.955810000000	9.921800000000	13.900890000000
C	7.014500000000	8.349880000000	11.856160000000
C	4.347250000000	1.881480000000	4.592060000000
H	3.881920000000	1.272460000000	3.826740000000
C	2.192310000000	3.194280000000	4.760620000000
H	1.513920000000	3.109050000000	5.613920000000
H	1.863880000000	2.497660000000	3.991030000000
H	2.096100000000	4.210610000000	4.369110000000
N	3.749770000000	4.724190000000	6.874720000000
H	2.820930000000	5.111550000000	6.799860000000
N	6.890440000000	5.588820000000	9.144770000000
C	4.695460000000	5.162960000000	7.721110000000
C	5.877890000000	4.363370000000	7.547310000000
C	5.992570000000	9.352690000000	12.265920000000
H	6.403120000000	10.360330000000	12.176380000000
H	5.717340000000	9.202600000000	13.312140000000
H	5.087120000000	9.305210000000	11.667160000000
C	5.763680000000	6.303360000000	9.275820000000
H	4.935380000000	7.849770000000	10.348020000000

Table S25 Cartesian coordinates and energy values of $[Zn(LH)Cl_2]$ (N^4).

Sum of electronic and thermal Energies (Eh)	-3759.2266
Sum of electronic and enthalpy Energies (Eh)	-3759.2257
Sum of electronic and thermal Free Energies (Eh)	-3759.3025
Number of Imaginary Frequencies	0

Molecular Geometry in Cartesian Coordinates

Zn	8.534160000000	6.529330000000	10.446890000000
Cl	9.593840000000	7.629970000000	8.713890000000
Cl	9.414200000000	4.412490000000	10.785290000000
N	4.416260000000	5.614920000000	7.841650000000
C	10.032570000000	9.146420000000	14.277260000000
H	10.350300000000	9.764390000000	15.106600000000
C	5.481200000000	2.047730000000	5.066770000000
H	4.916360000000	1.552460000000	4.288350000000
C	10.885850000000	8.200870000000	13.737880000000
H	11.884420000000	8.048340000000	14.122220000000
N	5.715070000000	7.635150000000	10.421850000000
N	6.846950000000	7.754330000000	11.117300000000
N	6.752950000000	5.888120000000	9.339520000000
C	10.432990000000	7.434890000000	12.674140000000
H	11.057530000000	6.682140000000	12.210220000000
N	9.216840000000	7.580400000000	12.161660000000
C	8.375330000000	8.491210000000	12.683980000000
C	6.963810000000	3.254840000000	7.017920000000
C	7.549450000000	2.200180000000	6.351080000000
C	4.917920000000	3.107630000000	5.752870000000
H	3.918310000000	3.450200000000	5.523170000000
C	5.667550000000	3.721300000000	6.743010000000
C	8.761730000000	9.294260000000	13.743850000000

H	8.083030000000	10.025910000000	14.155560000000
C	7.034670000000	8.584280000000	12.073050000000
C	6.769650000000	1.608130000000	5.365520000000
H	7.181730000000	0.775030000000	4.809540000000
C	8.934880000000	1.729300000000	6.669050000000
H	9.664680000000	2.529120000000	6.517180000000
H	9.210050000000	0.888360000000	6.034550000000
H	9.012280000000	1.413490000000	7.712840000000
N	7.526360000000	4.034270000000	8.043390000000
H	8.417420000000	3.892660000000	8.504900000000
N	4.517880000000	6.558420000000	8.780440000000
C	6.633660000000	4.958290000000	8.417750000000
C	5.441550000000	4.817030000000	7.644300000000
C	6.046090000000	9.573360000000	12.584620000000
H	6.438840000000	10.586310000000	12.471700000000
H	5.865840000000	9.408780000000	13.648670000000
H	5.091120000000	9.521870000000	12.069060000000
C	5.656960000000	6.652290000000	9.472130000000
H	4.904750000000	8.220520000000	10.579400000000

Table S26 Cartesian coordinates and energy values of $[Zn(LH)(H_2O)_2]^{2+}$ (N^2).

Sum of electronic and thermal Energies (Eh)	-2988.6090
Sum of electronic and enthalpy Energies (Eh)	-2988.6080
Sum of electronic and thermal Free Energies (Eh)	-2988.6849
Number of Imaginary Frequencies	0

Molecular Geometry in Cartesian Coordinates

Zn	-1.673382203911	-0.765271476098	-0.021106351251
N	1.375580505798	-0.831424088200	-0.030965155291
C	-6.420419025976	0.104518005029	-0.067641002077
H	-7.475266605431	0.344571321911	-0.072987670255
C	5.819923933871	-2.101396495081	-0.043629129750
H	6.286736988579	-3.077008105506	-0.053258378931
C	-5.999814512857	-1.211972987577	-0.076790721271
H	-6.698571327844	-2.036168841573	-0.089469300139
N	-0.742518390706	2.015352152174	-0.004856335822
N	-1.891005289394	1.339403574195	-0.018194769271
N	1.559292042107	1.980604898224	-0.001986014166
C	-4.637835390903	-1.469264171042	-0.069715384983
H	-4.253418934481	-2.481324677905	-0.075068965464
N	-3.730707571815	-0.496488119961	-0.053551265987
C	-4.130685575694	0.792175896274	-0.043923314730
C	4.710826513000	0.396731980208	-0.017943950749
C	6.087492159695	0.324258611435	-0.018132093112
C	4.441316088440	-1.999097879618	-0.043581054113
H	3.817703204965	-2.882335201546	-0.053381936168
C	3.879356948448	-0.733769227189	-0.030425501259
C	-5.473905210069	1.118277309016	-0.051507822517
H	-5.786846509593	2.151323033547	-0.044162247507
C	-3.071662920056	1.828017229525	-0.026002121089
C	6.618649669168	-0.959466087947	-0.031101471114
H	7.695698999158	-1.073000177386	-0.031646978400
C	6.949754015487	1.548740561590	-0.005833333479
H	6.754030269432	2.173292849386	-0.881576399958
H	8.003714478146	1.276243060335	-0.006865884728
H	6.751806546222	2.157830142037	0.880249551574

N	3.912744335194	1.556120533071	-0.005534296714
H	4.251611423756	2.506825909383	0.005310727507
N	0.290890033714	-0.049396149296	-0.020460503195
C	2.620018730309	1.204610055614	-0.009454459332
C	2.533873612815	-0.234522582881	-0.025578025115
C	-3.429773011540	3.272201412785	-0.020546752341
H	-4.046057342302	3.501607160782	0.851157182942
H	-4.015288512084	3.515343987968	-0.909724074288
H	-2.556498596433	3.919237772344	-0.000302271995
C	0.417176289710	1.287878758738	-0.008852458180
H	-0.688599054407	3.026257950341	-0.000371364836
O	-1.379494679396	-1.996449455247	-1.659727916387
H	-2.020864083775	-2.714261380110	-1.768561862576
O	-1.437832665711	-1.948945781642	1.668104973827
H	-2.247631309439	-2.130554952169	2.167256980654
H	-0.504369349096	-2.412193918441	-1.675389035501
H	-0.801748715102	-1.589112409497	2.303728157534

Table S27 Cartesian coordinates and energy values of $[Zn(LH)(H_2O)_2]^{2+}$ (N^4).

Sum of electronic and thermal Energies (Eh)	-2988.6050
Sum of electronic and enthalpy Energies (Eh)	-2988.6041
Sum of electronic and thermal Free Energies (Eh)	-2988.6828
Number of Imaginary Frequencies	0

Molecular Geometry in Cartesian Coordinates

Zn	8.391837032710	6.541684978103	10.531534491673
N	4.371999931631	5.537435449831	7.928219484709
C	10.073538268686	9.264373257542	14.157048056577
H	10.407751093996	9.898530831184	14.967231519945
C	5.460284403391	2.072097805419	5.057903523158
H	4.876752352158	1.554870049187	4.308180562750
C	10.931771380760	8.343925190085	13.585465391038
H	11.951788458618	8.227070503340	13.922539696933
N	5.653909690435	7.588009767584	10.484247615695
N	6.793143236596	7.758439242790	11.152306198362
N	6.762122747749	5.925196406738	9.316132430722
C	10.457931800188	7.555492173024	12.548288358847
H	11.088274500210	6.821632560762	12.062345445643
N	9.213674965559	7.655967195141	12.090801333844
C	8.366596419296	8.546902292437	12.645631599381
C	6.991445576884	3.334973385633	6.933252265972
C	7.592901844433	2.323316385318	6.217146792518
C	4.883636581217	3.091569021749	5.791988666622
H	3.854924564483	3.382116602668	5.629562373216
C	5.660028818842	3.732741433830	6.742861783206
C	8.775169520946	9.367501428080	13.680638362731
H	8.092661752817	10.080405061840	14.117909927918
C	6.995993066189	8.599510526003	12.094876414379
C	6.786615259670	1.701535128241	5.271249506744
H	7.208523621372	0.898780176069	4.679333774005
C	9.017203244234	1.920379293519	6.442436741448
H	9.694020515771	2.762454950065	6.274943749095
H	9.298565544792	1.114189928852	5.767144715551
H	9.169911870704	1.578630635324	7.469690598005
N	7.566937501949	4.129913815661	7.945125149578

H	8.510192269137	4.051560696916	8.296696799452
N	4.465083880085	6.470517494715	8.876405045115
C	6.650421978561	4.997766600807	8.385208968496
C	5.427932385665	4.801562431263	7.671718014492
C	6.000497155322	9.562334023355	12.634797081603
H	6.361813189846	10.584811519762	12.504719561495
H	5.860198111352	9.397046448777	13.704754792163
H	5.032318559028	9.479986622559	12.148729688366
C	5.623636460251	6.621109613125	9.517256579808
H	4.816925907194	8.126036565480	10.671477177386
O	9.643451069264	7.125684372510	8.996391710717
H	10.573157384033	7.244126119415	9.241329264071
O	8.928195098584	4.591152722414	11.009187497669
H	9.820261745069	4.487068388140	11.372464994029
H	9.640024747829	6.541739795343	8.223212298117
H			

The ability of VLX600 to induce ROS production (DCFH/DA assay)

To monitor the cellular levels of ROS, SW480 and CH1/PA-1 cells were seeded into 96-well plates (2.5 x 10^4 cells/well) in volumes of 100 µL per well. After a 24 h pre-incubation, the cells were washed with 200 µl Hanks' balanced salt solution (HBSS, Sigma-Aldrich, containing 1% FBS) and incubated with 100 µL of 25 µM DCFH-DA (2',7'-dichlorofluorescein diacetate, Sigma-Aldrich) in HBSS (containing 1% FBS) for 45 min at 37 °C. After the incubation period, DCFH-DA solution was removed, and cells were washed with 200 µL HBSS. Cells were exposed to VLX600 alone and in the presence of 1 equiv. $CuCl_2$, 0.5 equiv. $ZnCl_2$ and 0.5 equiv. $FeCl_3$ at four different concentrations between the range of 0.2 and 25 µM diluted in 200 µL phenol-red-free Opti-MEM (Gibco; with 1% FBS) and applied as triplicates. As blank, phenol-red-free medium with 1% FBS was used; the negative controls were the non-drug-treated cells in phenol-red-free medium with 1% FBS and the positive controls were cells treated with 200 µM and 400 µM tert-butyl hydroperoxide (Sigma-Aldrich). Fluorescence emission was measured at 10 min intervals with the BioTek Synergy HT reader (excitation: 485/20 nm, emission: 516/20 nm) over 2 h.

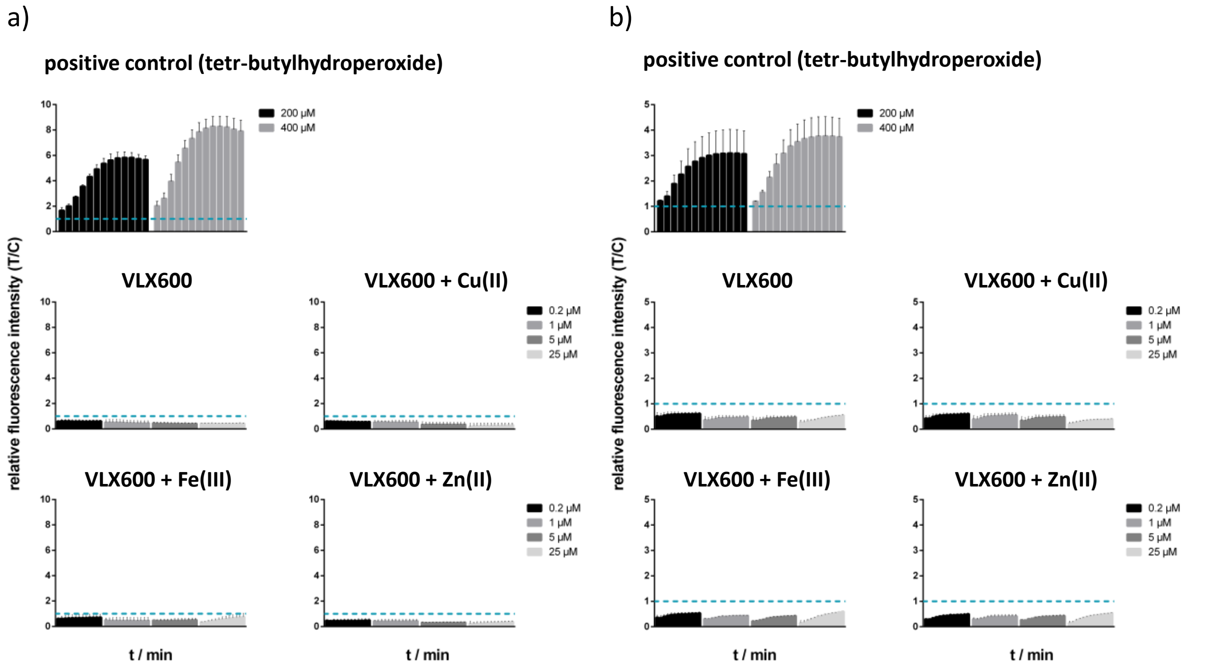

Figure S14 a) Levels of ROS in CH1/PA-1 and b) SW480 cancer cell lines treated with VLX600 in the absence and in the presence of 1 equiv $CuCl_2$, 0.5 eq. $ZnCl_2$ and 0.5 eq. $FeCl_3$ (2 h). (Positive control: tert-buthylhydroperoxide)

NMR spectra of VLX600

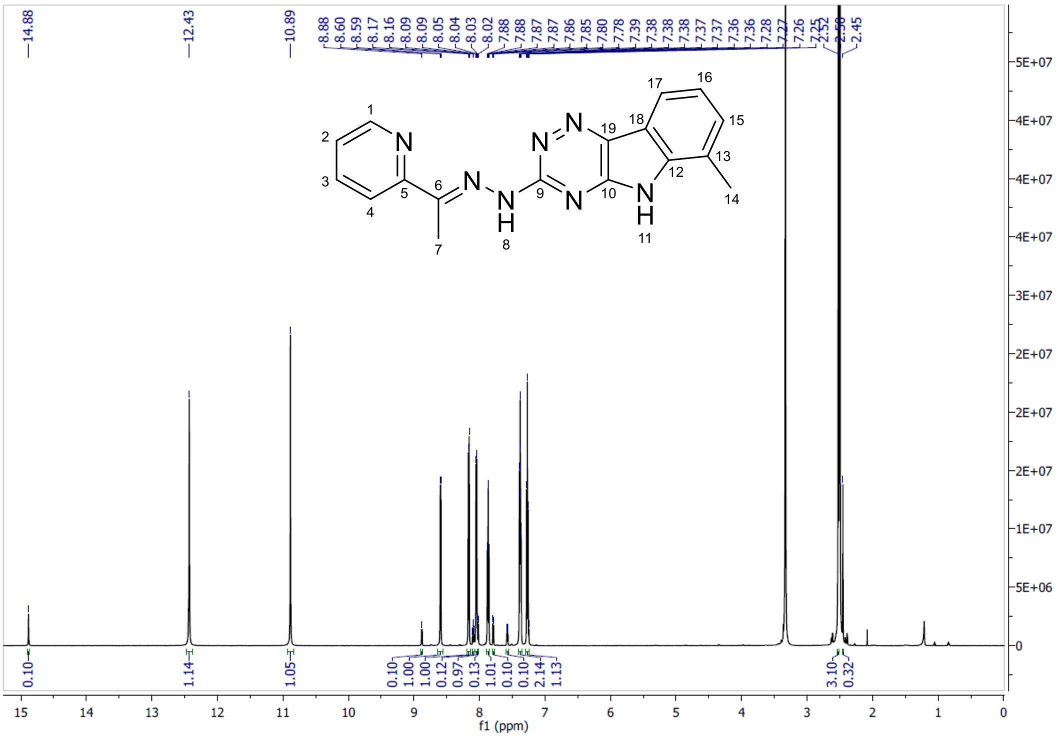

Figure S15 ¹H NMR spectra of VLX600 in DMSO-d_6 at 600 MHz.

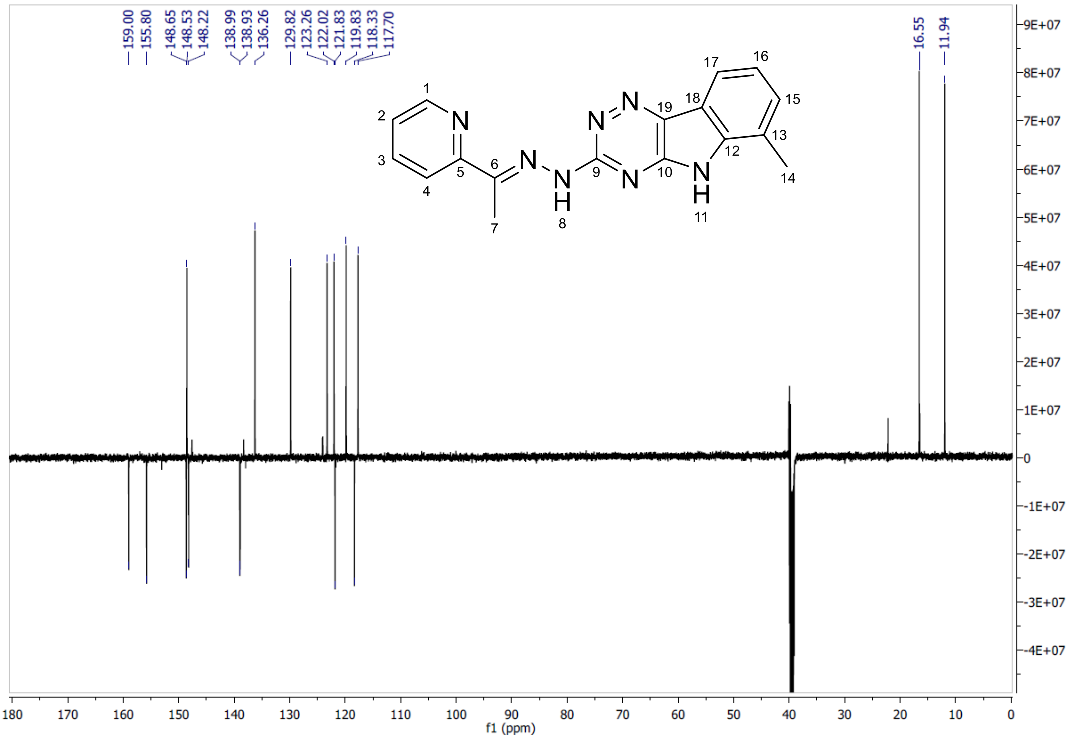

Figure S16 ¹³C NMR spectra of VLX600 in DMSO-d_6 at 151 MHz.
